# Diagnostic accuracy of phosphorylated tau217 in detecting Alzheimer's disease pathology among cognitively impaired and unimpaired: A systematic review and meta‐analysis

**DOI:** 10.1002/alz.14458

**Published:** 2024-12-23

**Authors:** Mohammad Khalafi, William J. Dartora, Laura Beth J. McIntire, Tracy A. Butler, Krista M. Wartchow, Seyed Hani Hojjati, Qolamreza R. Razlighi, Kiarash Shirbandi, Liangdong Zhou, Kewei Chen, Ke Xi, Samprit Banerjee, Nancy Foldi, Silky Pahlajani, Lidia Glodzik, Yi Li, Mony J. de Leon, Gloria C. Chiang

**Affiliations:** ^1^ Department of Radiology Brain Health Imaging Institute Weill Cornell Medicine New York New York USA; ^2^ Lipidomics and Biomarker Discovery Lab Department of Radiology Brain Health Imaging Institute Weill Cornell Medicine New York New York USA; ^3^ Biomedical Engineering Department Science and Research Branch Islamic Azad University Tehran Iran; ^4^ Banner Alzheimer's Institute Banner Health Phoenix Arizona USA; ^5^ College of Health Solutions Arizona State University Phoenix Arizona USA; ^6^ Department of Population Health Sciences Weill Cornell Medicine New York New York USA; ^7^ Department of Neurology Weill Cornell Medicine New York New York USA

**Keywords:** Alzheimer's disease, amyloid beta positron emission tomography imaging, cerebrospinal fluid biomarkers, diagnostic test accuracy, phosphorylated tau 217, plasma biomarkers, positron emission tomography, tau positron emission tomography imaging

## Abstract

Our review summarizes the diagnostic accuracy of plasma and cerebrospinal fluid (CSF) phosphorylated tau 217 (p‐tau217) in detecting amyloid and tau pathology on positron emission tomography (PET). We systematically reviewed studies that reported the diagnostic accuracy of plasma and CSF p‐tau217, searching MEDLINE/PubMed, Scopus, and Web of Science through August 2024. The accuracy of p‐tau217 in predicting amyloid and tau pathology on PET was evaluated in 30 studies. Both plasma and CSF p‐tau217 effectively detect amyloid and tau PET deposition. Plasma p‐tau217 showed 82% sensitivity for detecting amyloid and 83% for tau, with 86% and 83% specificity, respectively. CSF p‐tau217 had 79% sensitivity for amyloid and 91% for tau, with 91% and 84% specificity. p‐tau217 effectively identifies Alzheimer's disease (AD) pathology. Plasma p‐tau217 was comparable to CSF p‐tau217 in detecting amyloid deposition on PET. Despite being less sensitive for detecting tau deposition on PET, plasma p‐tau217 can be an efficient screening tool for underlying AD pathology.

**Highlights:**

- Plasma phosphorylated tau 217 (p‐tau217) serves as a viable biomarker alternative to cerebrospinal fluid p‐tau217 due to the strong concordance between their results.
- Plasma p‐tau217 accurately identifies amyloid and tau positron emission tomography (PET) positivity, exhibiting a low rate of false negatives and positives, thereby establishing it as a reliable diagnostic tool for Alzheimer's disease (AD).
- Plasma p‐tau217 demonstrates slightly higher accuracy in predicting amyloid PET positivity compared to tau PET positivity.
- Plasma p‐tau217 demonstrates higher predictive accuracy in detecting AD pathology among cognitively impaired individuals, compared to cognitively unimpaired individuals, suggesting its enhanced utility as a diagnostic biomarker in clinical settings.

## 1 BACKGROUND

Novel fluid biomarkers have shown promising results in detecting Alzheimer's disease (AD) pathology across the clinical spectrum.^1^, ^2^ Studies have found that individuals with AD have alterations in the levels of amyloid beta (Aβ), total tau (t‐tau), and phosphorylated tau (p‐tau) proteins in cerebrospinal fluid (CSF) and plasma. These alterations can effectively differentiate AD from cognitively healthy individuals.^1^, ^3^, ^4^, ^5^, ^6^ Fluid biomarkers can improve the specificity of AD clinical diagnosis by detecting biological evidence of AD, especially readily accessible plasma, which may facilitate earlier diagnosis and access to new disease‐modifying therapeutics.^7^, ^8^, ^9^

Amyloid and tau positron emission tomography (PET) scans have increasingly been used in recent years to detect and quantify accumulation of Aβ and tau pathology in the brain *ante mortem*. In addition, fluid biomarkers, including in CSF and plasma, have shown promise in detecting AD pathology and can be measured using immunoassays and mass spectrometry.^10^ Tau protein has many isoforms and is highly phosphorylated at many sites, based on age and disease state. p‐tau has different epitopes; up to 30 tau phosphorylation sites have been identified and > 15 tested in AD.^11^ Among these, phosphorylated tau 217 (p‐tau217) has shown greater accuracy than other p‐tau markers, such as plasma and CSF p‐tau181, in differentiating AD from elderly controls and other neurodegenerative disorders.^12^ Additionally, plasma p‐tau217 has been found to have diagnostic performance comparable to CSF p‐tau217.^13^, ^14^ This suggests that plasma p‐tau217 may reduce the need for invasive lumbar punctures without compromising accuracy in diagnosing AD.^15^, ^16^ Despite plasma biomarkers having not yet been approved for use in clinical settings, they are anticipated to be simpler, more cost‐effective, and easier‐to‐implement alternatives to CSF and neuroimaging biomarkers.^17^

An early, precise, and biologically based diagnosis of AD will gain further prominence given the recent advent of disease‐modifying treatments.^18^ Meta‐analyses offer a powerful approach for evaluating diagnostic accuracy by synthesizing data across multiple studies and sites, which enhances the precision of effect size estimates and broadens the generalizability of findings.^19^, ^20^, ^21^

Given the promising potential of p‐tau217, we conducted a comprehensive review of studies that evaluated its effectiveness in detecting AD pathology using Aβ and tau PET scans as reference standards. Our meta‐analysis had two primary objectives: first, we sought to assess the diagnostic test accuracy (DTA) of p‐tau217 in identifying AD pathology among cognitively impaired (CI) and cognitively unimpaired (CU) individuals; second, we explored the efficacy of various assay platforms for p‐tau217 to understand how different tests perform in clinical and research settings. We comprehensively evaluated the DTA of p‐tau217 across different assays and populations. This pooled analysis enables us to uncover differences in test performance across heterogeneous cohorts, which may not be apparent in head‐to‐head comparisons of assays within a single cohort. Ultimately, our findings aim to inform and optimize the diagnostic use of p‐tau217 in clinical practice, offering insights into its application across varied populations and assay methods.

## 2 METHODS

### 2.1 Search strategy

**FIGURE 1 alz14458-fig-0001:**
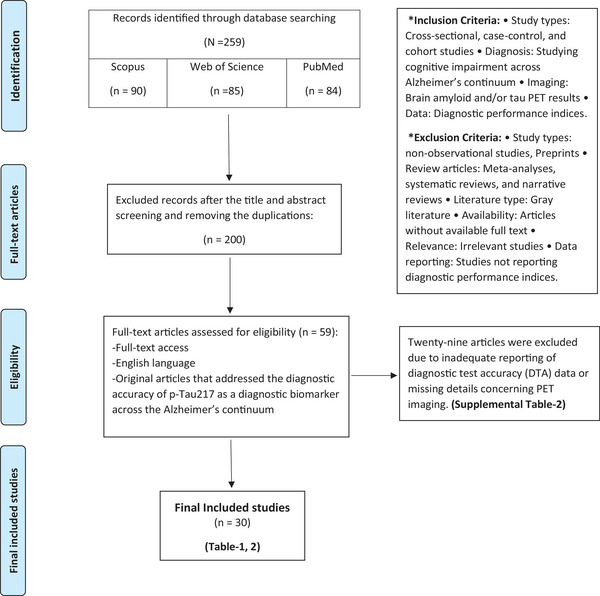
PRISMA flowchart. PET, positron emission tomography; PRISMA, Preferred Reporting Items for Systematic Reviews and Meta‐analysis; p‐tau, phosphorylated tau.

**FIGURE 2 alz14458-fig-0002:**
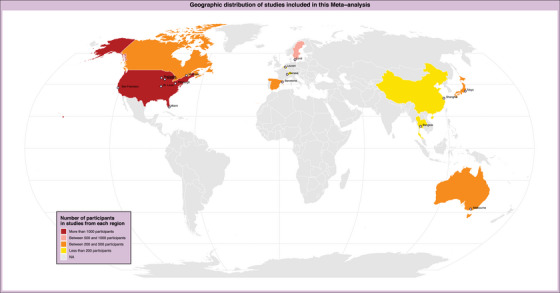
p‐tau217 has been proposed to be the most accurate fluid biomarker of AD pathology among various ethnic, racial, and clinical groups.^23^ The map illustrates the geographical distribution of included studies, highlighting the countries of origin of the cohorts included in this meta‐analysis and the number of individuals reported in the included studies, which were from the United States (MCSA, WRAP, UCSF Memory and Aging Center, Pittsburgh ADRC, Mayo Clinic ADRC, Florida ADRC),^11^, ^14^, ^18^, ^24^, ^25^, ^26^, ^27^, ^28^, ^29^, ^30^ Canada (Triad),^10^, ^18^, ^31^, ^32^ Sweden (Biofinder),^1^, ^2^, ^12^, ^18^, ^33^, ^34^, ^35^, ^36^ Switzerland (Memory Center),^15^ Belgium (F‐PACK),^37^ Spain (ALFA),^38^, ^39^ China (community‐based longitudinal cohort),^40^ Thailand (Memory Clinic),^41^ Japan (J‐TRC),^6^ and Australia (AIBL, ADNeT).^16^, ^42^ AD, Alzheimer's disease; ADNeT, Australian Dementia Network; ADRC, Alzheimer's Disease Research Center; AIBL, Australian Imaging, Biomarker & Lifestyle Flagship Study of Ageing; ALFA, Alzheimer's and Families; F‐PACK, Flemish Prevent Alzheimer's Disease Cohort KU Leuven; MCSA, Mayo Clinic Study on Aging; p‐tau217, phosphorylated tau 217; UCSF, University of California San Francisco, WRAP, Wisconsin Registry for Alzheimer's Prevention.

After registration in PROSPERO—CRD42024503676—the Preferred Reporting Items for Systematic Reviews and Meta‐analysis (PRISMA) statement was followed during a thorough literature review to cover all relevant studies (Figure 1).^22^ The review aimed to include observational studies examining the diagnostic precision of p‐tau217 as a biomarker for diagnosing AD, using tau PET and Aβ PET as the reference standards. The search was performed on four online databases, including MEDLINE/PubMed, Scopus, and Web of Science, from their inception to August 2024, using relevant keywords such as “Alzheimer's disease,” “primary senile degenerative dementia,” “presenile dementia,” “preclinical dementia,” “senile dementia,” “p‐tau217,” and “PET scan.” A search strategy was developed for each database using a combination of keywords with appropriate Boolean operators (OR/AND). All available literature was retrieved without any restrictions based on publication time, study design, language, or country of publication. Our meta‐analysis of 30 studies on p‐tau217 biomarkers spans diverse global regions, including North America, Europe, Asia, and Oceania (Figure 2).

### 2.2 Inclusion and exclusion criteria

The search identified studies of different types, including cross‐sectional, case–control, and cohort studies, which were required to meet the following criteria to be included in our analysis. The study had to include participants that were ≥ 18 years, have brain amyloid and/or tau PET results, and have reported diagnostic performance indices including sensitivity and specificity. The analysis excluded any non‐observational studies, such as case reports/series, editorials, comments, correspondence, guidelines, experimental, and interventional studies, as well as meta‐analysis and systematic and narrative reviews. Additionally, gray literature, articles without available full text, irrelevant studies, and studies that did not report diagnostic performance indices were excluded. We reached out to authors of excluded studies for supporting data and added those that provided DTA data from their study to the final inclusion list for this meta‐analysis.

**RESEARCH IN CONTEXT**

1. **Systematic review**: We conducted a literature review using MEDLINE/PubMed, Scopus, and Web of Science, focusing on studies evaluating the diagnostic accuracy of plasma and cerebrospinal fluid (CSF) phosphorylated tau (p‐tau217) to detect Alzheimer's disease (AD) pathology in both cognitively impaired and unimpaired cohorts. Our meta‐analysis is the first to report the accuracy of p‐tau217 in diverse cohorts.
2. **Interpretation**: p‐tau217 is an effective biomarker of AD pathology. Plasma p‐tau217 has comparable accuracy to CSF p‐tau217 in detecting amyloid deposition on positron emission tomography (PET), although it is slightly less sensitive in detecting tau deposition on PET. Nonetheless, plasma p‐tau217 can be a useful screening tool for detecting underlying AD, particularly with higher predictive value in cognitively impaired individuals, highlighting its promise as a diagnostic biomarker in clinical settings.
3. **Future directions**: Future studies should focus on larger, community‐based cohorts to assess p‐tau217's diagnostic accuracy in people with mixed pathologies. Standardization of methods and further investigation into specific p‐tau217 ratios, such as p‐tau217/total tau, is recommended to enhance diagnostic precision.

### 2.3 Study selection process

EndNote 21 software (Clarivate Analytics) was used to import the citations, and any duplicate entries were eliminated. The first screening stage involved two reviewers (M.K. and K.S.) who evaluated the titles and abstracts of studies to identify eligible ones. The second screening stage involved the same reviewers analyzing the selected articles’ full text, using inclusion and exclusion criteria.[Fig alz14458-fig-0001], [Fig alz14458-fig-0002]

### 2.4 Data extraction

Two investigators (M.K. and K.S.) thoroughly reviewed the studies included in this final research sample. Using a predefined Microsoft Excel worksheet, they gathered a comprehensive set of information including the first author's name, study period, country of origin, dataset source, total number, age, sex, Mini‐Mental State Examination, details of the PET method used, and statistical data findings such as true positive (TP), true negative (TN), false positive (FP), and false negative (FN). If TP, TN, FP, and FN rates were not reported in each study, the investigators calculated these figures based on the data reported and the number of participants involved. Any decimal figures were rounded to the nearest whole number.

### 2.5 Quality assessment

We used the Quality Assessment of Diagnostic Accuracy Studies (QUADAS‐2) to evaluate the standard of the studies that were included.^43^ QUADAS‐2 is a widely used tool to assess the quality of non‐randomized studies, focusing on four key domains: patient selection, index test, reference standard, and flow and timing. Each domain is systematically evaluated for potential biases and categorized as having low, high, or unclear risk. Additionally, the tool assesses concerns regarding the applicability of the study in terms of patient selection, index tests, and reference standards. The risk of bias assessment involves answering signaling questions within each domain, which helps guide the evaluator in determining the level of bias present. The decisions made during this process, along with specific criteria for each domain, are crucial for interpreting the results shown in Table S1 in supporting information and for understanding the quality assessments applied throughout the manuscript.

### 2.6 Quantitative meta‐analysis and statistical analysis

**FIGURE 3:**
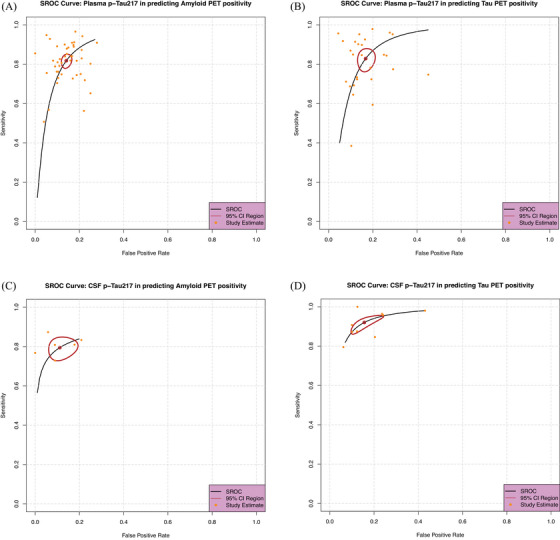
The SROC curve evaluates the performance of p‐tau217 in predicting PET positivity. The curve plots sensitivity against the false positive rate, summarizing the diagnostic accuracy across multiple studies. The curve includes a 95% confidence region to illustrate the uncertainty in the summary estimates. Each dot represents a study's individual sensitivity and false positive rate, with the SROC curve capturing the overall performance trend. A, SROC curve for plasma p‐tau217 in predicting amyloid PET positivity, with an AUC of 0.91 and sensitivity of 0.82 (95% CI: 0.79–0.84). B, SROC curve for plasma p‐tau217 in predicting tau PET positivity, with an AUC of 0.90 and sensitivity of 0.83 (95% CI: 0.78–0.87). C, SROC curve for CSF p‐tau217 in predicting amyloid PET positivity, with an AUC of 0.88 and sensitivity of 0.79 (95% CI: 0.73–0.84). D, SROC curve for CSF p‐tau217 in predicting tau PET positivity, with an AUC of 0.95 and sensitivity of 0.91 (sensitivity 95% CI: 0.87–0.95). AUC, area under the curve; CI, confidence interval; CSF, cerebrospinal fluid; PET, positron emission tomography; p‐tau217, phosphorylated tau 217; SROC, summary receiver operating characteristic.

The primary objective of this analysis was to assess the diagnostic performance of the studies, with a specific focus on the F1 score, which has the advantages of combining precision and recall, better accounting for FNs/FPs and imbalanced data sets. Additionally, we calculated sensitivity, specificity, the summary receiver operating characteristic (SROC) curve, and the diagnostic odds ratio (DOR). Point estimates and 95% confidence intervals (Cis) for these metrics were computed for each study to ensure consistency. A bivariate meta‐analysis of sensitivity and specificity was conducted to generate the SROC curve (Figure 3), using R version 4.1.2 (R Foundation for Statistical Computing, 2021) and RStudio version 1.4.1717. The R packages mada and meta4diag were used for this purpose.^44^, ^45^ To account for the methodological differences across diagnostic studies, we used a random effects model; this approach allows for variability between studies, accommodating potential differences in study design, sample characteristics, and measurement techniques, thereby providing a more generalized estimate of the effect size.^46^ The ggplot2^47^ library was used to generate some of the visualizations, including diagnostic performance plots. To assess potential publication bias, we used a funnel plot. All statistical tests were two sided, and a *p* value of <0.05 was considered statistically significant.

**Sensitivity (true positive rate [TPR])**: The proportion of actual positives that are correctly identified.

**Sensitivity = TP/(TP + FN)​**

**Specificity (TN Rate)**: The proportion of actual negatives that are correctly identified.

**Specificity = TN(TN + FP)**

**DOR**: The ratio of the odds of the test being positive in patients with the disease compared to those without.

​**DOR = (TP × TN)/(FP × FN)​**

**F1 Score**: A harmonic mean of precision and recall, used as the primary metric in this analysis.

**F1 score = (2** × **Precision** × **Recall)/Precision** + **Recall = (2** ×TP **)/2TP + (FP + FN)**

To plot the SROC curve, we calculated the following:

**False Positive Rate (FPR)**: The proportion of actual negatives that are incorrectly classified as positives.

**FPR = FP/(FP + TN)**

**TPR**: Equivalent to sensitivity, representing the proportion of actual positives correctly classified.

**TPR = TP/(TP + FN)**

### 2.7 Meta‐analysis heterogeneity

In a meta‐analysis, the level of variation among the included studies can be assessed by calculating the between‐study variance.^48^, ^49^ Understanding the variance among studies is essential for interpreting the differences in outcomes across meta‐analyses. In particular, the presence of heterogeneity can significantly affect the reliability of pooled estimates, making it crucial to approach the results with caution. Heterogeneity is quantified using the *I* ^2^ statistic, which measures the proportion of total variation across studies that is due to heterogeneity rather than chance. *I* ^2^ values range from 0% to 100%, with higher values indicating greater inconsistency among study results. An *I* ^2^ value between 0% and 25% indicates low or negligible heterogeneity, 25% to 50% indicates moderate heterogeneity, 50% to 75% indicates substantial heterogeneity, and 75% to 100% indicates high heterogeneity.^50^ Our analysis revealed substantial variability across both plasma and CSF studies, with *I* ^2^ values > 50% in each set, indicating moderate to high heterogeneity. Heterogeneity was also observed across most subgroups analyzed, although one subgroup exhibited a low *I* ^2^ value and a non‐significant *p* value, suggesting less variability. The observed heterogeneity in other subgroups underscores the need for careful interpretation of the findings, as high *I* ^2^ values suggest that much of the variability between study outcomes is likely influenced by differences in study design, populations, or methodologies, rather than random chance. Forest plots offer further insights into the degree of heterogeneity, including precision estimates and *p* values for each subgroup, allowing for a more detailed assessment of variability. The level of heterogeneity observed highlights the complexity of synthesizing results across diverse studies and emphasizes the importance of accounting for these differences when drawing conclusions (Figures S1–S5 in supporting information).

## 3 RESULTS

After passing the eligibility criteria of the PRISMA flow diagram, 59 studies were included. Among them, 30 studies (Tables 1 and 2), including 9787 individuals from 10 countries (Figure 2) that reported the DTA of p‐tau217 in CSF, plasma, or both, were included in the final analysis. Twenty‐nine studies were excluded due to insufficient data (Table S2 in supporting information). Data from these studies were reviewed, and the data were entered into a meta‐analysis table for quantitative analysis. We used the SROC curve to evaluate the performance of p‐tau217 in predicting PET positivity, which includes a 95% confidence region to illustrate the uncertainty in the summary estimates (Figure 3). Due to the importance of investigating FN cases and using the F1 score in medical diagnostic tests, we used the average F1 score to assess classification.^53^, [Table alz14458-tbl-0001], [Table alz14458-tbl-0002]

**TABLE 1 alz14458-tbl-0001:** Descriptive insights from studies using plasma and CSF p‐tau217 to predict amyloid PET positivity.

ID	Location	Source	Assay	Participants (AD/MCI/CN)	Age (AD/MCI/CN)	Tracer	Finding(s)	Limitation(s)
Palmqvist et al. (2020)^35^	Sweden	Plasma, CSF	Immunoassay: Lilly	(121/178/301) +99 neurodegenerative	(74/72/67)	[18F]Flutemetamol	p‐tau217 was found to have the strongest correlation with Aβ PET in the medial frontoparietal regions.	• Clinic‐based cohort. • The primary analysis excluded participants with plasma p‐tau217 concentrations that were too low to be accurately determined. • The assay being used currently is of research‐grade quality.
Suarez‐Calvet et al. (2020)^39^	Spain	CSF	Immunoassay: Simoa (Homebrew)	NA‐ (AB+ = 131, AB− = 250)	(AB+ = 62, AB− = 61)	[18F]Flutemetamol	New biomarkers, including CSF and plasma N‐p‐tau181, CSF N‐p‐tau217, and CSF Midp‐tau231, increase early in the preclinical stage of AD.	• Clinic‐based cohort. • A cross‐sectional analysis. • Various biomarkers can be measured using different analytical platforms and antibodies. • No available tau PET. • No plasma p‐tau217.
Janelidze et al. (2021)^33^	Sweden	CSF	Immunoassay: Lilly	(NA/186/314)	(NA/72/65)	[18F]Flutemetamol	In MCI, plasma p‐tau217 has the highest predictive value for Aβ status compared to other biomarkers.	Dementia‐based cohort.
Leuzy et al. (2021)^1^	Sweden	CSF	Immunoassay: Lilly	(119/84/334)	(77/71/66)	[18F]Flutemetamol	CSF p‐tau217Lilly is more beneficial than other p‐tau assays in diagnosing AD and Aβ status.	• No Aβ‐PET in the AD dementia group. • Clinic‐based cohort.
Thijssen et al. (2021)^14^	USA	Plasma	Immunoassay: Lilly	(2/1.4/1.1)	(58/99/118)	[11C]PiB [18F]Florbetapir, or [18F]Florbetaben	Both p‐tau217 and p‐tau181 are highly effective in diagnosing AD and differentiating it from other groups. P‐tau217 has a slight advantage in the differential diagnosis of clinical AD and predicting amyloid PET positivity.	• The homogeneity of the racial composition of the participants. • Young cohort.
Therriault et al. (2022)^13^	Canada	Plasma, CSF	Immunoassay: Lilly	NA‐ (CI = 71/CU = 113)	(CI = 69/CU = 71)	[18F]AZD4694	Plasma and CSF p‐tau217 have similar diagnostic performance for AD and Aβ status. Plasma p‐tau217 can reduce the need for invasive lumbar punctures without compromising accuracy in identifying AD.	• Single‐center study • Clinic‐based cohort.
Dore et al. (2022)^42^	Australia	Plasma	Immunoassay: Simoa (Janssen R&D)	(83/91/223)	(71/74/75)	[18F]NAV4694	An elevated level of plasma p‐tau217 is linked with elevated Aβ in predicting AD pathology.	Clinic‐based cohort.
Mila‐Aloma et al. (2022)^38^	Spain	Plasma	Immunoassay: Lilly	NA‐ (Aβ+ = 135/Aβ− = 262)	(Aβ+ = 62/ Aβ− = 61)	[18F]Flutemetamol	p‐tau231 and p‐tau217 will aid in recruiting participants for early‐stage clinical trials for anti‐amyloid therapy and predicting Aβ status.	• The role of assay platforms in determining the accuracy of diagnostic tests for plasma biomarkers is uncertain. • Clinic‐based cohort.
Meilke et al. (2022)^26^	USA	Plasma	Immunoassay: Lilly	(6/81/666)	(84/77/72)	[11C]PIB	p‐tau217 was found to be a more reliable predictor of amyloid PET compared to P‐tau181.	The homogeneity of the racial composition of the participants.
Brum et al. (2023)^51^	Sweden	Plasma	Immunoassay: Lilly	(NA/348/NA)	(NA/73/NA)	[18F]Flutemetamol	Screening MCI patients for Aβ positivity and using a plasma p‐tau217‐based model can accurately classify patients while reducing the need for costly or invasive Aβ tests.	Biomarker measurements were done in a single‐batch manner. Aβ PET was used as the reference standard for evaluation.
Woo et al. (2023)^31^	Canada	Plasma	Immunoassay: Simoa (Janssen R&D)	NA‐ (PET+ = 89/PET− = 145)	(PET+ = 65 to 71/PET− = 42)	[18F]AZD4694	Combining plasma levels of p‐tau217 and N‐terminal‐tau can sensitively identify PET positivity in individuals with Aβ+.	NA
Xiao et al. (2023)^40^	China	Plasma	Immunoassay: Simoa (ALZpath)	NA‐ (Aβ+ = 10/Aβ− = 61)	(Aβ+ = 63/ Aβ− = 63)	[18F]Florbetapir	Plasma p‐tau217 may be an early predictive marker of AD and Aβ pathology in older individuals residing in the community.	Small sample size.
VandeVrede et al. (2023)^28^	USA	Plasma	Immunoassay: Lilly	(54/NA/59) + other participants	(65/NA/67)	[11C]PIB [18F]Florbetapir, [18F]Florbetaben	Plasma p‐tau217 can be an inexpensive tool to predict amyloid PET positivity and differentiate AD from other neurodegenerative diseases.	The study has three main issues: unequal diagnostic groups, reliance on PET scans to define AD, and the absence of a replication cohort.
Jonaitis et al. (2023)^25^	USA	Plasma	Immunoassay: Lilly	NA‐ (PET+ = 124/PET− = 206)	68	[11C]PiB	p‐tau217 can detect AD and predict Aβ status before symptoms appear, differentiating it from normal cognitive aging.	• Clinic‐based cohort. • Small sample size. • The homogeneity of the racial composition of the participants.
Rissman et al. (2023)^27^	USA	Plasma	Mass spectrometry (LC‐MS/MS)	NA‐ (Aβ+ = 720, Aβ− = 340)	(Aβ+ = 70, Aβ− = 67)	[18F]NAV4694	• P‐tau217/Nptau217 ratio improves plasma biomarker algorithms for preclinical amyloid PET positivity. • Prediction performance at higher NAV centiloid levels was improved with p‐tau217/np‐tau217	Participants’ homogenous racial composition.
Jack et al. (2023)^24^	USA	Plasma	Immunoassay: Lilly	(124/148/864)	(70/75/70)	[11C]PiB	The Lilly p‐tau217 assay compared well to the Quanterix p‐tau181 assay. But Quanterix's four biomarkers performed better. Staging amyloid pathology may become more important in AD clinical trials.	NA
Mendes et al. (2024)^15^	Switzerland	Plasma, CSF	Immunoassay: Lilly	(14, 67, 33)	(71 ± 9/73 ± 6/69 ± 8)	[18F]Flutemetamol or [18F]Florbetapir	Plasma p‐tau217 outperforms other variants of p‐tau in identifying Aβ status and clinical phenotypes. p‐tau217 shows similar performance in both plasma and CSF.	• Clinic‐based cohort. • Small sample size.
Feizpour et al. (2024)^16^	Australia	Plasma	Immunoassay: Simoa (Janssen R&D)	NA‐ (PET+ = 283/PET− = 192)	(PET+ = 65 to 74±8/PET− = 74±6)	[18F]NAV4694	Initial and early p‐tau217 shows efficacy in predicting Aβ status.	Clinic‐based cohort.
Ashton et al. (2024)^18^	USA, Canada	Plasma	Immunoassay: Simoa (ALZpath)	NA‐ WRAP: (PET+ = 58/PET− = 209) TRIAD: (PET+ = 122/PET− = 146)	WRAP: (65) TRIAD: (69)	[18F]AZD4694, [11C]PIB	p‐tau217 test can reduce confirmatory testing for Aβ positivity by 80% in the AD continuum.	Clinic‐based cohort.
Barthelemy et al. (2024)^2^	USA, Sweden	Plasma	Mass spectrometry (LC‐MS/HRMS)	(349/403/1007)	70	[18F]Flutemetamol or [18F]Florbetapir,	Blood %p‐tau217 implementation would reduce the need for PET or CSF testing, enhance access to accurate AD diagnosis, and enable determination of amyloid status in patients with MCI or mild dementia who might benefit from anti‐amyloid immunotherapies.	• Clinic‐based cohort. • Mass spectrometry is more expensive and requires more technical expertise than immunoassays.
Therriault et al. (2024)^32^	Canada	Plasma	Immunoassay: Simoa (Jansson & ALZpath)	(PET+ = 121/PET− = 148)	(PET+ = 70/PET− = 67)	[18F]AZD4698	Both plasma p‐tau217 assays were closely linked to amyloid and tau PET and effectively distinguished AD from other neurodegenerative diseases. Plasma p‐tau217 levels increased with AD severity and were tracked with annual tau PET changes.	• Clinic‐based cohort. • Participants’ homogenous racial composition.
Niimi et al. (2024)^6^	Japan	Plasma	Immunoassay: Lilly	(PET+ = 81/PET− = 393)	(PET+ = 74±6/PET− = 71±7)	[18F]flutemetamol	The combination of plasma Aβ markers and p‐tau217 has shown high accuracy in detecting Aβ PET positivity in preclinical and prodromal AD in Japanese and Swedish cohorts.	• Clinic‐based cohort. • Small sample size.
Thanapornsangsuth et al. (2024)^41^	Thailand	Plasma	Immunoassay: Lilly	(AD = 72/60 = non‐AD)	(68/67)	[18F]florbetaben	Incorporating pretest probability into plasma p‐Tau217 interpretation significantly enhances diagnostic performance, potentially eliminating the need for confirmatory tests in most cases.	• Clinic‐based cohort. • Small sample size.
Asken et al. (2024)^29^	USA	Plasma	Immunoassay: Simoa (ALZpath)	(PET+ = 108/PET− = 161)	71 ± 8	[18F]florbetapir, or [18F]florbetaben	Plasma p‐tau217 strongly predicts elevated brain Aβ on Aβ PET in both Hispanic/Latino and non‐Hispanic/Latino older adults. A two‐cutoff “intermediate‐range” approach could reduce the need for costly and invasive confirmatory CSF or PET tests.	• No longitudinal follow‐up. • No tau PET.
Figdore et al. (2024)^30^	USA	Plasma	Immunoassay: Simoa (ALZpath) and Lumipulse, Fujirebio	(82/345/NA)	(76/79/NA)	[11C]PiB	The study best DTA with a two‐cutpoint approach. The assays showed significant differences in intermediate classifications, providing valuable insights for the clinical interpretation of these biomarkers in AD.	NA
de Meyer et al. (2024)^37^	Belgium	Plasma	Immunoassay: Simoa (ALZpath)	(PET+ = 16/PET− = 59)	(PET+ = 71±4/PET− = 69±6)	[11C]PiB, [18F]flutemetamol	Baseline plasma p‐Tau217 predicts Aβ accumulation in asymptomatic AD as well as Aβ PET. In a multimodal model, the predictive value diminishes, indicating overlapping information.	• Clinic‐based cohort. • Small sample size. • Two different PET tracers.

Abbreviations: Aβ, amyloid beta; AD, Alzheimer's disease; CI, cognitively impaired; CN, cognitively normal; CSF, cerebrospinal fluid; CU, cognitively unimpaired; HRMS, high‐resolution mass spectrometry; LC‐MS/MS, liquid chromatography tandem mass spectrometry; MCI, mild cognitive impairment; PET, positron emission tomography; PiB, Pittsburgh compound B; p‐tau, phosphorylated tau.

**TABLE 2 alz14458-tbl-0002:** Descriptive insights from studies using plasma and CSF p‐Tau217 to predict tau PET positivity.

ID	Location	Source	Assay	Participants (AD/MCI/CN)	Age (AD/MCI/CN)	Tracer	Finding(s)	Limitation(s)
Palmqvist et al. (2020)^35^	Sweden	Plasma, CSF	Immunoassay: Lilly	(121/178/301) +99 neurodegenerative	(74/72/67)	[18F]RO948,	The levels of p‐tau217 were found to be highly correlated with the tau PET signal in the temporoparietal regions.	• Clinic‐based cohort. • The primary analysis excluded participants with plasma p‐tau217 concentrations that were too low to be accurately determined. • The assay being used currently is of research‐grade quality.
Janelidze et al. (2020)^12^	Sweden	CSF	Immunoassay: Lilly	(43/29/65)	(72/72/75)	[18F]Flortaucipir	P‐tau217 may be more useful than P‐tau181 in diagnosing AD and tau status, as validated by a different immunoassay.	Clinic‐based cohort.
Janelidze et al. (2021)^33^	Sweden	CSF	Immunoassay: Lilly	(NA/186/314)	(NA/72/65)	[18F]RO948	In MCI, plasma p‐tau217 has a higher predictive value for tau status than other biomarkers.	Clinic‐based cohort.
Leuzy et al. (2021)^1^	Sweden	CSF	Immunoassay: Lilly	(119/84/334)	(77/71/66)	[18F]RO948	CSF p‐tau217Lilly is more beneficial than other p‐tau assays in detecting tau PET positivity.	• No Aβ PET in the AD dementia group. • Clinic‐based cohort.
Thijssen et al. (2021)^14^	USA	Plasma	Immunoassay: Lilly	(2/1.4/1.1)	(58/99/118)	[18F]Flortaucipir	Both p‐tau217 and p‐tau181 are highly effective in diagnosing AD and predicting tau PET positivity.	• The homogeneity of the racial composition of the participants. • Young cohort.
Ossenkoppele et al. (2021)^34^	Sweden, ADNI	CSF	Immunoassay: Lilly	(NA/310/461)	(NA/72/65)	[18F]RO948, [18F]Flortaucipir	CSF and plasma p‐tau181/217 are linked with early AD markers, while tau PET strongly correlates with cognitive and neurodegenerative markers of disease progression.	• p‐au217 was unavailable in ADNI at the time of tau PET, and CSF p‐tau217 was not determined. • Clinic‐based cohort.
Therriault et al. (2022)^13^	Canada	Plasma, CSF	Immunoassay: Simoa (Janssen R&D)	NA‐ (CI = 71/CU = 113)	(CI = 69/CU = 71)	[18F]MK‐6240	Individuals with CDR ≥ 1 or tau PET positivity had no plasma/CSF p‐tau217 discordance.	• Single‐center study • Clinic‐based cohort.
Dore et al. (2022)^42^	Australia	Plasma	Immunoassay: Simoa (Janssen R&D)	(83/91/223)	(71/74/75)	[18F]MK‐6240	An elevated level of plasma p‐tau217 is linked with elevated Aβ in predicting AD pathology.	Clinic‐based cohort.
Meilke et al. (2022)^26^	USA	Plasma	Immunoassay: Lilly	(6/81/666)	(84/77/72)	[18F]Flortaucipir	Tau PET predicted P‐tau181 and P‐tau217 for ERC tau PET accurately (AUROC > 0.80), but less so for a tau PET temporal meta‐ROI with ERC (AUROC < 0.70).	The homogeneity of the racial composition of the participants.
Woo et al. (2023)^31^	Canada	Plasma	Immunoassay: Simoa (Janssen R&D)	NA‐ (PET+ = 89/PET− = 145)	(PET+ = 65 to 71/PET− = 42)	[18F]MK‐6240,	Combining plasma levels of p‐tau217 and N‐terminal‐tau can sensitively identify tau PET positivity in individuals with Aβ+.	NA
VandeVrede et al. (2023)^28^	USA	Plasma	Immunoassay: Lilly	(54/NA/59) + other participants	(65/NA/67)	[18F]Flortaucipir	Plasma p‐tau217 has shown outstanding diagnostic performance in identifying tau PET positivity in AD pathology.	The study has three main issues: unequal diagnostic groups, reliance on PET scans to define AD, and the absence of a replication cohort.
Jonaitis et al. (2023)^25^	USA	Plasma	Immunoassay: Lilly	NA‐ (PET+ = 124/PET− = 206)	68	[18F]MK‐6240	p‐tau217 can detect AD before symptoms appear, differentiating it from normal cognitive aging.	• Clinic‐based cohort. • Small sample size. • The homogeneity of the racial composition of the participants.
Gonzalez‐Ortiz et al. (2023)^52^	Sweden, USA	Plasma	Mass spectrometry (IP‐MS), Simoa	NA‐ (AB+ = 72, AB− = 21)	(AB+ = 79, AB− = 63)	[18F]RO948	p‐tau217 identifies individuals with abnormal tau PET scans well and strongly associates with cognitive performance and other biomarkers.	• Lack of neuropathology. • Clinic‐based cohort. • Small sample size.
Mattsson‐Carlgren et al. (2024)^36^	Sweden	Plasma	Immunoassay: Lilly	NA‐ (PET+ = 72, PET− = 21)	(Aβ+ = 79, Aβ− = 63)	[18F]Flortaucipir, [18F]RO948	p‐tau217 measurement can reduce the need for invasive and costly tests to identify candidates for anti‐amyloid therapies.	Clinic‐based cohort.
Mendes et al. (2024)^15^	Switzerland	Plasma, CSF	Immunoassay: Lilly	(14, 67, 33)	(71 ± 9/73 ± 6/69 ± 8)	[18F]Flortaucipir	Plasma p‐tau217 outperforms other variants of p‐tau in identifying AD pathology and clinical phenotypes in a memory clinic cohort. p‐tau217 shows similar performance in both plasma and CSF.	• Clinic‐based cohort. • Small sample size.
Feizpour et al. (2024)^16^	Australia	Plasma	Immunoassay: Simoa (Janssen R&D)	NA‐ (PET+ = 283/PET− = 192)	(PET+ = 65 to 74±8/PET− = 74±6)	[18F]MK‐6240,	Using 18F‐MK6240 as a tau PET tracer may have provided a more optimal identification of early‐stage AD but the findings were not able to distinguish between A+T− and A+T_MTL_+ based on plasma p‐tau217.	Clinic‐based cohort.
Ashton et al. (2024)^18^	USA, Canada	Plasma	Immunoassay: Simoa (ALZpath)	NA‐ WRAP: (PET+ = 58/PET− = 209) TRIAD: (PET+ = 122/PET− = 146)	WRAP: (65) TRIAD: (69)	[18F]MK‐6240,	p‐tau217 test can reduce confirmatory testing for Aβ positivity by 80% in predicting amyloid and tau positivity.	Clinic‐based cohort.
Barthelemy et al. (2024)^2^	USA, Sweden	Plasma	Mass spectrometry (LC‐MS/HRMS)	(349/403/1007)	70	[18F]Flortaucipir, [18F]RO948	Plasma %p‐tau217 had very high performance in classifying tau PET status in BioFINDER‐2 cohort, superior to CSF Elecsys p‐tau181/Aβ42 and CSF Elecsys Aβ42/40.	• Clinic‐based cohort. • Mass spectrometry is more expensive and requires more technical expertise than immunoassays.

Abbreviations: Aβ, amyloid beta; AD, Alzheimer's disease; ADNI, Alzheimer's Disease Neuroimaging Initiative; AUROC, area under the receiver operating characteristic curve; CDR, Clinical Dementia Rating; CI, cognitively impaired; CN, cognitively normal; CSF, cerebrospinal fluid; CU, cognitively unimpaired; ERC, entorhinal cortical; HRMS, high‐resolution mass spectrometry; IP‐MS, immunoprecipitation mass spectrometry; LC‐MS/MS, liquid chromatography tandem mass spectrometry; MCI, mild cognitive impairment; PET, positron emission tomography; PiB, Pittsburgh compound B; p‐tau, phosphorylated tau; ROI, region of interest; TRIAD, Translational Biomarkers in Aging and Dementia; WRAP, Wisconsin Registry for Alzheimer's Prevention.

### 3.1 Evaluation of plasma p‐tau217 for predicting amyloid PET positivity

We evaluated 23 studies that were conducted using amyloid PET imaging to investigate the accuracy of plasma p‐tau217 in detecting Aβ pathology. The results indicated a pooled sensitivity of 82% (95% Ci: 78.5%–84.3% *I* ^2 ^= 85%), a pooled specificity of 86% (95% Ci: 84.0%–87.8% *I* ^2 ^= 81%), and an F1 score of 0.77 (95% Ci: 0.741–0.805; *I* ^2 ^= 95%). The weakest performance was observed in detecting Aβ in CU participants with a pooled sensitivity of 76% (95% Ci: 71.4%–80.3% *I* ^2 ^= 55%), a pooled specificity of 83% (95% Ci: 79%–86% *I* ^2 ^= 86%), and an F1 score of 0.67 (95%  Ci: 0.567–0.716; *I* ^2 ^= 90%; Table 3). The DTA of p‐tau217 in identifying Aβ in individuals with CI showed a pooled sensitivity of 85% (95% Ci: 81.4%–88.2%; *I* ^2 ^= 67%), a pooled specificity of 86% (95% Ci: 82.8%–88.2%; *I* ^2 ^= 4%), and an F1 score of 0.87 (95% Ci: 0.847–0.894; *I* ^2 ^= 66%). In mixed cohorts of CU and CI individuals, plasma p‐tau 217 had a pooled sensitivity of 83% (95% Ci: 77.7%–87.3%; *I* ^2 ^= 89%), a pooled specificity of 88% (95% Ci: 85.5%–90.6%; *I* ^2 ^= 80%), and an F1 score of 0.8 (95% Ci: 0.752–0.841; *I* ^2 ^= 90%) in detecting Aβ (Table 3). The measurement of p‐tau217 showed slight variations when different amyloid radiotracers were used as the reference standard. [11C] Pittsburgh compound B and [18F] Flutemetamol are more widely reported, and their combined F1 scores were calculated as 0.79 (95% Ci: 0.862–0.866; *I* ^2 ^= 97%) and 0.77 (95% Ci: 0.596–0.878; *I* ^2 ^= 97%) respectively, for CU and CI cohorts.

**TABLE 3:** Summary of pooled quantitative information showing the sensitivities, specificities, and F1 scores of plasma versus CSF p‐tau217 by reference standard (PET).

Plasma p‐tau217			
Reference standard	Sensitivity%	Specificity%	F1 score
Aβ PET positivity	82	86	0.78
Aβ PET in CU	76	83	0.65
Aβ PET in CU+CI	83	88	0.80
Aβ PET in CI	85	86	0.87
Tau PET positivity	83	83	0.75
Tau PET in CU	79	75	0.48
Tau PET in CU+CI	85	86	0.81
Tau PET in CI	81	85	0.83
CSF p‐Tau217			

Reference standard	Sensitivity %	Specificity (%)	F1 score
Aβ PET positivity	79	91	0.8
Aβ PET in CU	81	82	0.56
Aβ PET in CU+CI	79	92	0.84
Tau PET positivity	91	84	0.8
Tau PET in CU	85	84	0.33
Tau PET in CU+CI	91	86	0.83
Tau PET in CI	98	57	0.85

Abbreviations: Aβ; amyloid beta; CI, cognitively impaired; CSF, cerebrospinal fluid; CU, cognitively unimpaired; PET, positron emission tomography; p‐tau, phosphorylated tau.

### 3.2 Evaluation of plasma p‐tau217 for predicting tau PET positivity

To evaluate the accuracy of plasma p‐tau217 for diagnosis, 11 studies with tau PET imaging that met inclusion criteria were evaluated. The pooled sensitivity was 83% (95% Ci: 77.7%–87.2% *I* ^2 ^= 83%), the pooled specificity was 83% (95% Ci: 79.8%–86.2% *I* ^2 ^= 93%), and the F1 score was 0.75 (95% Ci: 0.67–0.809; *I* ^2 ^= 74%) for detecting tau deposition on PET. The pooled sensitivity of detecting tau PET positivity in CU participants was 79% (95% Ci: 69.7%–85.9%, *I* ^2 ^= 65%), with a pooled specificity of 75% (95% Ci: 67%–81.9%, *I* ^2 ^= 95%), and an F1 score of 0.48 (95% Ci: 0.345–0.605; *I* ^2 ^= 95%; Table 3). Among CIs, p‐tau217 demonstrated a pooled sensitivity of 81% (95% Ci: 67.2%–90.1%; *I* ^2 ^= 89%), a pooled specificity of 85% (95%  Ci: 76.6%–90.5% *I* ^2 ^= 81%), and an F1 score of 0.83 (95%  Ci: 0.788–0.868; *I* ^2 ^= 74%). In mixed CU+CI cohorts, the sensitivity and specificity were 83% (95%  Ci: 77.5%–90.5%; *I* ^2 ^= 85%) and 86% (95%  Ci: 83.3%–88.6%; *I* ^2 ^= 58%), respectively, with an F1 score of 0.81 (95%  Ci: 0.747–0.857; *I* ^2 ^= 83%; Table 3). When using different tau radiotracers as the reference standard, the best performance of p‐tau217 was observed in studies that used [18F]Flortaucipir with an F1 score of 0.78 (95%  Ci: 0.582–0.903; *I* ^2 ^= 98%). F1 scores of plasma p‐tau217 were 0.7 (95%  Ci: 0.626–0.782; *I* ^2 ^= 92%) and 0.71 (95%  Ci: 0.466–0.860; *I* ^2 ^= 97%) in studies that used [18F]RO948 and [18F]MK‐6240, respectively.

### 3.3 Evaluation of CSF p‐tau217 for predicting amyloid PET positivity

In five studies, amyloid PET imaging was used to evaluate the accuracy of CSF. The findings demonstrated a pooled sensitivity of 79% (95%  Ci: 73.2%–83.8% *I* ^2 ^= 53%) and a pooled specificity of 91% (95%  Ci: 85%–94.1% *I* ^2 ^= 81%), resulting in an F1 score of 0.8 (95%  Ci: 0.669–0.885; *I* ^2 ^= 93%). In mixed CU+CI cohorts, the DTA of p‐tau217 was shown to have a pooled sensitivity of 79% (95%  Ci: 72.1%–84.4% *I* ^2 ^= 63%), pooled specificity of 92% (95%  Ci: 89.4%–93.2% *I* ^2 ^= 11%), and an F1 score of 0.84 (95%  Ci: 0.784–0.877; *I* ^2 ^= 66%) in detecting Aβ deposition on PET (Table 3). [18F]Flutemetamol was the most commonly used Aβ PET radiotracer; CSF p‐tau217 demonstrated an F1 score of 0.73 (95%  Ci: 0.543–0.86; *I* ^2 ^= 95%) compared to this tracer.

### 3.4 Evaluation of CSF p‐tau217 for predicting tau PET positivity

Tau PET imaging was used in five studies to determine the DTA of CSF p‐tau217, showing a pooled sensitivity of 91% (95%  Ci: 86.5%–94.6% *I* ^2 ^= 71%), pooled specificity of 84% (95%  Ci: 77.1%–88.8% *I* ^2 ^= 91%), and F1 score of 0.8 (95%  Ci: 0.705–0.869; *I* ^2 ^= 92%). In identifying tau deposition among CU+CI studies samples, p‐tau217 had a pooled sensitivity of 91% (95%  Ci: 85.5%–94% *I* ^2 ^= 71%), pooled specificity of 86% (95%  Ci: 81.1%–90.2% *I* ^2 ^= 89%), and F1 score of 0.83 (95%  Ci: 0.772–0.87; *I* ^2 ^= 87%; Table 3). CSF p‐tau217 demonstrated the highest concordance with [18F]Flortaucipir with an F1 score of 0.88 (95%  Ci: 0.848–0.904; *I* ^2 ^= 0%).

### 3.5 Plasma p‐tau217 assays

In measuring plasma p‐tau217, the Meso Scale Discovery (MSD)‐based Lilly immunoassay had a pooled sensitivity of 86% (95%  Ci: 80.3%–89.5%; *I* ^2 ^= 90%) and a pooled specificity of 86% (95%  Ci: 83.1%–88.7%; *I* ^2 ^= 86%) and F1 score of 0.78 (95%  Ci: 0.714–0.834; *I* ^2 ^= 94%) in 14 studies. The Single Molecule Array (Simoa) ALZpath, based on six studies, had a pooled sensitivity of 85% (95%  Ci: 80%–89%; *I* ^2^ = 55%) and a pooled specificity of 84% (95%  Ci: 80.7%–86.2%; *I* ^2 ^= 40%) and F1 score of 0.78 (95%  Ci: 0.692–0.84; *I* ^2 ^= 89%). Simoa Jansson R&D, based on five studies, had a pooled sensitivity of 81% (95%  Ci: 77.2%–83.7%; *I* ^2 ^= 52%) and a pooled specificity of 84% (95%  Ci: 80.4%–87.1%; *I* ^2 ^= 74%) and F1 score of 0.77 (95%  Ci: 0.704–0.829; *I* ^2 ^= 92%). Three studies used mass spectrometry, which had a pooled sensitivity of 85% (95% Ci: 78.1%–89.2%; *I* ^2^ = 81%), a pooled specificity of 90% (95% Ci: 88%–91%; *I* ^2^ = 0%), and an F1 score of 0.83 (95% Ci: 0.75–0.89; *I* ^2^ = 93%). Note that grouping these three mass spectrometry studies provides an approximate estimate of the accuracy of mass spectrometry in measuring p‐tau217, although there are differences in these spectrometry methods: immunoprecipitation‐mass spectrometry,^52^ liquid chromatography‐high resolution mass spectrometry,^2^ and liquid chromatography‐tandem mass spectrometry.^27^

### 3.6 CSF p‐tau217 assays

The MSD‐based Lilly immunoassay in measuring plasma p‐tau217 had a pooled sensitivity of 91% (95%  Ci: 85%–94.6%; *I* ^2 ^= 78%) and a pooled specificity of 85% (95%  Ci: 80%–89%; *I* ^2 ^= 55%) and F1 score of 0.79 (95%  Ci: 0.699–0.862; *I* ^2 ^= 92%) in five studies.

### 3.7 Comparison of p‐tau217 assays by cognitive status

In these studies, the measurement and extraction of p‐tau217 were performed using MSD‐based Lilly immunoassay, Simoa ALZpath, Simoa Jansson R&D, Simoa Honebrew, and mass spectrometry for measuring CSF and plasma p‐tau217.

In plasma p‐tau217, MSD‐based Lilly, Simoa ALZpath, and Simoa Jansson R&D showed weaker diagnostic power than mass spectrometry in measuring plasma p‐tau 217 among CU individuals, with F1 scores of 0.63 (95%  Ci: 0.479–0.761; *I* ^2 ^= 92%), 0.54 (95%  Ci: 0.441–0.642; *I* ^2 ^= 18%), and 0.57 (95%  Ci: 0.492–0.647; *I* ^2 ^= 47%), respectively, compared to the F1 score of 0.78 for mass spectrometry. However, Lumipulse Fujirebio with an F1 score of 0.9—based on only one study—and Simoa‐based assays had an F1 score of 0.86 (95%  Ci: 0.81–0.892; *I* ^2 ^= 67%) and 0.89 (95%  Ci: 0.839–0.925; *I* ^2 ^= 57%) for ALZpath and Jansson R&D, respectively, in Cis; these were almost similar to that of mass spectrometry and better than MSD's F1 score of 0.83 (95%  Ci: 0.682–0.918; *I* ^2 ^= 86%; Table 4). However, given the limited data for CSF p‐tau217, MSD‐based‐Lilly had satisfactory results among mixed CU/CI cohorts, with F1 scores of 0.82 (95%  Ci: 0.765–0.858; *I* ^2 ^= 85%; Table 5).

**TABLE 4:** Summary statistics for all included studies that used plasma p‐tau217, stratified by PET reference standard or plasma biomarker assay.

	Sensitivity: 95% Ci lower bound	Sensitivity: 95% Ci upper bound	Specificity: 95% Ci lower bound	Specificity: 95% Ci upper bound	DOR: 95% Ci lower bound	DOR: 95% Ci upper bound	F1 score: 95% Ci lower bound	F1 score: 95% Ci upper bound
Data stratified based on imaging reference standard								
Amyloid PET imaging (11549^a^:23^b^)	0.785	0.843	0.840	0.878	22.97	36.51	0.738	0.816
Amyloid PET imaging: CU (4073^a^:9^b^)	0.714	0.803	0.786	0.858	10.09	22.37	0567	0.716
Amyloid PET imaging: CU+CI (4588^a^:13^b^)	0.777	0.873	0.855	0.906	29.31	50.18	0.752	0.841
Amyloid PET imaging: CI (2352^a^:5^b^)	0.814	0.882	0.828	0.882	25.93	51.47	0.847	0.894
Tau PET imaging (7343^a^:11^b^)	0.777	0.872	0.798	0.862	18.05	38.86	0.670	0.809
Tau PET imaging: CU (2773^a^:4^b^)	0.697	0.859	0.670	0.819	5.75	23.51	0.345	0.608
Tau PET imaging: CU+CI (3156^a^:8^b^)	0.775	0.905	0.833	0.886	25.02	58.19	0.747	0.857
Tau PET imaging: CI (1414^a^:3^b^)	0.672	0.901	0.766	0.905	13.45	52.11	0.788	0.868
Data stratified based on plasma assay used								
MSD based‐Lilly immunoassay: CU (4071^a^:6^b^)	0.698	0.850	0.785	0.889	10.59	51.10	0.479	0.761
MSD based‐Lilly immunoassay: CU+CI (4635^a^:10^b^)	0.801	0.916	0.843	0.907	32.55	77.10	0.765	0.874
MSD based‐Lilly immunoassay: CI (324^a^:2^b^)	0.860	0.869	0.640	0.868	21.25	98.38	0.682	0.918
Simoa HD‐X immunoassay‐ALZpath: CU (214^a^:2^b^)	0.502	0.790	0.708	0.834	3.39	14.03	0.441	0.642
Simoa HD‐X immunoassay‐ALZpath: CU+CI (772^a^:4^b^)	0.828	0.904	0.813	0.882	31.24	65.64	0.728	0.832
Simoa HD‐X immunoassay‐ALZpath: CI (1816^a^:3^b^)	0.782	0.925	0.783	0.886	19.38	51.18	0.839	0.924
Simoa HD‐X immunoassay‐Jansson: CU (1816^a^:3^b^)	0.683	0.841	0.727	0.824	7.77	21.43	0.492	0.647
Simoa HD‐X immunoassay‐Jansson: CU+CI (3017^a^:6^b^)	0.771	0.864	0.803	0.898	20.09	47.002	0.711	0.841
Simoa HD‐X immunoassay‐Jansson: CI (1794^a^:3^b^)	0.721	0.860	0.802	0.898	15.68	38.90	0.810	0.892
Lumipulse‐Fujirebio: CI (427^a^:1^b^)	0.834	0.914	0.792	0.908	24.43	77.91	0.861	0.922
Mass spectrometry: CU (1080^a^:1^b^)	0.743	0.833	0.866	0.912	21.66	43.52	0.746	0.810
Mass spectrometry: CU+CI (93^a^:1^b^)	0.528	0.918	0.810	0.960	8.33	105.94	0.572	0.850
Mass spectrometry: CI (1413^a^:1^b^)	0.853	0.903	0.878	0.920	47.80	93.61	0.855	0.905

*Note*: F1 score—A harmonic mean of precision and recall that is used to assess predictive performance of a classification task. Subgroup data were extracted from studies based on whether they used amyloid or tau PET imaging as the reference standard, whether cognitively impaired or unimpaired groups were included, and which p‐tau217 assay was used.

Abbreviations: CI, cognitively impaired; Ci, confidence interval; CU, cognitively unimpaired; DOR, diagnostic odds ratio; MSD, Meso Scale Discovery; PET, positron emission tomography; p‐tau, phosphorylated tau.

^a^ The total number of participants based on the datasets available,

^b^ The number of total studies.

**TABLE 5:** Summary statistics for all included studies that used CSF p‐tau217, stratified by PET reference standard or CSF biomarker assay.

	Sensitivity: 95% Ci lower bound	Sensitivity: 95% Ci upper bound	Specificity: 95% Ci lower bound	Specificity: 95% Ci upper bound	DOR: 95% Ci lower bound	DOR: 95% Ci upper bound	F1 score: 95% Ci lower bound	F1 score: 95% Ci upper bound
**Data stratified based on imaging reference standard**								
Amyloid PET imaging (2050^a^:5^b^)	0.732	0.838	0.850	0.941	21.86	74.36	0.669	0.885
Amyloid PET imaging: CU (658^a^:1^b^)	0.659	0.914	0.773	0.865	8.59	44.98	0.445	0.624
Amyloid PET imaging: CU+CI (1392^a^:4^b^)	0.721	0.844	0.894	0.933	24.12	104.28	0.784	0.877
Tau PET imaging (3288^a^:5^b^)	0.865	0.946	0.771	0.888	49.03	87.11	0.705	0.869
Tau PET imaging: CU (219^a^:1^b^)	0.546	0.981	0.735	0.849	4.58	100.60	0.222	0.460
Tau PET imaging: CU+CI (2888^a^:5^b^)	0.855	0.940	0.811	0.902	50.52	91.77	0.772	0.870
Tau PET imaging: CI (181^a^:1^b^)	0.931	0.998	0.453	0.681	15.23	287.48	0.795	0.891
**Data stratified based on CSF assay used**								
MSD based‐Lilly immunoassay: CU (219^a^:1^b^)	0.546	0.981	0.735	0.849	4.58	100.60	0.222	0.460
MSD based‐Lilly immunoassay: CU+CI (4106^a^:6^b^)	0.836	0.940	0.814	0.908	40.02	83.66	0.765	0.858
MSD based‐Lilly immunoassay: CI (181^a^:1^b^)	0.931	0.998	0.453	0.681	15.23	287.48	0.795	0.891
Simoa HD‐X immunoassay‐Homebrew: CU (329^a^:1^b^)	0.659	0.914	0.773	0.865	8.59	44.98	0.445	0.624
Simoa HD‐X immunoassay‐Jansson: CU+CI (344^a^:1^b^)	0.773	0.940	0.878	0.978	37.78	328.28	0.828	0.938

*Note*: F1 score—A harmonic mean of precision and recall that is used to assess predictive performance of a classification task. Subgroup data were extracted from studies based on whether they used amyloid or tau PET imaging as the reference standard, whether cognitively impaired or unimpaired groups were included, and which p‐tau217 assay was used.

Abbreviations: CI, cognitively impaired; Ci, confidence interval; CU, cognitively unimpaired; DOR, diagnostic odds ratio; MSD, Meso Scale Discovery; PET, positron emission tomography; p‐tau, phosphorylated tau.

^a^ The total number of participants based on the datasets available.

^b^ The number of total studies.

### 3.8 Quality assessment

To assess potential biases in the selected studies, we used QUADAS‐2, a tool that scrutinizes the validity of the research. Our findings revealed that none of the studies exhibited significantly high risk biases. Reflecting minimal publication bias, the data were symmetrically arranged around the actual effect in an inverted funnel shape (Figure S6 in supporting information). Among the 30 studies, the majority demonstrated low overall risk of bias across all domains, indicating that the design and conduct of these studies were robust and had minimal potential for bias. Most studies exhibited low risk in all QUADAS‐2 categories. However, a few studies were rated as having a high or moderate risk of bias due to high overall risk, primarily due to concerns regarding patient selection or the flow and timing of the study procedures. Two studies were evaluated as having moderate overall risk due to issues related to the index test and reference standard applicability. Despite these concerns, the overall quality of the evidence is robust, with most studies meeting the criteria for reliable diagnostic accuracy of p‐tau217 (Table S1).

## 4 DISCUSSION

Recent development of novel, sensitive, and specific fluid biomarkers for AD pathology provides more efficient and cost‐effective screening tools for AD. p‐tau species are linked to both amyloid plaque and neurofibrillary tau tangle formation^54^, ^55^ and have been investigated as potential early biomarkers of disease. For example, p‐tau181, p‐tau217, and p‐tau231 have all been reported to be associated with early amyloid changes, whereas p‐tau205 alterations occur at later stages of the disease.^11^, ^56^, ^57^, ^58^ Recently, elevated p‐tau217 has been shown to even be detected in CUs who are Aβ PET positive, before tau PET becomes positive.^38^, ^59^, ^60^ Therefore, p‐tau217 has been shown to be particularly useful in capturing early cerebral Aβ plaque deposition.^38^, ^61^, ^62^

Triana‐Baltzer et al. demonstrated that plasma and CSF p‐tau217 exhibit a moderate correlation, indicating that while they share some commonality, their levels do not perfectly align,^34^, ^35^, ^63^ which may be due to peripheral dilution and clearance effects, particularly in the setting of chronic renal insufficiency.^64^ The correlation between CSF and plasma p‐tau may also reflect differences in permeability across the blood–brain and blood–CSF barriers.^65^, ^66^, ^67^ Notably, these processes may also vary based on CSF or blood fluid turnover rates, time of day, and compartment volumes.^68^

Some studies have reported that p‐tau217 is more precise than other tau biomarkers in distinguishing between mild cognitive impairment and AD.^69^, ^70^ Our study has focused specifically on analyzing the utility of p‐tau217 in predicting underlying AD pathology, rather than cognitive status. We conducted a comprehensive search and separately analyzed all the data obtained from plasma and CSF to detect amyloid and tau PET positivity. This analysis was conducted in the form of a DTA meta‐analysis, which differs from prior studies that were focused on the effect size and ratio of means (ROM). Effect size meta‐analysis and ROM meta‐analysis are used to estimate the average effectiveness of an intervention across different studies; effect size meta‐analysis is used to quantify continuous outcomes, while ROM meta‐analysis focuses on quantifying relationships. On the other hand, DTA meta‐analysis aims to provide an overall summary of the accuracy of a test.^53^, ^71^, ^72^ Because it is crucial to manage FP and FN in medical testing; we focused on the F1 score (Figure S7 in supporting information), which weighs the FP and FN, along with pooled sensitivity and specificity.

Higher correlation with biomarkers and cognitive measures results in more accuracy for p‐tau217 compared to other p‐tau epitopes; it has demonstrated a 3.3‐ to 3.9‐fold increase in AD, while p‐tau181 only has shown a 1.7‐fold increase.^25^, ^56^, ^61^ In this study, we demonstrated that p‐tau217 closely reflects amyloid and tau burden on PET. Our pooled results demonstrated that CSF p‐tau217 had slightly increased sensitivity in predicting tau PET positivity compared to plasma p‐tau217. The best performance of plasma p‐tau217 was observed in CIs, which were slightly higher in amyloid PET than in tau PET (F1 score of 0.87 vs. 0.83). A study by Ossenkoppele et al. using CSF p‐tau217 among CIs indicated its strong performance (0.85); consistently, in CU individuals, CSF and plasma p‐tau217 outperformed p‐tau181 and Aβ 1‐42/Aβ 1‐40 in detecting amyloid positivity.^25^, ^34^, ^73^ While Jonaitis et al.^25^ reported high DTA for p‐tau217 among CU individuals, our pooled results indicated a weaker performance for p‐tau217 in predicting amyloid and tau PET positivity among CU individuals.

Studies have reported high DTA of p‐tau217 in detecting amyloid PET Centiloid (CL) values^18^, ^74^ and Braak stages on tau PET.^15^, ^18^ CL standardization of amyloid PET deposition aids in the harmonization of the quantification of Aβ deposition and provides a threshold for neuritic plaque density across tracers.^75^ Braak staging, on the other hand, traditionally used in neuropathology to classify the progression of neurofibrillary tangles, has been adapted for tau PET imaging by using the brain regions identified by Braak and Braak to assess the tau PET signal.^76^ The revised criteria from the Alzheimer's Association Workgroup recommend using tau PET imaging for in vivo staging, by characterizing medial temporal region uptake, moderate neocortical uptake, and high neocortical uptake, which have shown concordance with traditional Braak staging.^77^

Which reference PET tracer was used in each study may have affected the reported DTA in several ways.^76^ While the tracers demonstrated similar specific binding patterns and are largely concordant, there are variations in their dynamic range. These variations are primarily attributed to differences in tracer binding properties (B_max_, Kd) and clearance rates, rather than specificity.^78^, ^79^ For instance, MK‐6240 shows a greater dynamic range in standardized uptake volume ratio estimates compared to flortaucipir, which may be advantageous for detecting early tau pathology or small longitudinal changes.^80^, ^81^

The studied laboratory assays for p‐tau217 in this meta‐analysis included immunoassays that are conducted by Eli Lilly and Quanterix using the Simoa platform, while the mass spectrometry is performed using liquid chromatography‐mass spectrometry.^82^, ^83^ In the case of plasma p‐tau217, our results showed that the measurement of this biomarker using immunoassays and mass spectrometry was almost similar in CU+CI and CI‐only cohorts. However, in the case of CU individuals, mass spectrometry results indicated an F1 score of 0.78, while the F1 scores obtained from MSD‐based Lilly and Simoa HD‐X were lower.^27^

Evaluating the DTA of p‐tau217 using separate assays in a head‐to‐head comparison in the case of CI individuals, Janssen R&D results were slightly superior to those of ALZpath while Lumipulse‐Fujirebio provided more accurate results in predicting PET positivity.^30^, ^32^ Differences in assay characteristics may be responsible for the variations in accurately detecting p‐tau217: mass spectrometry may not directly measure the intact tau protein and targets shorter fragments, whereas immunoassays focus on longer, well‐defined tau peptides.^83^, ^84^

### 4.1 Limitations and future directions

Our criteria required studies to report the DTA of p‐tau217 for detecting amyloid‐PET positivity, which may have excluded studies reporting other outcomes. In addition, our study predominantly included studies with dementia‐based cohorts and high prevalence rates of PET‐positive cases, commonly found in clinical settings. Consequently, our results may not be generalizable to community‐based populations with lower dementia prevalence and more diverse comorbidities. Despite efforts to standardize data extraction, we encountered variability in reporting, with some studies not separating results based on different radiotracers and varying in their reporting of sensitivity and specificity. The included studies used various cut‐off points and laboratory techniques for p‐tau217 assays, reflecting the current lack of standardized methods across laboratories. Few studies in our review investigated CSF p‐tau217, likely due to the trend toward non‐invasive collection procedures. Additionally, it is essential to interpret the results with great care, especially when addressing heterogeneity as this is not a head‐to‐head comparison study of assays in a distinct cohort. Heterogeneity reflects variability in study outcomes, which can stem from differences in study design, populations, or methodologies. This variability can significantly influence the overall results and conclusions of the meta‐analysis, potentially leading to biased or misleading interpretations.^85^, ^86^ For instance, using different assays on the same cohort could affect the results, thereby impacting the interpretation of the findings.

### 4.2 Conclusion

Our data demonstrate that p‐tau217 reliably identifies AD pathology in vivo; plasma and CSF p‐tau 217 can detect amyloid and tau PET imaging positivity, with the highest performance among CI individuals. Future studies in larger community‐based settings may provide additional information on the utility of p‐tau217 in screening for preclinical AD.

## AUTHOR CONTRIBUTIONS

Conceptualization: The study was conceptualized and designed by Mohammad Khalafi and Gloria C. Chiang. Methodology: Mohammad Khalafi and Gloria C. Chiang developed the experimental procedures and research methodologies, with additional support from the research team. Data collection: Mohammad Khalafi, Kiarash Shirbandi, and William J. Dartora were responsible for conducting the experiments, performing clinical assessments, and gathering critical data essential to the research design. Data analysis: The database search was conducted by Mohammad Khalafi, Kiarash Shirbandi, and William J. Dartora. Data analysis was performed under the supervision of the statistical team including Samprit Banerjee, Kiarash Shirbandi, William J. Dartora, Kewei Chen, and Ke Xi. Writing: Mohammad Khalafi drafted the manuscript under the guidance and supervision of Gloria C. Chiang and the research received critical revisions from Mony J. de Leon, Laura Beth J. McIntire, Tracy A. Butler, Silky Pahlajani, Lidia Glodzik, Qolamreza R. Razlighi, Seyed Hani Hojjati, Liangdong Zhou, Kewei Chen, Nancy Foldi, Krista M. Wartchow, and Yi Li. Supervision: Overall guidance and oversight were provided by Gloria C. Chiang.

## CONFLICT OF INTEREST STATEMENT

G.C. receives consulting fees from Life Molecular Imaging and speaker honoraria from Efficient CME and PeerView. K.C. receives consulting fees from Shanghai Green Valley Pharmaceutical, ADMdx, and Banner Alzheimer's Institute. M.K., K.S., L.M., Y.L., L.Z., T.B., W.D., K.W., N.F., L.G., M.d.L., S.H., Q.R., K.X., S.B., and S.P. report no competing interests. Author disclosures are available in the supporting information.

## ETHICS APPROVAL

Because this is a meta‐analysis of previously published studies, ethics approval was considered to be not required.

## STATEMENT OF DIVERSITY, EQUITY, AND INCLUSION

Our meta‐analysis of 30 studies on p‐tau217 biomarkers spans diverse global regions, including North America, Europe, Asia, and Oceania. By including cohorts from these regions, we aimed to demonstrate the representation of different ethnicities and health‐care systems, reflecting our commitment to equity and inclusion in research and enhancing the generalizability of our findings. By conducting this study, we aimed to better understand the utility of p‐tau217 assays in AD across different populations.

## Supporting information



Supporting Information

Supporting Information

Supporting Information

Supporting Information

Supporting Information

Supporting Information

Supporting Information

Supporting Information

Supporting Information

Supporting Information
