# Supplementary material for: Diagnostic accuracy of phosphorylated tau217 in detecting Alzheimer's disease pathology among cognitively impaired and unimpaired: A systematic review and meta‐analysis
Source: Alzheimers Dement. 2024 Dec 23;21(2):e14458. doi: 10.1002/alz.14458 (PMC11848338; doi:10.1002/alz.14458)

Supplemental Figure-2 Forest plots showing the diagnostic performance metrics of cerebrospinal fluid (CSF) and plasma phosphorylated tau-217 (p-Tau217), stratified by the reference standard of either amyloid and/or tau PET. g: group of studies that used CSF or plasma p-Tau217 in predicting PET positivity; I^2: the proportion of variation due to heterogeneity; τ^2: between-study variance in a random-effects model. χ^2_x: chi-square test statistic with x degrees of freedom (df = x).

Panel (A) shows the sensitivity of each study, illustrating the ability of p-Tau217 to correctly identify true positives (either amyloid and/or tau PET positive). Events: number of true positive cases identified by the test; Total: actual total number of positive subjects in the cohort; Proportion: proportion of positive cases— also known as the true positive rate, reflecting the assay's ability to correctly identify positive cases, or its effectiveness in detecting the condition when present (sensitivity). Panel (B) shows the specificity of each study, illustrating the ability of p-Tau217 to correctly identify true negatives. Events: indicate the number of true negative cases detected by the test; Total: actual total number of negative subjects in the cohort; Proportion: proportion of negative cases, detected by the assay (specificity). Panel (C) shows the diagnostic odds ratio (OR), a comprehensive measure of test effectiveness, which combines sensitivity and specificity into a single metric. Events: number of true positive cases identified by the test; Total: actual number of positive cases in the cohort; Proportion: expressed as the DOR, provides the odds of achieving a true positive result versus a false positive. Panel (D) highlights the F1 score, representing the harmonic mean of sensitivity and precision, providing a balanced evaluation of the test's predictive power in distinguishing PET-positive cases. Events: number of cases where precision and recall both resulted in a truly positive outcome; Total: total number of positive predictions made by the test; Proportion: balance between sensitivity/recall and precision, giving a single measure that accounts for both false positives and false negatives.

Supp, Figure-2A

Study	Events	Total	Proportion	95%-CI
**g = CSF, Aß PET+**				
Suarez-Calevt, 2020b	34	42	0.810	[0.659; 0.914]
Leuzy, 2021c	207	284	0.729	[0.673; 0.780]
Mendes, 2024b	43	56	0.768	[0.636; 0.870]
Palmqvist, 2020b	131	162	0.809	[0.740; 0.866]
Therriault, 2022a	62	71	0.873	[0.773; 0.940]
Random effects model		615	0.790	[0.732; 0.838]
Heterogeneity: I^2 = 53%, τ^2 = 0.0637, p = 0.07				
**g = CSF, Tau PET+**				
Ossenkoppele, 2021c	11	13	0.846	[0.546; 0.981]
Ossenkoppele, 2021b	100	102	0.980	[0.931; 0.998]
Janelidze, 2020a	63	72	0.875	[0.776; 0.941]
Janelidze, 2020b	62	78	0.795	[0.688; 0.878]
Janelidze, 2020c	63	72	0.875	[0.776; 0.941]
Leuzy, 2021a	108	119	0.908	[0.841; 0.953]
Leuzy, 2021b	177	203	0.872	[0.818; 0.915]
Mendes, 2024a	42	42	1.000	[0.916; 1.000]
Ossenkoppele, 2021a	108	112	0.964	[0.911; 0.990]
Palmqvist, 2020a	160	167	0.958	[0.916; 0.983]
Random effects model		980	0.914	[0.865; 0.946]
Heterogeneity: I^2 = 71%, τ^2 = 0.4235, p < 0.01				
**g = Plasma, Aß PET+**				
Barthelemy, 2024a	311	349	0.891	[0.854; 0.922]
Dore, 2022m	92	126	0.730	[0.644; 0.805]
Dore, 2022n	106	126	0.841	[0.766; 0.900]
Dore, 2022o	103	126	0.817	[0.739; 0.881]
Figdore, 2024a	240	273	0.879	[0.834; 0.915]
Figdore, 2024b	230	274	0.839	[0.790; 0.881]
Niimi, 2024a	35	39	0.897	[0.758; 0.971]
Therriault, 2024b	75	88	0.852	[0.761; 0.919]
Therriault, 2024e	80	88	0.909	[0.829; 0.960]
Clifford, 2023c	77	96	0.802	[0.708; 0.876]
Clifford, 2023d	133	204	0.652	[0.582; 0.717]
Dore, 2022p	35	47	0.745	[0.597; 0.861]
Dore, 2022q	39	47	0.830	[0.692; 0.924]
Dore, 2022r	41	47	0.872	[0.743; 0.952]
Jonaitis, 2023b	59	69	0.855	[0.750; 0.928]
Meyer, 2024	9	16	0.562	[0.299; 0.802]
Mila-aloma, 2022	19	27	0.704	[0.498; 0.862]
Niimi, 2024c	32	42	0.762	[0.605; 0.879]
Rissman, 2024	268	339	0.791	[0.743; 0.833]
Therriault, 2024c	24	32	0.750	[0.566; 0.885]
Therriault, 2024f	23	32	0.719	[0.533; 0.863]
Thijssen, 2021e	8	11	0.727	[0.390; 0.940]
Ashton, 2024 b	44	52	0.846	[0.719; 0.931]
Ashton, 2024d	49	52	0.942	[0.841; 0.988]
Asken, 2024	80	90	0.889	[0.805; 0.945]
Brum, 2023	200	211	0.948	[0.909; 0.974]
Dore, 2022j	128	171	0.749	[0.677; 0.812]
Dore, 2022k	138	171	0.807	[0.740; 0.863]
Dore, 2022l	140	171	0.819	[0.753; 0.873]
Gonzales-ortiz, 2023	16	21	0.762	[0.528; 0.918]
Meilke, 2022c	107	211	0.507	[0.438; 0.576]
Mendes, 2024c	52	56	0.929	[0.827; 0.980]
Niimi, 2024b	68	81	0.840	[0.741; 0.912]
Palmqvist, 2020d	92	162	0.568	[0.488; 0.645]
Thanapornsangsuth, 2024	31	34	0.912	[0.763; 0.981]
Therriault, 2022c	56	71	0.789	[0.676; 0.877]
Therriault, 2024a	98	121	0.810	[0.729; 0.876]
Therriault, 2024d	99	121	0.818	[0.738; 0.882]
Thijssen, 2021a	71	94	0.755	[0.656; 0.838]
Thijssen, 2021b	28	29	0.966	[0.822; 0.999]
VandeVrede, 2023a	76	92	0.826	[0.733; 0.897]
Woo, 2023b	40	44	0.909	[0.783; 0.975]
Xiao, 2023	9	10	0.900	[0.555; 0.997]
Random effects model		4563	0.816	[0.785; 0.843]
Heterogeneity: I^2 = 85%, τ^2 = 0.2991, p < 0.01				
**g = Plasma, Tau PET+**				
Barthelemy, 2024b	266	306	0.869	[0.826; 0.905]
Dore, 2022g	81	112	0.723	[0.631; 0.804]
Dore, 2022h	81	112	0.723	[0.631; 0.804]
Dore, 2022i	77	112	0.688	[0.593; 0.772]
Ossenkoppele, 2021f	100	105	0.952	[0.892; 0.984]
Clifford, 2023a	65	87	0.747	[0.643; 0.834]
Clifford, 2023b	95	112	0.848	[0.768; 0.909]
Dore, 2022d	24	31	0.774	[0.589; 0.904]
Dore, 2022e	27	32	0.844	[0.672; 0.947]
Dore, 2022f	19	32	0.594	[0.406; 0.763]
Jonaitis, 2023a	46	47	0.979	[0.887; 0.999]
Ossenkoppele, 2021e	11	15	0.733	[0.449; 0.922]
Ashton, 2024 a	46	51	0.902	[0.786; 0.967]
Ashton, 2024 c	46	52	0.885	[0.766; 0.956]
Dore, 2022a	101	142	0.711	[0.629; 0.784]
Dore, 2022b	111	142	0.782	[0.705; 0.847]
Dore, 2022c	99	143	0.692	[0.610; 0.767]
M-Carlgren, 2024a	85	93	0.914	[0.838; 0.962]
M-Carlgren, 2024b	49	51	0.961	[0.865; 0.995]
Meilke, 2022a	10	26	0.385	[0.202; 0.594]
Meilke, 2022b	20	31	0.645	[0.454; 0.808]
Mendes, 2024d	40	42	0.952	[0.838; 0.994]
Ossenkoppele, 2021d	100	108	0.926	[0.859; 0.967]
Palmqvist, 2020c	150	167	0.898	[0.842; 0.940]
Thijssen, 2021c	67	73	0.918	[0.830; 0.969]
Thijssen, 2021d	22	23	0.957	[0.781; 0.999]
VandeVrede, 2023b	62	73	0.849	[0.746; 0.922]
Random effects model		2320	0.830	[0.777; 0.872]
Heterogeneity: I^2 = 83%, τ^2 = 0.6196, p < 0.01				
**Random effects model**		**8478**	**0.832**	**[0.808; 0.853]**
Heterogeneity: I^2 = 84%, τ^2 = 0.4154, p < 0.01				
Test for subgroup differences: χ^2_3 = 12.44, df = 3 (p < 0.01)				

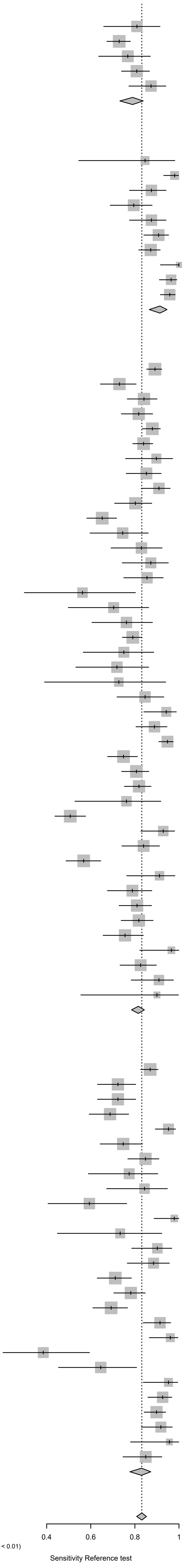

Supp, Figure-2B

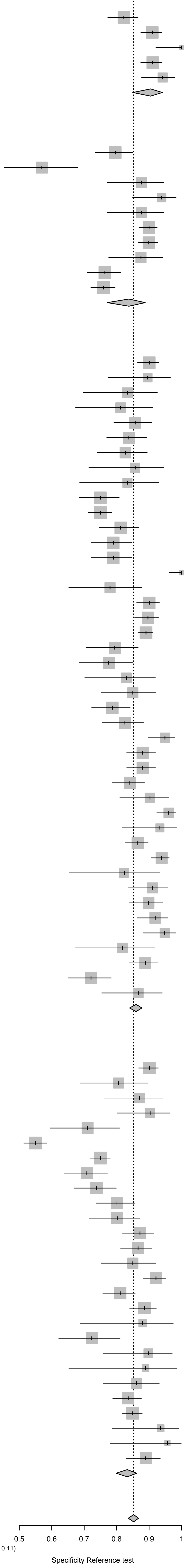

Study	Events	Total	Proportion	95%-CI
**g = CSF, Aß PET+**				
Suarez-Calevt, 2020b	236	287	0.822	[0.773; 0.865]
Leuzy, 2021c	314	345	0.910	[0.875; 0.938]
Mendes, 2024b	45	45	1.000	[0.921; 1.000]
Palmqvist, 2020b	297	326	0.911	[0.875; 0.940]
Therriault, 2022a	97	103	0.942	[0.878; 0.978]
Random effects model		1106	0.905	[0.850; 0.941]
Heterogeneity: I^2 = 81%, τ^2 = 0.2305, p < 0.01				
**g = CSF, Tau PET+**				
Ossenkoppele, 2021c	164	206	0.796	[0.735; 0.849]
Ossenkoppele, 2021b	45	79	0.570	[0.453; 0.681]
Janelidze, 2020a	57	65	0.877	[0.772; 0.945]
Janelidze, 2020b	61	65	0.938	[0.850; 0.983]
Janelidze, 2020c	57	65	0.877	[0.772; 0.945]
Leuzy, 2021a	459	510	0.900	[0.871; 0.925]
Leuzy, 2021b	383	426	0.899	[0.866; 0.926]
Mendes, 2024a	63	72	0.875	[0.776; 0.941]
Ossenkoppele, 2021a	220	288	0.764	[0.711; 0.812]
Palmqvist, 2020a	404	532	0.759	[0.721; 0.795]
Random effects model		2308	0.838	[0.771; 0.888]
Heterogeneity: I^2 = 91%, τ^2 = 0.4059, p < 0.01				
**g = Plasma, Aß PET+**				
Barthelemy, 2024a	318	353	0.901	[0.865; 0.930]
Dore, 2022m	43	48	0.896	[0.773; 0.965]
Dore, 2022n	40	48	0.833	[0.698; 0.925]
Dore, 2022o	39	48	0.812	[0.674; 0.911]
Figdore, 2024a	132	154	0.857	[0.792; 0.908]
Figdore, 2024b	129	154	0.838	[0.770; 0.892]
Niimi, 2024a	86	104	0.827	[0.740; 0.894]
Therriault, 2024b	36	42	0.857	[0.715; 0.946]
Therriault, 2024e	35	42	0.833	[0.686; 0.930]
Clifford, 2023c	153	204	0.750	[0.685; 0.808]
Clifford, 2023d	423	564	0.750	[0.712; 0.785]
Dore, 2022p	143	176	0.812	[0.747; 0.867]
Dore, 2022q	139	176	0.790	[0.722; 0.847]
Dore, 2022r	139	176	0.790	[0.722; 0.847]
Jonaitis, 2023b	95	95	1.000	[0.962; 1.000]
Meyer, 2024	46	59	0.780	[0.653; 0.877]
Mila-aloma, 2022	281	312	0.901	[0.862; 0.931]
Niimi, 2024c	260	290	0.897	[0.856; 0.929]
Rissman, 2024	659	740	0.891	[0.866; 0.912]
Therriault, 2024c	85	107	0.794	[0.705; 0.866]
Therriault, 2024f	83	107	0.776	[0.685; 0.851]
Thijssen, 2021e	44	53	0.830	[0.702; 0.919]
Ashton, 2024 b	68	80	0.850	[0.753; 0.920]
Ashton, 2024d	155	197	0.787	[0.723; 0.842]
Asken, 2024	123	149	0.826	[0.755; 0.883]
Brum, 2023	130	137	0.949	[0.898; 0.979]
Dore, 2022j	199	226	0.881	[0.831; 0.920]
Dore, 2022k	199	226	0.881	[0.831; 0.920]
Dore, 2022l	190	226	0.841	[0.786; 0.886]
Gonzales-ortiz, 2023	65	72	0.903	[0.810; 0.960]
Meilke, 2022c	195	203	0.961	[0.924; 0.983]
Mendes, 2024c	42	45	0.933	[0.817; 0.986]
Niimi, 2024b	340	393	0.865	[0.827; 0.897]
Palmqvist, 2020d	306	326	0.939	[0.907; 0.962]
Thanapornsangsuth, 2024	28	34	0.824	[0.655; 0.932]
Therriault, 2022c	91	100	0.910	[0.836; 0.958]
Therriault, 2024a	133	148	0.899	[0.838; 0.942]
Therriault, 2024d	136	148	0.919	[0.863; 0.957]
Thijssen, 2021a	91	96	0.948	[0.883; 0.983]
Thijssen, 2021b	36	44	0.818	[0.673; 0.918]
VandeVrede, 2023a	191	215	0.888	[0.838; 0.927]
Woo, 2023b	137	190	0.721	[0.652; 0.784]
Xiao, 2023	52	60	0.867	[0.754; 0.941]
Random effects model		7367	0.860	[0.840; 0.878]
Heterogeneity: I^2 = 81%, τ^2 = 0.1941, p < 0.01				
**g = Plasma, Tau PET+**				
Barthelemy, 2024b	365	405	0.901	[0.868; 0.928]
Dore, 2022g	50	62	0.806	[0.686; 0.896]
Dore, 2022h	54	62	0.871	[0.761; 0.943]
Dore, 2022i	56	62	0.903	[0.801; 0.964]
Ossenkoppele, 2021f	54	76	0.711	[0.595; 0.809]
Clifford, 2023a	427	777	0.550	[0.514; 0.585]
Clifford, 2023b	564	752	0.750	[0.717; 0.781]
Dore, 2022d	136	192	0.708	[0.639; 0.772]
Dore, 2022e	141	191	0.738	[0.670; 0.799]
Dore, 2022f	153	191	0.801	[0.737; 0.855]
Jonaitis, 2023a	89	111	0.802	[0.715; 0.871]
Ossenkoppele, 2021e	177	203	0.872	[0.818; 0.915]
Ashton, 2024 a	181	209	0.866	[0.812; 0.909]
Ashton, 2024 c	68	80	0.850	[0.753; 0.920]
Dore, 2022a	234	254	0.921	[0.881; 0.951]
Dore, 2022b	206	254	0.811	[0.757; 0.857]
Dore, 2022c	225	254	0.886	[0.840; 0.922]
M-Carlgren, 2024a	22	25	0.880	[0.688; 0.975]
M-Carlgren, 2024b	68	94	0.723	[0.622; 0.811]
Meilke, 2022a	35	39	0.897	[0.758; 0.971]
Meilke, 2022b	16	18	0.889	[0.653; 0.986]
Mendes, 2024d	62	72	0.861	[0.759; 0.931]
Ossenkoppele, 2021d	244	292	0.836	[0.788; 0.876]
Palmqvist, 2020c	452	532	0.850	[0.816; 0.879]
Thijssen, 2021c	29	31	0.935	[0.786; 0.992]
Thijssen, 2021d	22	23	0.957	[0.781; 0.999]
VandeVrede, 2023b	137	154	0.890	[0.829; 0.934]
Random effects model		5415	0.833	[0.798; 0.862]
Heterogeneity: I^2 = 93%, τ^2 = 0.2768, p < 0.01				
**Random effects model**		**16196**	**0.852**	**[0.836; 0.867]**

Heterogeneity: I^2 = 90%, τ^2 = 0.2622, p < 0.01

Test for subgroup differences: χ^2_3 = 5.98, df = 3 (p = 0.11)

Supp, Figure-2C

Study	Experimental Events	Experimental Total	Control Events	Control Total	OR	95%-CI
**g = CSF, Aß PET+**						
Suarez-Calevt, 2020b	34	85	8	244	19.66667	[8.59705; 44.98960]
Leuzy, 2021c	207	238	77	391	27.23000	[17.32227; 42.80458]
Mendes, 2024b	43	43	13	58	293.22222	[16.90866; 5084.92519]
Palmqvist, 2020b	131	160	31	328	43.27809	[25.05733; 74.74830]
Therriault, 2022a	62	68	9	106	111.37037	[37.78181; 328.28919]
**Random effects model**		**594**		**1127**	**40.32383**	**[21.86520; 74.36526]**
Heterogeneity: I^2 = 61%, τ^2 = 0.2741, p = 0.04						
**g = CSF, Tau PET+**						
Ossenkoppele, 2021c	11	53	2	166	21.47619	[4.58437; 100.60858]
Ossenkoppele, 2021b	100	134	2	47	66.17647	[15.23330; 287.48364]
Janelidze, 2020a	63	71	9	66	49.87500	[18.02903; 137.97278]
Janelidze, 2020b	62	66	16	77	59.09375	[18.68777; 186.86396]
Janelidze, 2020c	63	71	9	66	49.87500	[18.02903; 137.97278]
Leuzy, 2021a	108	159	11	470	88.36364	[44.56734; 175.19853]
Leuzy, 2021b	177	220	26	409	60.63596	[36.10429; 101.83608]
Mendes, 2024a	42	51	0	63	568.15789	[32.20685; 10022.81863]
Ossenkoppele, 2021a	108	176	4	224	87.35294	[31.05014; 245.74886]
Palmqvist, 2020a	160	288	7	411	72.14286	[32.98809; 157.77185]
**Random effects model**		**1289**		**1999**	**65.36090**	**[49.03800; 87.11708]**
Heterogeneity: I^2 = 0%, τ^2 = < 0.0001, p = 0.75						
**g = Plasma, Aß PET+**						
Barthelemy, 2024a	311	346	38	356	74.35940	[45.78032; 120.77940]
Dore, 2022m	92	97	34	77	23.27059	[8.50816; 63.64717]
Dore, 2022n	106	114	20	60	26.50000	[10.80693; 64.98145]
Dore, 2022o	103	112	23	62	19.40580	[8.25963; 45.59342]
Figdore, 2024a	240	262	33	165	43.63636	[24.43767; 77.91792]
Figdore, 2024b	230	255	44	173	26.97273	[15.77912; 46.10702]
Niimi, 2024a	35	53	4	90	41.80556	[13.20443; 132.35745]
Therriault, 2024b	75	81	13	49	34.61538	[12.16439; 98.50264]
Therriault, 2024e	80	87	8	43	50.00000	[16.82135; 148.62068]
Clifford, 2023c	77	128	19	172	12.15789	[6.71444; 22.01441]
Clifford, 2023d	133	274	71	494	5.61972	[3.97833; 7.93831]
Dore, 2022p	35	68	12	155	12.63889	[5.92815; 26.94626]
Dore, 2022q	39	76	8	147	18.31419	[7.88512; 42.53702]
Dore, 2022r	41	78	6	145	25.67117	[10.12595; 65.08120]
Jonaitis, 2023b	59	59	10	105	1082.33333	[62.26696; 18813.27541]
Meyer, 2024	9	22	7	53	4.54945	[1.42067; 14.56885]
Mila-aloma, 2022	19	50	8	289	21.52823	[8.70461; 53.24360]
Niimi, 2024c	32	62	10	270	27.73333	[12.40702; 61.99213]
Rissman, 2024	268	349	71	730	30.70979	[21.66581; 43.52900]
Therriault, 2024c	24	46	8	93	11.59091	[4.58520; 29.30063]
Therriault, 2024f	23	47	9	92	8.83796	[3.61294; 21.61940]
Thijssen, 2021e	8	17	3	47	13.03704	[2.88507; 58.91168]
Ashton, 2024 b	44	56	8	76	31.16667	[11.79529; 82.35162]
Ashton, 2024d	49	91	3	158	60.27778	[17.89308; 203.06232]
Asken, 2024	80	106	10	133	37.84615	[17.31845; 82.70552]
Brum, 2023	200	207	11	141	337.66234	[127.61658; 893.42510]
Dore, 2022j	128	155	43	242	21.93971	[12.91348; 37.27505]
Dore, 2022k	138	165	33	232	30.82155	[17.72897; 53.58280]
Dore, 2022l	140	176	31	221	23.83513	[14.06424; 40.39416]
Gonzales-ortiz, 2023	16	23	5	70	29.71429	[8.33368; 105.94827]
Meilke, 2022c	107	115	104	299	25.07812	[11.76599; 53.45174]
Mendes, 2024c	52	55	4	46	182.00000	[38.58321; 858.50806]
Niimi, 2024b	68	121	13	353	33.55588	[17.34120; 64.93190]
Palmqvist, 2020d	92	112	70	376	20.10857	[11.61474; 34.81393]
Thanapornsangsuth, 2024	31	37	3	31	48.22222	[11.00901; 211.22545]
Therriault, 2022c	56	65	15	106	37.74815	[15.48725; 92.00618]
Therriault, 2024a	98	113	23	156	37.77971	[18.74480; 76.14413]
Therriault, 2024d	99	111	22	158	51.00000	[24.10223; 107.91531]
Thijssen, 2021a	71	76	23	114	56.18261	[20.34663; 155.13558]
Thijssen, 2021b	28	36	1	37	126.00000	[14.87332; 1067.41500]
VandeVrede, 2023a	76	100	16	207	37.80208	[19.03365; 75.07743]
Woo, 2023b	40	93	4	141	25.84906	[8.81691; 75.78322]
Xiao, 2023	9	17	1	53	58.50000	[6.50807; 525.84705]
**Random effects model**		**4713**		**7217**	**28.96202**	**[22.97003; 36.51709]**
Heterogeneity: I^2 = 77%, τ^2 = 0.3844, p < 0.01						
**g = Plasma, Tau PET+**						
Barthelemy, 2024b	266	306	40	405	60.68125	[38.08271; 96.68993]
Dore, 2022g	81	93	31	81	10.88710	[5.12292; 23.13696]
Dore, 2022h	81	89	31	85	17.63710	[7.53763; 41.26856]
Dore, 2022i	77	83	35	91	20.53333	[8.08592; 52.14224]
Ossenkoppele, 2021f	100	122	5	59	49.09091	[17.59792; 136.94327]
Clifford, 2023a	65	415	22	449	3.60455	[2.17826; 5.96474]
Clifford, 2023b	95	283	17	581	16.76471	[9.75112; 28.82289]
Dore, 2022d	24	80	7	143	8.32653	[3.39345; 20.43085]
Dore, 2022e	27	77	5	146	15.22800	[5.56135; 41.69704]
Dore, 2022f	19	57	13	166	5.88462	[2.67108; 12.96431]
Jonaitis, 2023a	46	68	1	90	186.09091	[24.30938; 1424.54573]
Ossenkoppele, 2021e	11	37	4	181	18.72115	[5.54833; 63.16889]
Ashton, 2024 a	46	74	5	186	59.47143	[21.76708; 162.48621]
Ashton, 2024 c	46	58	6	74	43.44444	[15.21831; 124.02295]
Dore, 2022a	101	121	41	275	28.82195	[16.08449; 51.64633]
Dore, 2022b	111	159	31	237	15.36694	[9.25409; 25.51767]
Dore, 2022c	99	128	44	269	17.45690	[10.32652; 29.51075]
M-Carlgren, 2024a	85	88	8	30	77.91667	[19.07439; 318.28049]
M-Carlgren, 2024b	49	75	2	70	64.07692	[14.52266; 282.72044]
Meilke, 2022a	10	14	16	51	5.46875	[1.48791; 20.10011]
Meilke, 2022b	20	22	11	27	14.54545	[2.81079; 75.27065]
Mendes, 2024d	40	50	2	64	124.00000	[25.81428; 595.63924]
Ossenkoppele, 2021d	100	148	8	252	63.54167	[29.01688; 139.14466]
Palmqvist, 2020c	150	230	17	469	49.85294	[28.61739; 86.84635]
Thijssen, 2021c	67	69	6	35	161.91667	[30.83149; 850.33203]
Thijssen, 2021d	22	23	1	23	484.00000	[28.44526; 8235.32532]
VandeVrede, 2023b	62	79	11	148	45.42246	[20.09387; 102.67810]
**Random effects model**		**3048**		**4687**	**26.43123**	**[18.05778; 38.68748]**
Heterogeneity: I^2 = 82%, τ^2 = 0.7404, p < 0.01						
**Random effects model**		**9644**		**15030**	**31.29452**	**[26.01787; 37.64131]**
Heterogeneity: I^2 = 79%, τ^2 = 0.5050, p < 0.01						
Test for subgroup differences: χ^2_3 = 22.42, df = 3 (p < 0.01)						

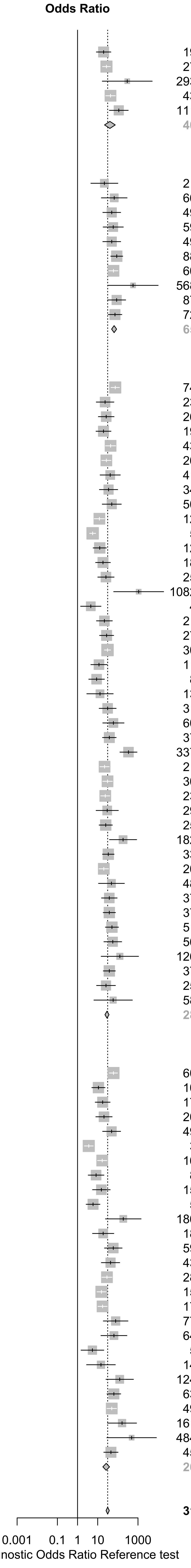

Supp, Figure-2D

Study	Events	Total	Proportion	95%-CI
**g = CSF, Aß PET+**				
Suarez-Calevt, 2020b	68	127	0.535	[0.445; 0.624]
Leuzy, 2021c	414	522	0.793	[0.756; 0.827]
Mendes, 2024b	86	99	0.869	[0.786; 0.928]
Palmqvist, 2020b	262	322	0.814	[0.767; 0.855]
Therriault, 2022a	124	139	0.892	[0.828; 0.938]
Random effects model		1209	0.798	[0.669; 0.885]
Heterogeneity: I^2 = 93%, τ^2 = 0.5354, p < 0.01				
**g = CSF, Tau PET+**				
Ossenkoppele, 2021c	22	66	0.333	[0.222; 0.460]
Ossenkoppele, 2021b	200	236	0.847	[0.795; 0.891]
Janelidze, 2020a	126	143	0.881	[0.816; 0.929]
Janelidze, 2020b	124	144	0.861	[0.794; 0.913]
Janelidze, 2020c	126	143	0.881	[0.816; 0.929]
Leuzy, 2021a	216	278	0.777	[0.723; 0.825]
Leuzy, 2021b	354	423	0.837	[0.798; 0.871]
Mendes, 2024a	84	93	0.903	[0.824; 0.955]
Ossenkoppele, 2021a	216	288	0.750	[0.696; 0.799]
Palmqvist, 2020a	320	455	0.703	[0.659; 0.745]
Random effects model		2269	0.800	[0.705; 0.869]
Heterogeneity: I^2 = 92%, τ^2 = 0.6362, p < 0.01				
**g = Plasma, Aß PET+**				
Barthelemy, 2024a	622	695	0.895	[0.870; 0.917]
Dore, 2022m	184	223	0.825	[0.769; 0.873]
Dore, 2022n	212	240	0.883	[0.836; 0.921]
Dore, 2022o	206	238	0.866	[0.816; 0.906]
Figdore, 2024a	480	535	0.897	[0.868; 0.922]
Figdore, 2024b	460	529	0.870	[0.838; 0.897]
Niimi, 2024a	70	92	0.761	[0.661; 0.844]
Therriault, 2024b	150	169	0.888	[0.830; 0.931]
Therriault, 2024e	160	175	0.914	[0.863; 0.951]
Clifford, 2023c	154	224	0.688	[0.622; 0.748]
Clifford, 2023d	266	478	0.556	[0.511; 0.602]
Dore, 2022p	70	115	0.609	[0.513; 0.698]
Dore, 2022q	78	123	0.634	[0.543; 0.719]
Dore, 2022r	82	125	0.656	[0.566; 0.739]
Jonaitis, 2023b	118	128	0.922	[0.861; 0.962]
Meyer, 2024	18	38	0.474	[0.310; 0.642]
Mila-aloma, 2022	38	77	0.494	[0.378; 0.610]
Niimi, 2024c	64	104	0.615	[0.515; 0.709]
Rissman, 2024	536	688	0.779	[0.746; 0.810]
Therriault, 2024c	48	78	0.615	[0.498; 0.723]
Therriault, 2024f	46	79	0.582	[0.466; 0.692]
Thijssen, 2021e	16	28	0.571	[0.372; 0.755]
Ashton, 2024 b	88	108	0.815	[0.729; 0.883]
Ashton, 2024d	98	143	0.685	[0.602; 0.760]
Asken, 2024	160	196	0.816	[0.755; 0.868]
Brum, 2023	400	418	0.957	[0.933; 0.974]
Dore, 2022j	256	326	0.785	[0.737; 0.829]
Dore, 2022k	276	336	0.821	[0.776; 0.861]
Dore, 2022l	280	347	0.807	[0.761; 0.847]
Gonzales-ortiz, 2023	32	44	0.727	[0.572; 0.850]
Meilke, 2022c	214	326	0.656	[0.602; 0.708]
Mendes, 2024c	104	111	0.937	[0.874; 0.974]
Niimi, 2024b	136	202	0.673	[0.604; 0.737]
Palmqvist, 2020d	184	274	0.672	[0.612; 0.727]
Thanapornsangsuth, 2024	62	71	0.873	[0.773; 0.940]
Therriault, 2022c	112	136	0.824	[0.749; 0.884]
Therriault, 2024a	196	234	0.838	[0.784; 0.882]
Therriault, 2024d	198	232	0.853	[0.801; 0.896]
Thijssen, 2021a	142	170	0.835	[0.771; 0.888]
Thijssen, 2021b	56	65	0.862	[0.753; 0.935]
VandeVrede, 2023a	152	192	0.792	[0.727; 0.847]
Woo, 2023b	80	137	0.584	[0.497; 0.667]
Xiao, 2023	18	27	0.667	[0.460; 0.835]
Random effects model		9276	0.780	[0.738; 0.816]
Heterogeneity: I^2 = 94%, τ^2 = 0.5259, p < 0.01				
**g = Plasma, Tau PET+**				
Barthelemy, 2024b	532	612	0.869	[0.840; 0.895]
Dore, 2022g	162	205	0.790	[0.728; 0.844]
Dore, 2022h	162	201	0.806	[0.744; 0.858]
Dore, 2022i	154	195	0.790	[0.726; 0.845]
Ossenkoppele, 2021f	200	227	0.881	[0.832; 0.920]
Clifford, 2023a	130	502	0.259	[0.221; 0.300]
Clifford, 2023b	190	395	0.481	[0.431; 0.532]
Dore, 2022d	48	111	0.432	[0.339; 0.530]
Dore, 2022e	54	109	0.495	[0.398; 0.593]
Dore, 2022f	38	89	0.427	[0.323; 0.536]
Jonaitis, 2023a	92	115	0.800	[0.715; 0.869]
Ossenkoppele, 2021e	22	52	0.423	[0.287; 0.568]
Ashton, 2024 a	92	125	0.736	[0.650; 0.811]
Ashton, 2024 c	92	110	0.836	[0.754; 0.900]
Dore, 2022a	202	263	0.768	[0.712; 0.818]
Dore, 2022b	222	301	0.738	[0.684; 0.786]
Dore, 2022c	198	271	0.731	[0.674; 0.783]
M-Carlgren, 2024a	170	181	0.939	[0.894; 0.969]
M-Carlgren, 2024b	98	126	0.778	[0.695; 0.847]
Meilke, 2022a	20	40	0.500	[0.338; 0.662]
Meilke, 2022b	40	53	0.755	[0.617; 0.862]
Mendes, 2024d	80	92	0.870	[0.783; 0.931]
Ossenkoppele, 2021d	200	256	0.781	[0.726; 0.830]
Palmqvist, 2020c	300	397	0.756	[0.710; 0.797]
Thijssen, 2021c	134	142	0.944	[0.892; 0.975]
Thijssen, 2021d	44	46	0.957	[0.852; 0.995]
VandeVrede, 2023b	124	152	0.816	[0.745; 0.874]
Random effects model		5368	0.746	[0.670; 0.809]
Heterogeneity: I^2 = 97%, τ^2 = 0.8906, p < 0.01				
**Random effects model**		**18122**	**0.773**	**[0.740; 0.802]**
Heterogeneity: I^2 = 95%, τ^2 = 0.6420, p < 0.01				
Test for subgroup differences: χ^2_3 = 1.26, df = 3 (p = 0.74)				

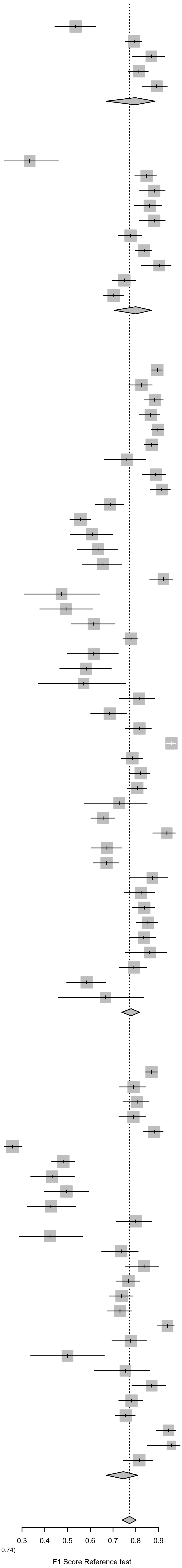

Supplement: Supplementary file 2 — Supporting Information [file ALZ-21-e14458-s002.pdf]
