# Supplementary material for: Diagnostic accuracy of phosphorylated tau217 in detecting Alzheimer's disease pathology among cognitively impaired and unimpaired: A systematic review and meta‐analysis
Source: Alzheimers Dement. 2024 Dec 23;21(2):e14458. doi: 10.1002/alz.14458 (PMC11848338; doi:10.1002/alz.14458)

Supplemental Figure-3 Forest plots showing the diagnostic performance metrics of cerebrospinal fluid (CSF) and plasma phosphorylated tau-217 (p-Tau217), stratified by the reference standard of either amyloid and/or tau PET and cognitive group (unimpaired, impaired, or mixed cohort of both unimpaired and impaired individuals). Cognitively Unimpaired (CU), combined Cognitively Unimpaired and Cognitively Impaired (CU+CI), and Cognitively Impaired (CI); g: group of studies that used cerebrospinal fluid (CSF) or plasma p-Tau217 in predicting PET positivity; I^2: the proportion of variation due to heterogeneity; τ^2: between-study variance in a random-effects model. χ^2_X: chi-square test statistic with x degrees of freedom (df = x).

Panel (A) shows the sensitivity of each study, illustrating the ability of p-Tau217 to correctly identify true positives (either amyloid and/or tau PET positive). Events: number of true positive cases identified by the test; Total: actual total number of positive subjects in the cohort; Proportion: proportion of positive cases— also known as the true positive rate, reflecting the assay's ability to correctly identify positive cases, or its effectiveness in detecting the condition when present (sensitivity). Panel (B) shows the specificity of each study, illustrating the ability of p-Tau217 to correctly identify true negatives. Events: indicate the number of true negative cases detected by the test; Total: actual total number of negative subjects in the cohort; Proportion: proportion of negative cases, detected by the assay (specificity). Panel (C) shows the diagnostic odds ratio (OR), a comprehensive measure of test effectiveness, which combines sensitivity and specificity into a single metric. Events: number of true positive cases identified by the test; Total: actual number of positive cases in the cohort; Proportion: expressed as the DOR, provides the odds of achieving a true positive result versus a false positive. Finally, Panel (D) highlights the F1 score, representing the harmonic mean of sensitivity and precision, providing a balanced evaluation of the test's predictive power in distinguishing PET-positive cases in cognitive subgroups. Events: number of cases where precision and recall both resulted in a truly positive outcome; Total: total number of positive predictions made by the test; Proportion: balance between sensitivity/recall and precision, giving a single measure that accounts for both false positives and false negatives.

Supp, Figure-3A

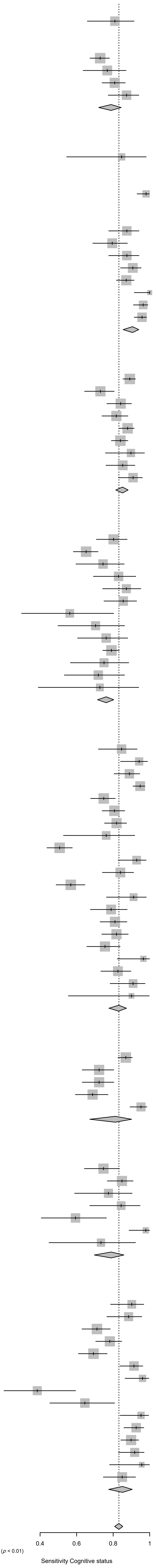

Study	Events	Total	Proportion	95%-CI
**g = CSF, Aß PET+, CU**				
Suarez-Calevt, 2020b	34	42	0.810	[0.659; 0.914]
**g = CSF, Aß PET+, CU+CI**				
Leuzy, 2021c	207	284	0.729	[0.673; 0.780]
Mendes, 2024b	43	56	0.768	[0.636; 0.870]
Palmqvist, 2020b	131	162	0.809	[0.740; 0.866]
Therriault, 2022a	62	71	0.873	[0.773; 0.940]
**Random effects model**		573	0.789	[0.721; 0.844]
Heterogeneity: $I^2 = 63\%$, $\tau^2 = 0.0827$, $p = 0.04$				
**g = CSF, Tau PET+, CU**				
Ossenkoppele, 2021c	11	13	0.846	[0.546; 0.981]
**g = CSF, Tau PET+, CI**				
Ossenkoppele, 2021b	100	102	0.980	[0.931; 0.998]
**g = CSF, Tau PET+, CU+CI**				
Janelidze, 2020a	63	72	0.875	[0.776; 0.941]
Janelidze, 2020b	62	78	0.795	[0.688; 0.878]
Janelidze, 2020c	63	72	0.875	[0.776; 0.941]
Leuzy, 2021a	108	119	0.908	[0.841; 0.953]
Leuzy, 2021b	177	203	0.872	[0.818; 0.915]
Mendes, 2024a	42	42	1.000	[0.916; 1.000]
Ossenkoppele, 2021a	108	112	0.964	[0.911; 0.990]
Palmqvist, 2020a	160	167	0.958	[0.916; 0.983]
**Random effects model**		865	0.906	[0.855; 0.940]
Heterogeneity: $I^2 = 71\%$, $\tau^2 = 0.3357$, $p < 0.01$				
**g = Plasma, Aß PET+, CI**				
Barthelemy, 2024a	311	349	0.891	[0.854; 0.922]
Dore, 2022m	92	126	0.730	[0.644; 0.805]
Dore, 2022n	106	126	0.841	[0.766; 0.900]
Dore, 2022o	103	126	0.817	[0.739; 0.881]
Figdore, 2024a	240	273	0.879	[0.834; 0.915]
Figdore, 2024b	230	274	0.839	[0.790; 0.881]
Niimi, 2024a	35	39	0.897	[0.758; 0.971]
Therriault, 2024b	75	88	0.852	[0.761; 0.919]
Therriault, 2024e	80	88	0.909	[0.829; 0.960]
**Random effects model**		1489	0.851	[0.814; 0.882]
Heterogeneity: $I^2 = 67\%$, $\tau^2 = 0.1026$, $p < 0.01$				
**g = Plasma, Aß PET+, CU**				
Clifford, 2023c	77	96	0.802	[0.708; 0.876]
Clifford, 2023d	133	204	0.652	[0.582; 0.717]
Dore, 2022p	35	47	0.745	[0.597; 0.861]
Dore, 2022q	39	47	0.830	[0.692; 0.924]
Dore, 2022r	41	47	0.872	[0.743; 0.952]
Jonaitis, 2023b	59	69	0.855	[0.750; 0.928]
Meyer, 2024	9	16	0.562	[0.299; 0.802]
Mila-aloma, 2022	19	27	0.704	[0.498; 0.862]
Niimi, 2024c	32	42	0.762	[0.605; 0.879]
Rissman, 2024	268	339	0.791	[0.743; 0.833]
Therriault, 2024c	24	32	0.750	[0.566; 0.885]
Therriault, 2024f	23	32	0.719	[0.533; 0.863]
Thijssen, 2021e	8	11	0.727	[0.390; 0.940]
**Random effects model**		1009	0.762	[0.714; 0.803]
Heterogeneity: $I^2 = 55\%$, $\tau^2 = 0.0872$, $p < 0.01$				
**g = Plasma, Aß PET+, CU+CI**				
Ashton, 2024 b	44	52	0.846	[0.719; 0.931]
Ashton, 2024d	49	52	0.942	[0.841; 0.988]
Asken, 2024	80	90	0.889	[0.805; 0.945]
Brum, 2023	200	211	0.948	[0.909; 0.974]
Dore, 2022j	128	171	0.749	[0.677; 0.812]
Dore, 2022k	138	171	0.807	[0.740; 0.863]
Dore, 2022l	140	171	0.819	[0.753; 0.873]
Gonzales-ortiz, 2023	16	21	0.762	[0.528; 0.918]
Meilke, 2022c	107	211	0.507	[0.438; 0.576]
Mendes, 2024c	52	56	0.929	[0.827; 0.980]
Niimi, 2024b	68	81	0.840	[0.741; 0.912]
Palmqvist, 2020d	92	162	0.568	[0.488; 0.645]
Thanapornsangsuth, 2024	31	34	0.912	[0.763; 0.981]
Therriault, 2022c	56	71	0.789	[0.676; 0.877]
Therriault, 2024a	98	121	0.810	[0.729; 0.876]
Therriault, 2024d	99	121	0.818	[0.738; 0.882]
Thijssen, 2021a	71	94	0.755	[0.656; 0.838]
Thijssen, 2021b	28	29	0.966	[0.822; 0.999]
VandeVrede, 2023a	76	92	0.826	[0.733; 0.897]
Woo, 2023b	40	44	0.909	[0.783; 0.975]
Xiao, 2023	9	10	0.900	[0.555; 0.997]
**Random effects model**		2065	0.830	[0.777; 0.873]
Heterogeneity: $I^2 = 89\%$, $\tau^2 = 0.4824$, $p < 0.01$				
**g = Plasma, Tau PET+, CI**				
Barthelemy, 2024b	266	306	0.869	[0.826; 0.905]
Dore, 2022g	81	112	0.723	[0.631; 0.804]
Dore, 2022h	81	112	0.723	[0.631; 0.804]
Dore, 2022i	77	112	0.688	[0.593; 0.772]
Ossenkoppele, 2021f	100	105	0.952	[0.892; 0.984]
**Random effects model**		747	0.812	[0.672; 0.901]
Heterogeneity: $I^2 = 89\%$, $\tau^2 = 0.6529$, $p < 0.01$				
**g = Plasma, Tau PET+, CU**				
Clifford, 2023a	65	87	0.747	[0.643; 0.834]
Clifford, 2023b	95	112	0.848	[0.768; 0.909]
Dore, 2022d	24	31	0.774	[0.589; 0.904]
Dore, 2022e	27	32	0.844	[0.672; 0.947]
Dore, 2022f	19	32	0.594	[0.406; 0.763]
Jonaitis, 2023a	46	47	0.979	[0.887; 0.999]
Ossenkoppele, 2021e	11	15	0.733	[0.449; 0.922]
**Random effects model**		356	0.789	[0.697; 0.859]
Heterogeneity: $I^2 = 65\%$, $\tau^2 = 0.2406$, $p < 0.01$				
**g = Plasma, Tau PET+, CU+CI**				
Ashton, 2024 a	46	51	0.902	[0.786; 0.967]
Ashton, 2024 c	46	52	0.885	[0.766; 0.956]
Dore, 2022a	101	142	0.711	[0.629; 0.784]
Dore, 2022b	111	142	0.782	[0.705; 0.847]
Dore, 2022c	99	143	0.692	[0.610; 0.767]
M-Carlgren, 2024a	85	93	0.914	[0.838; 0.962]
M-Carlgren, 2024b	49	51	0.961	[0.865; 0.995]
Meilke, 2022a	10	26	0.385	[0.202; 0.594]
Meilke, 2022b	20	31	0.645	[0.454; 0.808]
Mendes, 2024d	40	42	0.952	[0.838; 0.994]
Ossenkoppele, 2021d	100	108	0.926	[0.859; 0.967]
Palmqvist, 2020c	150	167	0.898	[0.842; 0.940]
Thijssen, 2021c	67	73	0.918	[0.830; 0.969]
Thijssen, 2021d	22	23	0.957	[0.781; 0.999]
VandeVrede, 2023b	62	73	0.849	[0.746; 0.922]
**Random effects model**		1217	0.851	[0.775; 0.905]
Heterogeneity: $I^2 = 85\%$, $\tau^2 = 0.8035$, $p < 0.01$				
**Random effects model**		**8478**	**0.832**	**[0.808; 0.853]**
Heterogeneity: $I^2 = 84\%$, $\tau^2 = 0.4154$, $p < 0.01$				
Test for subgroup differences: $\chi^2_{10} = 33.77$, df = 10 ($p < 0.01$)				

Supp, Figure-3B

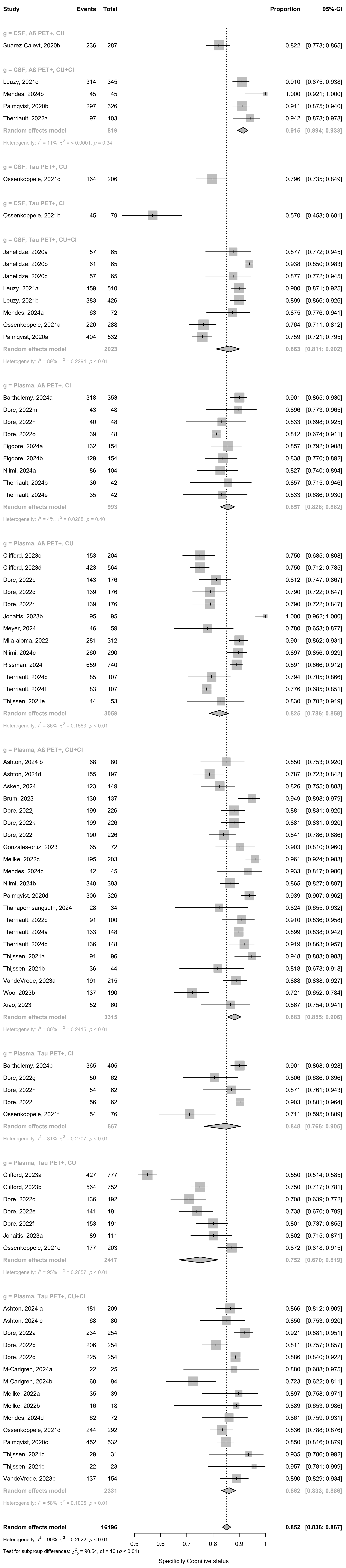

Supp, Figure-3C

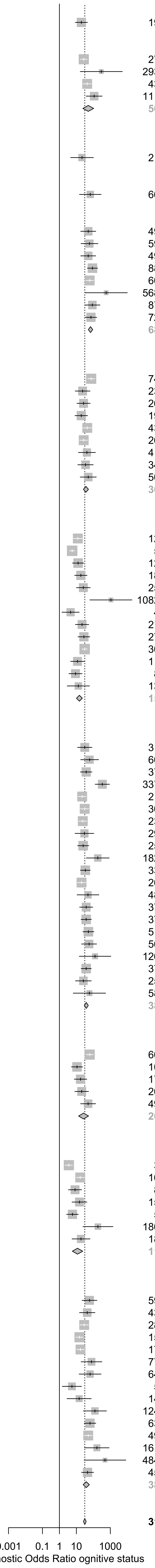

Study	Experimental Events	Experimental Total	Control Events	Control Total	OR	95%-CI
**g = CSF, Aß PET+, CU**						
Suarez-Calevt, 2020b	34	85	8	244	19.66667	[8.59705; 44.98960]
**g = CSF, Aß PET+, CU+CI**						
Leuzy, 2021c	207	238	77	391	27.23000	[17.32227; 42.80458]
Mendes, 2024b	43	43	13	58	293.22222	[16.90866; 5084.92519]
Palmqvist, 2020b	131	160	31	328	43.27809	[25.05733; 74.74830]
Therriault, 2022a	62	68	9	106	111.37037	[37.78181; 328.28919]
Random effects model		509		883	50.16129	[24.12820; 104.28275]
Heterogeneity: I^2 = 63%, τ^2 = 0.3221, p = 0.04						
**g = CSF, Tau PET+, CU**						
Ossenkoppele, 2021c	11	53	2	166	21.47619	[4.58437; 100.60858]
**g = CSF, Tau PET+, CI**						
Ossenkoppele, 2021b	100	134	2	47	66.17647	[15.23330; 287.48364]
**g = CSF, Tau PET+, CU+CI**						
Janelidze, 2020a	63	71	9	66	49.87500	[18.02903; 137.97278]
Janelidze, 2020b	62	66	16	77	59.09375	[18.68777; 186.86396]
Janelidze, 2020c	63	71	9	66	49.87500	[18.02903; 137.97278]
Leuzy, 2021a	108	159	11	470	88.36364	[44.56734; 175.19853]
Leuzy, 2021b	177	220	26	409	60.63596	[36.10429; 101.83608]
Mendes, 2024a	42	51	0	63	568.15789	[32.20685; 10022.81863]
Ossenkoppele, 2021a	108	176	4	224	87.35294	[31.05014; 245.74886]
Palmqvist, 2020a	160	288	7	411	72.14286	[32.98809; 157.77185]
Random effects model		1102		1786	68.09952	[50.52968; 91.77862]
Heterogeneity: I^2 = 0%, τ^2 = < 0.0001, p = 0.79						
**g = Plasma, Aß PET+, CI**						
Barthelemy, 2024a	311	346	38	356	74.35940	[45.78032; 120.77940]
Dore, 2022m	92	97	34	77	23.27059	[8.50816; 63.64717]
Dore, 2022n	106	114	20	60	26.50000	[10.80693; 64.98145]
Dore, 2022o	103	112	23	62	19.40580	[8.25963; 45.59342]
Figdore, 2024a	240	262	33	165	43.63636	[24.43767; 77.91792]
Figdore, 2024b	230	255	44	173	26.97273	[15.77912; 46.10702]
Niimi, 2024a	35	53	4	90	41.80556	[13.20443; 132.35745]
Therriault, 2024b	75	81	13	49	34.61538	[12.16439; 98.50264]
Therriault, 2024e	80	87	8	43	50.00000	[16.82135; 148.62068]
Random effects model		1407		1075	36.53513	[25.93077; 51.47611]
Heterogeneity: I^2 = 40%, τ^2 = 0.1092, p = 0.10						
**g = Plasma, Aß PET+, CU**						
Clifford, 2023c	77	128	19	172	12.15789	[6.71444; 22.01441]
Clifford, 2023d	133	274	71	494	5.61972	[3.97833; 7.93831]
Dore, 2022p	35	68	12	155	12.63889	[5.92815; 26.94626]
Dore, 2022q	39	76	8	147	18.31419	[7.88512; 42.53702]
Dore, 2022r	41	78	6	145	25.67117	[10.12595; 65.08120]
Jonaitis, 2023b	59	59	10	105	1082.33333	[62.26696; 18813.27541]
Meyer, 2024	9	22	7	53	4.54945	[1.42067; 14.56885]
Mila-aloma, 2022	19	50	8	289	21.52823	[8.70461; 53.24360]
Niimi, 2024c	32	62	10	270	27.73333	[12.40702; 61.99213]
Rissman, 2024	268	349	71	730	30.70979	[21.66581; 43.52900]
Therriault, 2024c	24	46	8	93	11.59091	[4.58520; 29.30063]
Therriault, 2024f	23	47	9	92	8.83796	[3.61294; 21.61940]
Thijssen, 2021e	8	17	3	47	13.03704	[2.88507; 58.91168]
Random effects model		1276		2792	15.03226	[10.09969; 22.37383]
Heterogeneity: I^2 = 82%, τ^2 = 0.3283, p < 0.01						
**g = Plasma, Aß PET+, CU+CI**						
Ashton, 2024 b	44	56	8	76	31.16667	[11.79529; 82.35162]
Ashton, 2024d	49	91	3	158	60.27778	[17.89308; 203.06232]
Asken, 2024	80	106	10	133	37.84615	[17.31845; 82.70552]
Brum, 2023	200	207	11	141	337.66234	[127.61656; 893.42510]
Dore, 2022j	128	155	43	242	21.93971	[12.91348; 37.27505]
Dore, 2022k	138	165	33	232	30.82155	[17.72897; 53.58280]
Dore, 2022l	140	176	31	221	23.83513	[14.06424; 40.39416]
Gonzales-ortiz, 2023	16	23	5	70	29.71429	[8.33368; 105.94827]
Meilke, 2022c	107	115	104	299	25.07812	[11.76599; 53.45174]
Mendes, 2024c	52	55	4	46	182.00000	[38.58321; 858.50806]
Niimi, 2024b	68	121	13	353	33.55588	[17.34120; 64.93190]
Palmqvist, 2020d	92	112	70	376	20.10857	[11.61474; 34.81393]
Thanapornsangsuth, 2024	31	37	3	31	48.22222	[11.00901; 211.22545]
Therriault, 2022c	56	65	15	106	37.74815	[15.48725; 92.00618]
Therriault, 2024a	98	113	23	156	37.77971	[18.74480; 76.14413]
Therriault, 2024d	99	111	22	158	51.00000	[24.10223; 107.91531]
Thijssen, 2021a	71	76	23	114	56.18261	[20.34663; 155.13558]
Thijssen, 2021b	28	36	1	37	126.00000	[14.87332; 1067.41500]
VandeVrede, 2023a	76	100	16	207	37.80208	[19.03365; 75.07743]
Woo, 2023b	40	93	4	141	25.84906	[8.81691; 75.78322]
Xiao, 2023	9	17	1	53	58.50000	[6.50807; 525.84705]
Random effects model		2030		3350	38.35094	[29.31000; 50.18065]
Heterogeneity: I^2 = 50%, τ^2 = 0.1834, p < 0.01						
**g = Plasma, Tau PET+, CI**						
Barthelemy, 2024b	266	306	40	405	60.68125	[38.08271; 96.68993]
Dore, 2022g	81	93	31	81	10.88710	[5.12292; 23.13696]
Dore, 2022h	81	89	31	85	17.63710	[7.53763; 41.26856]
Dore, 2022i	77	83	35	91	20.53333	[8.08592; 52.14224]
Ossenkoppele, 2021f	100	122	5	59	49.09091	[17.59792; 136.94327]
Random effects model		693		721	26.47776	[13.45297; 52.11278]
Heterogeneity: I^2 = 78%, τ^2 = 0.4279, p < 0.01						
**g = Plasma, Tau PET+, CU**						
Clifford, 2023a	65	415	22	449	3.60455	[2.17826; 5.96474]
Clifford, 2023b	95	283	17	581	16.76471	[9.75112; 28.82289]
Dore, 2022d	24	80	7	143	8.32653	[3.39345; 20.43085]
Dore, 2022e	27	77	5	146	15.22800	[5.56135; 41.69704]
Dore, 2022f	19	57	13	166	5.88462	[2.67108; 12.96431]
Jonaitis, 2023a	46	68	1	90	186.09091	[24.30938; 1424.54573]
Ossenkoppele, 2021e	11	37	4	181	18.72115	[5.54833; 63.16889]
Random effects model		1017		1756	11.63815	[5.75979; 23.51587]
Heterogeneity: I^2 = 80%, τ^2 = 0.6545, p < 0.01						
**g = Plasma, Tau PET+, CU+CI**						
Ashton, 2024 a	46	74	5	186	59.47143	[21.76708; 162.48621]
Ashton, 2024 c	46	58	6	74	43.44444	[15.21831; 124.02295]
Dore, 2022a	101	121	41	275	28.82195	[16.08449; 51.64633]
Dore, 2022b	111	159	31	237	15.36694	[9.25409; 25.51767]
Dore, 2022c	99	128	44	269	17.45690	[10.32652; 29.51075]
M-Carlgren, 2024a	85	88	8	30	77.91667	[19.07439; 318.28049]
M-Carlgren, 2024b	49	75	2	70	64.07692	[14.52266; 282.72044]
Meilke, 2022a	10	14	16	51	5.46875	[1.48791; 20.10011]
Meilke, 2022b	20	22	11	27	14.54545	[2.81079; 75.27065]
Mendes, 2024d	40	50	2	64	124.00000	[25.81428; 595.63924]
Ossenkoppele, 2021d	100	148	8	252	63.54167	[29.01688; 139.14466]
Palmqvist, 2020c	150	230	17	469	49.85294	[28.61739; 86.84635]
Thijssen, 2021c	67	69	6	35	161.91667	[30.83149; 850.33203]
Thijssen, 2021d	22	23	1	23	484.00000	[28.44526; 8235.32532]
VandeVrede, 2023b	62	79	11	148	45.42246	[20.09387; 102.67810]
Random effects model		1338		2210	38.16188	[25.02416; 58.19690]
Heterogeneity: I^2 = 66%, τ^2 = 0.3939, p < 0.01						
**Random effects model**		**9644**		**15030**	**31.29452**	**[26.01787; 37.64131]**

Heterogeneity: I^2 = 79%, τ^2 = 0.5050, p < 0.01

Test for subgroup differences: χ^2_{10} = 50.97, df = 10 (p < 0.01)

Supp, Figure-3D

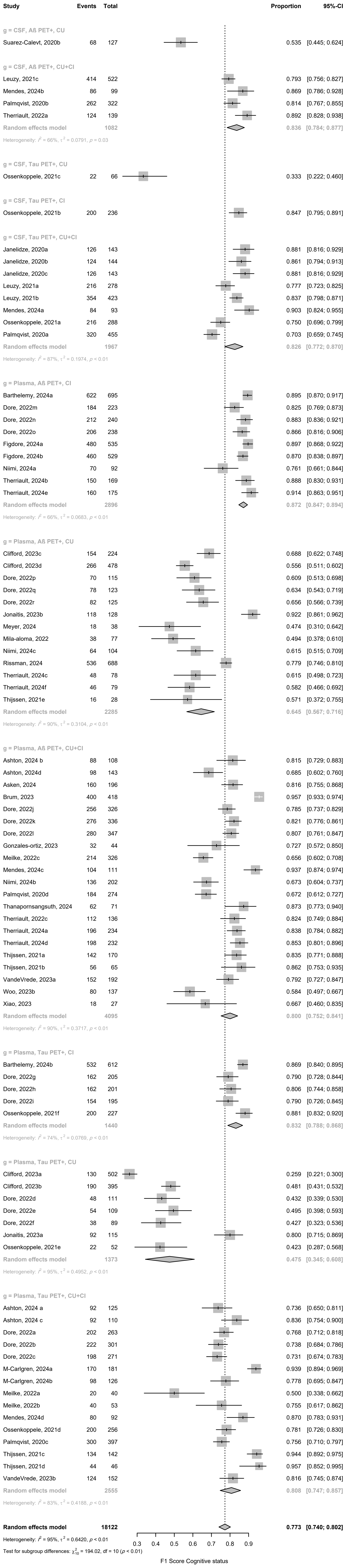

Supplement: Supplementary file 3 — Supporting Information [file ALZ-21-e14458-s010.pdf]
