# Supplementary material for: Diagnostic accuracy of phosphorylated tau217 in detecting Alzheimer's disease pathology among cognitively impaired and unimpaired: A systematic review and meta‐analysis
Source: Alzheimers Dement. 2024 Dec 23;21(2):e14458. doi: 10.1002/alz.14458 (PMC11848338; doi:10.1002/alz.14458)

Supplemental Figure-4 Forest plots illustrating the diagnostic performance of four platforms—Lilly, Simoa-ALZpath, Simoa-Jansson R&D, and Mass Spectrometry—for phosphorylated tau-217 (p-Tau217) in detecting AD pathology, using either amyloid and/or tau PET as the reference. g: group of studies that used cerebrospinal fluid (CSF) or plasma p-Tau217 in predicting PET positivity, stratified by assay type. g: group of studies that used cerebrospinal fluid (CSF) or plasma biomarkers; I^2: the proportion of variation due to heterogeneity; τ^2: between-study variance in a random-effects model. χ^2_X: chi-square test statistic with x degrees of freedom (df = x).

Panel (A) presents the sensitivity of each platform, reflecting its ability to correctly identify true positive cases. Events: number of subjects testing positive; Total: actual total number of positive subjects in the cohort; Proportion: proportion of positive cases, detected by the assay (sensitivity). Panel (B) shows the specificity, highlighting how well each platform correctly identified true negatives. Events: number of subjects testing negative; Total: actual total number of negative subjects in the cohort; Proportion: proportion of negative cases, detected by the assay (specificity). Panel (C) provides the diagnostic odds ratio (OR), which combines sensitivity and specificity to evaluate overall test performance. Events: number of true positive cases identified by the test; Total: actual number of positive cases in the cohort; Proportion: expressed as the DOR, provides the odds of achieving a true positive result versus a false positive. Panel (D) displays the F1 score, the mean of sensitivity and precision, offering a balanced assessment of each platform's accuracy in detecting AD pathology. Events: number of cases where precision and recall both resulted in a truly positive outcome; Total: total number of positive predictions made by the test; Proportion: balance between sensitivity/recall and precision, giving a single measure that accounts for both false positives and false negatives.

*Note that combining various mass spectrometry techniques as a single method allowed us to obtain a general estimate for diagnostic performance when employing PET as the reference standard. Our analysis more precisely categorized the studies into three subgroups in **Supplemental Figure-5**.

Supp, Figure-4A

Study	Events	Total	Proportion	95%-CI
g = CSF, MSD based-Lilly				
Ossenkoppele, 2021c	100	102	0.980	[0.931; 0.998]
Ossenkoppele, 2021e	11	13	0.846	[0.546; 0.981]
Janelidze, 2020a	63	72	0.875	[0.776; 0.941]
Leuzy, 2021a*	108	119	0.908	[0.841; 0.953]
Leuzy, 2021b*	177	203	0.872	[0.818; 0.915]
Mendes, 2024b*	42	42	1.000	[0.916; 1.000]
Mendes, 2024d*	43	56	0.768	[0.636; 0.870]
Ossenkoppele, 2021d	108	112	0.964	[0.911; 0.990]
Palmqvist, 2020b*	160	167	0.958	[0.916; 0.983]
Palmqvist, 2020c*	131	162	0.809	[0.740; 0.866]
Random effects model		1048	0.909	[0.850; 0.946]
Heterogeneity: I^2 = 78%, τ^2 = 0.5839, p < 0.01				
g = CSF, Simoa_Homebrew				
Suarez-Calevt, 2020b	34	42	0.810	[0.659; 0.914]
g = CSF, Simoa_Janssen R&D				
Therriault, 2022c	62	71	0.873	[0.773; 0.940]
g = Plasma, Lumipluse-Fujirebio				
Figdore, 2024b	240	273	0.879	[0.834; 0.915]
g = Plasma, Mass Spectromerty				
Barthelemy, 2024a*	311	349	0.891	[0.854; 0.922]
Barthelemy, 2024b*	266	306	0.869	[0.826; 0.905]
Rissman, 2024	268	339	0.791	[0.743; 0.833]
Gonzales-ortiz, 2023	16	21	0.762	[0.528; 0.918]
Random effects model		1015	0.845	[0.781; 0.892]
Heterogeneity: I^2 = 81%, τ^2 = 0.1322, p < 0.01				
g = Plasma, MSD based-Lilly				
Niimi, 2024b	35	39	0.897	[0.758; 0.971]
Ossenkoppele, 2021c	100	105	0.952	[0.892; 0.984]
Clifford, 2023a*	95	112	0.848	[0.768; 0.909]
Clifford, 2023d*	133	204	0.652	[0.582; 0.717]
Jonaitis, 2023a*	46	47	0.979	[0.887; 0.999]
Jonaitis, 2023b*	59	69	0.855	[0.750; 0.928]
Mila-aloma, 2022	19	27	0.704	[0.498; 0.862]
Niimi, 2024a	32	42	0.762	[0.605; 0.879]
Ossenkoppele, 2021b	11	15	0.733	[0.449; 0.922]
Thijssen, 2021b	8	11	0.727	[0.390; 0.940]
Brum, 2023	200	211	0.948	[0.909; 0.974]
Janelidze, 2021c	45	57	0.789	[0.661; 0.886]
M-Carlgren, 2024a*	85	93	0.914	[0.838; 0.962]
M-Carlgren, 2024b*	49	51	0.961	[0.865; 0.995]
Meilke, 2022b*	20	31	0.645	[0.454; 0.808]
Meilke, 2022c*	107	211	0.507	[0.438; 0.576]
Mendes, 2024a*	40	42	0.952	[0.838; 0.994]
Mendes, 2024c*	52	56	0.929	[0.827; 0.980]
Niimi, 2024c	68	81	0.840	[0.741; 0.912]
Ossenkoppele, 2021a	100	108	0.926	[0.859; 0.967]
Palmqvist, 2020a*	150	167	0.898	[0.842; 0.940]
Palmqvist, 2020d*	92	162	0.568	[0.488; 0.645]
Thanapornsangsuth, 2024	31	34	0.912	[0.763; 0.981]
Thijssen, 2021d*	28	29	0.966	[0.822; 0.999]
Thijssen, 2021e*	22	23	0.957	[0.781; 0.999]
VandeVrede, 2023a*	76	92	0.826	[0.733; 0.897]
VandeVrede, 2023b*	62	73	0.849	[0.746; 0.922]
Random effects model		2192	0.855	[0.803; 0.895]
Heterogeneity: I^2 = 90%, τ^2 = 0.7299, p < 0.01				
g = Plasma, Simoa_ALZpath				
Figdore, 2024a	230	274	0.839	[0.790; 0.881]
Therriault, 2024a	80	88	0.909	[0.829; 0.960]
Meyer, 2024	9	16	0.562	[0.299; 0.802]
Therriault, 2024b	23	32	0.719	[0.533; 0.863]
Ashton, 2024 a*	46	51	0.902	[0.786; 0.967]
Ashton, 2024 b*	44	52	0.846	[0.719; 0.931]
Ashton, 2024 c*	46	52	0.885	[0.766; 0.956]
Ashton, 2024d	49	52	0.942	[0.841; 0.988]
Asken, 2024	80	90	0.889	[0.805; 0.945]
Therriault, 2024c	99	121	0.818	[0.738; 0.882]
Xiao, 2023	9	10	0.900	[0.555; 0.997]
Random effects model		838	0.850	[0.800; 0.890]
Heterogeneity: I^2 = 55%, τ^2 = 0.1920, p = 0.01				
g = Plasma, Simoa_Janssen R&D				
Dore, 2022j	77	112	0.688	[0.593; 0.772]
Dore, 2022o*	103	126	0.817	[0.739; 0.881]
Feizpour, 2024c	109	131	0.832	[0.757; 0.892]
Therriault, 2024d	75	88	0.852	[0.761; 0.919]
Dore, 2022b*	27	32	0.844	[0.672; 0.947]
Dore, 2022e*	35	47	0.745	[0.597; 0.861]
Therriault, 2024e	24	32	0.750	[0.566; 0.885]
Dore, 2022i*	111	142	0.782	[0.705; 0.847]
Dore, 2022p*	128	171	0.749	[0.677; 0.812]
Feizpour, 2024a	70	80	0.875	[0.782; 0.938]
Therriault, 2022a*	56	71	0.789	[0.676; 0.877]
Therriault, 2024f*	98	121	0.810	[0.729; 0.876]
Woo, 2023a*	80	89	0.899	[0.817; 0.953]
Woo, 2023b*	40	44	0.909	[0.783; 0.975]
Random effects model		1286	0.807	[0.772; 0.837]
Heterogeneity: I^2 = 52%, τ^2 = 0.0781, p = 0.01				
**Random effects model**		**6765**	**0.851**	**[0.827; 0.872]**

Heterogeneity: I^2 = 85%, τ^2 = 0.4072, p < 0.01

Test for subgroup differences: χ^2_7 = 14.01, df = 7 (p = 0.05)

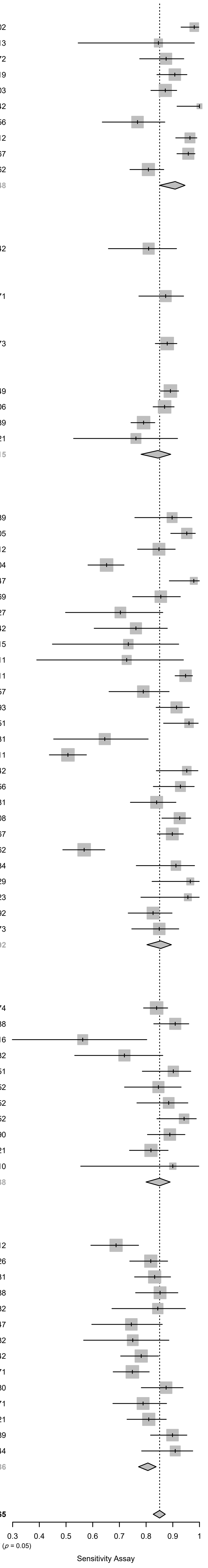

Supp, Figure-4B

Study	Events	Total	Proportion	95%-CI
**g = CSF, MSD based-Lilly**				
Ossenkoppele, 2021c	45	79	0.570	[0.453; 0.681]
Ossenkoppele, 2021e	164	206	0.796	[0.735; 0.849]
Janelidze, 2020a	57	65	0.877	[0.772; 0.945]
Leuzy, 2021a*	459	510	0.900	[0.871; 0.925]
Leuzy, 2021b*	383	426	0.899	[0.866; 0.926]
Mendes, 2024b*	63	72	0.875	[0.776; 0.941]
Mendes, 2024d*	45	45	1.000	[0.921; 1.000]
Ossenkoppele, 2021d	220	288	0.764	[0.711; 0.812]
Palmqvist, 2020b*	404	532	0.759	[0.721; 0.795]
Palmqvist, 2020c*	297	326	0.911	[0.875; 0.940]
**Random effects model**		**2549**	**0.842**	**[0.772; 0.894]**
Heterogeneity: I^2 = 92%, τ^2 = 0.4514, $p < 0.01$				
**g = CSF, Simoa_Homebrew**				
Suarez-Calevt, 2020b	236	287	0.822	[0.773; 0.865]
**g = CSF, Simoa_Janssen R&D**				
Therriault, 2022c	97	103	0.942	[0.878; 0.978]
**g = Plasma, Lumipluse-Fujirebio**				
Figdore, 2024b	132	154	0.857	[0.792; 0.908]
**g = Plasma, Mass Spectromertry**				
Barthelemy, 2024a*	318	353	0.901	[0.865; 0.930]
Barthelemy, 2024b*	365	405	0.901	[0.868; 0.928]
Rissman, 2024	659	740	0.891	[0.866; 0.912]
Gonzales-ortiz, 2023	65	72	0.903	[0.810; 0.960]
**Random effects model**		**1570**	**0.896**	**[0.880; 0.910]**
Heterogeneity: I^2 = 0%, τ^2 = 0, [illegible]				
**g = Plasma, MSD based-Lilly**				
Niimi, 2024b	86	104	0.827	[0.740; 0.894]
Ossenkoppele, 2021c	54	76	0.711	[0.595; 0.809]
Clifford, 2023a*	564	752	0.750	[0.717; 0.781]
Clifford, 2023d*	423	564	0.750	[0.712; 0.785]
Jonaitis, 2023a*	89	111	0.802	[0.715; 0.871]
Jonaitis, 2023b*	95	95	1.000	[0.962; 1.000]
Mila-aloma, 2022	281	312	0.901	[0.862; 0.931]
Niimi, 2024a	260	290	0.897	[0.856; 0.929]
Ossenkoppele, 2021b	177	203	0.872	[0.818; 0.915]
Thijssen, 2021b	44	53	0.830	[0.702; 0.919]
Brum, 2023	130	137	0.949	[0.898; 0.979]
Janelidze, 2021c	117	148	0.791	[0.716; 0.853]
M-Carlgren, 2024a*	22	25	0.880	[0.688; 0.975]
M-Carlgren, 2024b*	68	94	0.723	[0.622; 0.811]
Meilke, 2022b*	16	18	0.889	[0.653; 0.986]
Meilke, 2022c*	195	203	0.961	[0.924; 0.983]
Mendes, 2024a*	62	72	0.861	[0.759; 0.931]
Mendes, 2024c*	42	45	0.933	[0.817; 0.986]
Niimi, 2024c	340	393	0.865	[0.827; 0.897]
Ossenkoppele, 2021a	244	292	0.836	[0.788; 0.876]
Palmqvist, 2020a*	452	532	0.850	[0.816; 0.879]
Palmqvist, 2020d*	306	326	0.939	[0.907; 0.962]
Thanapornsangsuth, 2024	28	34	0.824	[0.655; 0.932]
Thijssen, 2021d*	36	44	0.818	[0.673; 0.918]
Thijssen, 2021e*	22	23	0.957	[0.781; 0.999]
VandeVrede, 2023a*	191	215	0.888	[0.838; 0.927]
VandeVrede, 2023b*	137	154	0.890	[0.829; 0.934]
**Random effects model**		**5315**	**0.862**	**[0.831; 0.887]**
Heterogeneity: I^2 = 86%, τ^2 = 0.2797, $p < 0.01$				
**g = Plasma, Simoa_ALZpath**				
Figdore, 2024a	129	153	0.843	[0.776; 0.897]
Therriault, 2024a	35	42	0.833	[0.686; 0.930]
Meyer, 2024	46	59	0.780	[0.653; 0.877]
Therriault, 2024b	83	107	0.776	[0.685; 0.851]
Ashton, 2024 a*	181	209	0.866	[0.812; 0.909]
Ashton, 2024 b*	68	80	0.850	[0.753; 0.920]
Ashton, 2024 c*	68	80	0.850	[0.753; 0.920]
Ashton, 2024d	155	197	0.787	[0.723; 0.842]
Asken, 2024	123	149	0.826	[0.755; 0.883]
Therriault, 2024c	136	148	0.919	[0.863; 0.957]
Xiao, 2023	52	60	0.867	[0.754; 0.941]
**Random effects model**		**1284**	**0.836**	**[0.807; 0.862]**
Heterogeneity: I^2 = 40%, τ^2 = 0.0465, p = 0.08				
**g = Plasma, Simoa_Janssen R&D**				
Dore, 2022j	56	62	0.903	[0.801; 0.964]
Dore, 2022o*	39	48	0.812	[0.674; 0.911]
Feizpour, 2024c	53	62	0.855	[0.742; 0.931]
Therriault, 2024d	36	42	0.857	[0.715; 0.946]
Dore, 2022b*	141	191	0.738	[0.670; 0.799]
Dore, 2022e*	143	176	0.812	[0.747; 0.867]
Therriault, 2024e	85	107	0.794	[0.705; 0.866]
Dore, 2022i*	206	254	0.811	[0.757; 0.857]
Dore, 2022p*	199	226	0.881	[0.831; 0.920]
Feizpour, 2024a	161	192	0.839	[0.779; 0.888]
Therriault, 2022a*	91	100	0.910	[0.836; 0.958]
Therriault, 2024f*	133	148	0.899	[0.838; 0.942]
Woo, 2023a*	132	145	0.910	[0.852; 0.951]
Woo, 2023b*	137	190	0.721	[0.652; 0.784]
**Random effects model**		**1943**	**0.840**	**[0.804; 0.871]**
Heterogeneity: I^2 = 74%, τ^2 = 0.1524, $p < 0.01$				
**Random effects model**		**13205**	**0.853**	**[0.837; 0.869]**
Heterogeneity: I^2 = 84%, τ^2 = 0.2161, $p < 0.01$				
Test for subgroup differences: χ^2_7 = 30.06, df = 7 ($p < 0.01$)				

Supp, Figure-6A

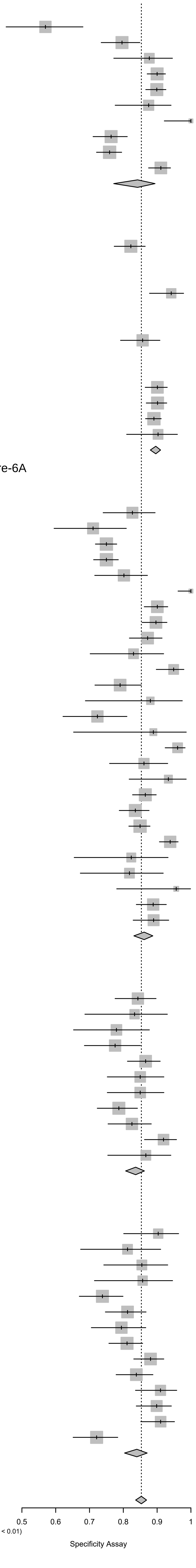

Supp, Figure-4C

Study	Experimental Events	Experimental Total	Control Events	Control Total	OR	95%-CI
**g = CSF, MSD based-Lilly**						
Ossenkoppele, 2021c	100	134	2	47	66.17647	[15.23330; 287.48364]
Ossenkoppele, 2021e	11	53	2	166	21.47619	[4.58437; 100.60858]
Janelidze, 2020a	63	71	9	66	49.87500	[18.02903; 137.97278]
Leuzy, 2021a*	108	159	11	470	88.36364	[44.56734; 175.19853]
Leuzy, 2021b*	177	220	26	409	60.63596	[36.10429; 101.83608]
Mendes, 2024b*	42	51	0	63	568.15789	[32.20685; 10022.81863]
Mendes, 2024d*	43	43	13	58	293.22222	[16.90866; 5084.92519]
Ossenkoppele, 2021d	108	176	4	224	87.35294	[31.05014; 245.74886]
Palmqvist, 2020b*	160	288	7	411	72.14286	[32.98809; 157.77185]
Palmqvist, 2020c*	131	160	31	328	43.27809	[25.05733; 74.74830]
Random effects model		1355		2242	61.43449	[46.96484; 80.36218]
Heterogeneity: I^2 = 0%, τ^2 = < 0.0001, p = 0.47						
**g = CSF, Simoa_Homebrew**						
Suarez-Calevt, 2020b	34	85	8	244	19.66667	[8.59705; 44.98960]
**g = CSF, Simoa_Janssen R&D**						
Therriault, 2022c	62	68	9	106	111.37037	[37.78181; 328.28919]
**g = Plasma, Lumipluse-Fujirebio**						
Figdore, 2024b	240	262	33	165	43.63636	[24.43767; 77.91792]
**g = Plasma, Mass Spectromertry**						
Barthelemy, 2024a*	311	346	38	356	74.35940	[45.78032; 120.77940]
Barthelemy, 2024b*	266	306	40	405	60.68125	[38.08271; 96.68993]
Rissman, 2024	268	349	71	730	30.70979	[21.66581; 43.52900]
Gonzales-ortiz, 2023	16	23	5	70	29.71429	[8.33368; 105.94827]
Random effects model		1024		1561	47.73360	[29.49703; 77.24493]
Heterogeneity: I^2 = 72%, τ^2 = 0.1532, p = 0.01						
**g = Plasma, MSD based-Lilly**						
Niimi, 2024b	35	53	4	90	41.80556	[13.20443; 132.35745]
Ossenkoppele, 2021c	100	122	5	59	49.09091	[17.59792; 136.94327]
Clifford, 2023a*	95	283	17	581	16.76471	[9.75112; 28.82289]
Clifford, 2023d*	133	274	71	494	5.61972	[3.97833; 7.93831]
Jonaitis, 2023a*	46	68	1	90	186.09091	[24.30938; 1424.54573]
Jonaitis, 2023b*	59	59	10	105	1082.33333	[62.26696; 18813.27541]
Mila-aloma, 2022	19	50	8	289	21.52823	[8.70461; 53.24360]
Niimi, 2024a	32	62	10	270	27.73333	[12.40702; 61.99213]
Ossenkoppele, 2021b	11	37	4	181	18.72115	[5.54833; 63.16889]
Thijssen, 2021b	8	17	3	47	13.03704	[2.88507; 58.91168]
Brum, 2023	200	207	11	141	337.66234	[127.61658; 893.42510]
Janelidze, 2021c	45	76	12	129	14.15323	[6.68668; 29.95715]
M-Carlgren, 2024a*	85	88	8	30	77.91667	[19.07439; 318.28049]
M-Carlgren, 2024b*	49	75	2	70	64.07692	[14.52266; 282.72044]
Meilke, 2022b*	20	22	11	27	14.54545	[2.81079; 75.27065]
Meilke, 2022c*	107	115	104	299	25.07812	[11.76599; 53.45174]
Mendes, 2024a*	40	50	2	64	124.00000	[25.81428; 595.63924]
Mendes, 2024c*	52	55	4	46	182.00000	[38.58321; 858.50806]
Niimi, 2024c	68	121	13	353	33.55588	[17.34120; 64.93190]
Ossenkoppele, 2021a	100	148	8	252	63.54167	[29.01688; 139.14466]
Palmqvist, 2020a*	150	230	17	469	49.85294	[28.61739; 86.84635]
Palmqvist, 2020d*	92	112	70	376	20.10857	[11.61474; 34.81393]
Thanapornsangsuth, 2024	31	37	3	31	48.22222	[11.00901; 211.22545]
Thijssen, 2021d*	28	36	1	37	126.00000	[14.87332; 1067.41500]
Thijssen, 2021e*	22	23	1	23	484.00000	[28.44526; 8235.32532]
VandeVrede, 2023a*	76	100	16	207	37.80208	[19.03365; 75.07743]
VandeVrede, 2023b*	62	79	11	148	45.42246	[20.09387; 102.67810]
Random effects model		2599		4908	39.89003	[27.09247; 58.73273]
Heterogeneity: I^2 = 83%, τ^2 = 0.7024, p < 0.01						
**g = Plasma, Simoa_ALZpath**						
Figdore, 2024a	230	254	44	173	28.09659	[16.33927; 48.31418]
Therriault, 2024a	80	87	8	43	50.00000	[16.82135; 148.62068]
Meyer, 2024	9	22	7	53	4.54945	[1.42067; 14.56885]
Therriault, 2024b	23	47	9	92	8.83796	[3.61294; 21.61940]
Ashton, 2024 a*	46	74	5	186	59.47143	[21.76708; 162.48621]
Ashton, 2024 b*	44	56	8	76	31.16667	[11.79529; 82.35162]
Ashton, 2024 c*	46	58	6	74	43.44444	[15.21831; 124.02295]
Ashton, 2024d	49	91	3	158	60.27778	[17.89308; 203.06232]
Asken, 2024	80	106	10	133	37.84615	[17.31845; 82.70552]
Therriault, 2024c	99	111	22	158	51.00000	[24.10223; 107.91531]
Xiao, 2023	9	17	1	53	58.50000	[6.50807; 525.84705]
Random effects model		923		1199	30.68724	[19.16917; 49.12611]
Heterogeneity: I^2 = 59%, τ^2 = 0.3690, p < 0.01						
**g = Plasma, Simoa_Janssen R&D**						
Dore, 2022j	77	83	35	91	20.53333	[8.08592; 52.14224]
Dore, 2022o*	103	112	23	62	19.40580	[8.25963; 45.59342]
Feizpour, 2024c	109	118	22	75	29.17677	[12.56926; 67.72743]
Therriault, 2024d	75	81	13	49	34.61538	[12.16439; 98.50264]
Dore, 2022b*	27	77	5	146	15.22800	[5.56135; 41.69704]
Dore, 2022e*	35	68	12	155	12.63889	[5.92815; 26.94626]
Therriault, 2024e	24	46	8	93	11.59091	[4.58520; 29.30063]
Dore, 2022i*	111	159	31	237	15.36694	[9.25409; 25.51767]
Dore, 2022p*	128	155	43	242	21.93971	[12.91348; 37.27505]
Feizpour, 2024a	70	101	10	171	36.35484	[16.89973; 78.20685]
Therriault, 2022a*	56	65	15	106	37.74815	[15.48725; 92.00618]
Therriault, 2024f*	98	113	23	156	37.77971	[18.74480; 76.14413]
Woo, 2023a*	80	93	9	141	90.25641	[36.91202; 220.69286]
Woo, 2023b*	40	93	4	141	25.84906	[8.81691; 75.78322]
Random effects model		1364		1865	24.40646	[18.42857; 32.32348]
Heterogeneity: I^2 = 41%, τ^2 = 0.1151, p = 0.05						
**Random effects model**		**7680**		**12290**	**36.43330**	**[30.07743; 44.13226]**
Heterogeneity: I^2 = 76%, τ^2 = 0.4035, p < 0.01						
Test for subgroup differences: χ^2_7 = 29.75, df = 7 (p < 0.01)						

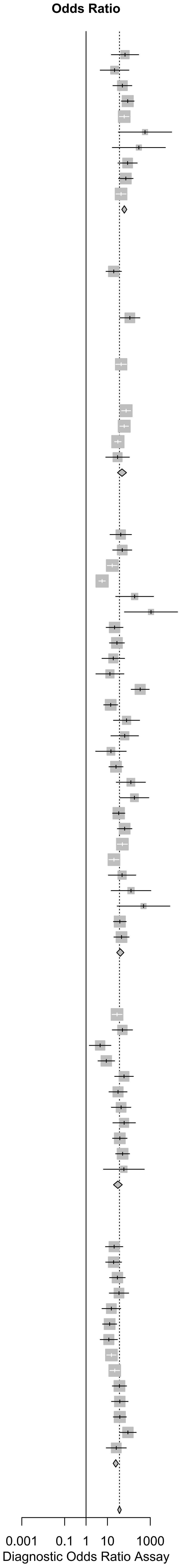

Supp, Figure-4D

Study	Events	Total	Proportion	95%-CI
**g = CSF, MSD based-Lilly**				
Ossenkoppele, 2021c	200	236	0.847	[0.795; 0.891]
Ossenkoppele, 2021e	22	66	0.333	[0.222; 0.460]
Janelidze, 2020a	126	143	0.881	[0.816; 0.929]
Leuzy, 2021a*	216	278	0.777	[0.723; 0.825]
Leuzy, 2021b*	354	423	0.837	[0.798; 0.871]
Mendes, 2024b*	84	93	0.903	[0.824; 0.955]
Mendes, 2024d*	86	99	0.869	[0.786; 0.928]
Ossenkoppele, 2021d	216	288	0.750	[0.696; 0.799]
Palmqvist, 2020b*	320	455	0.703	[0.659; 0.745]
Palmqvist, 2020c*	262	322	0.814	[0.767; 0.855]
Random effects model		2403	0.792	[0.699; 0.862]
Heterogeneity: $I^2 = 92\%$, $\tau^2 = 0.5933$, $p < 0.01$				
**g = CSF, Simoa_Homebrew**				
Suarez-Calevt, 2020b	68	127	0.535	[0.445; 0.624]
**g = CSF, Simoa_Janssen R&D**				
Therriault, 2022c	124	139	0.892	[0.828; 0.938]
**g = Plasma, Lumipluse-Fujirebio**				
Figdore, 2024b	480	535	0.897	[0.868; 0.922]
**g = Plasma, Mass Spectromertry**				
Barthelemy, 2024a*	622	695	0.895	[0.870; 0.917]
Barthelemy, 2024b*	532	612	0.869	[0.840; 0.895]
Rissman, 2024	536	688	0.779	[0.746; 0.810]
Gonzales-ortiz, 2023	32	44	0.727	[0.572; 0.850]
Random effects model		2039	0.834	[0.752; 0.893]
Heterogeneity: $I^2 = 93\%$, $\tau^2 = 0.2341$, $p < 0.01$				
**g = Plasma, MSD based-Lilly**				
Niimi, 2024b	70	92	0.761	[0.661; 0.844]
Ossenkoppele, 2021c	200	227	0.881	[0.832; 0.920]
Clifford, 2023a*	190	395	0.481	[0.431; 0.532]
Clifford, 2023d*	266	478	0.556	[0.511; 0.602]
Jonaitis, 2023a*	92	115	0.800	[0.715; 0.869]
Jonaitis, 2023b*	118	128	0.922	[0.861; 0.962]
Mila-aloma, 2022	38	77	0.494	[0.378; 0.610]
Niimi, 2024a	64	104	0.615	[0.515; 0.709]
Ossenkoppele, 2021b	22	52	0.423	[0.287; 0.568]
Thijssen, 2021b	16	28	0.571	[0.372; 0.755]
Brum, 2023	400	418	0.957	[0.933; 0.974]
Janelidze, 2021c	90	133	0.677	[0.590; 0.755]
M-Carlgren, 2024a*	170	181	0.939	[0.894; 0.969]
M-Carlgren, 2024b*	98	126	0.778	[0.695; 0.847]
Meilke, 2022b*	40	53	0.755	[0.617; 0.862]
Meilke, 2022c*	214	326	0.656	[0.602; 0.708]
Mendes, 2024a*	80	92	0.870	[0.783; 0.931]
Mendes, 2024c*	104	111	0.937	[0.874; 0.974]
Niimi, 2024c	136	202	0.673	[0.604; 0.737]
Ossenkoppele, 2021a	200	256	0.781	[0.726; 0.830]
Palmqvist, 2020a*	300	397	0.756	[0.710; 0.797]
Palmqvist, 2020d*	184	274	0.672	[0.612; 0.727]
Thanapornsangsuth, 2024	62	71	0.873	[0.773; 0.940]
Thijssen, 2021d*	56	65	0.862	[0.753; 0.935]
Thijssen, 2021e*	44	46	0.957	[0.852; 0.995]
VandeVrede, 2023a*	152	192	0.792	[0.727; 0.847]
VandeVrede, 2023b*	124	152	0.816	[0.745; 0.874]
Random effects model		4791	0.780	[0.714; 0.834]
Heterogeneity: $I^2 = 94\%$, $\tau^2 = 0.7813$, $p < 0.01$				
**g = Plasma, Simoa_ALZpath**				
Figdore, 2024a	460	528	0.871	[0.840; 0.899]
Therriault, 2024a	160	175	0.914	[0.863; 0.951]
Meyer, 2024	18	38	0.474	[0.310; 0.642]
Therriault, 2024b	46	79	0.582	[0.466; 0.692]
Ashton, 2024 a*	92	125	0.736	[0.650; 0.811]
Ashton, 2024 b*	88	108	0.815	[0.729; 0.883]
Ashton, 2024 c*	92	110	0.836	[0.754; 0.900]
Ashton, 2024d	98	143	0.685	[0.602; 0.760]
Asken, 2024	160	196	0.816	[0.755; 0.868]
Therriault, 2024c	198	232	0.853	[0.801; 0.896]
Xiao, 2023	18	27	0.667	[0.460; 0.835]
Random effects model		1761	0.775	[0.692; 0.840]
Heterogeneity: $I^2 = 89\%$, $\tau^2 = 0.4596$, $p < 0.01$				
**g = Plasma, Simoa_Janssen R&D**				
Dore, 2022j	154	195	0.790	[0.726; 0.845]
Dore, 2022o*	206	238	0.866	[0.816; 0.906]
Feizpour, 2024c	218	249	0.876	[0.828; 0.914]
Therriault, 2024d	150	169	0.888	[0.830; 0.931]
Dore, 2022b*	54	109	0.495	[0.398; 0.593]
Dore, 2022e*	70	115	0.609	[0.513; 0.698]
Therriault, 2024e	48	78	0.615	[0.498; 0.723]
Dore, 2022i*	222	301	0.738	[0.684; 0.786]
Dore, 2022p*	256	326	0.785	[0.737; 0.829]
Feizpour, 2024a	140	181	0.773	[0.706; 0.832]
Therriault, 2022a*	112	136	0.824	[0.749; 0.884]
Therriault, 2024f*	196	234	0.838	[0.784; 0.882]
Woo, 2023a*	160	182	0.879	[0.823; 0.923]
Woo, 2023b*	80	137	0.584	[0.497; 0.667]
Random effects model		2650	0.772	[0.704; 0.829]
Heterogeneity: $I^2 = 92\%$, $\tau^2 = 0.4203$, $p < 0.01$				
**Random effects model**		**14445**	**0.783**	**[0.750; 0.813]**
Heterogeneity: $I^2 = 94\%$, $\tau^2 = 0.5722$, $p < 0.01$				
Test for subgroup differences: $\chi^2_7 = 88.59$, df = 7 ($p < 0.01$)				

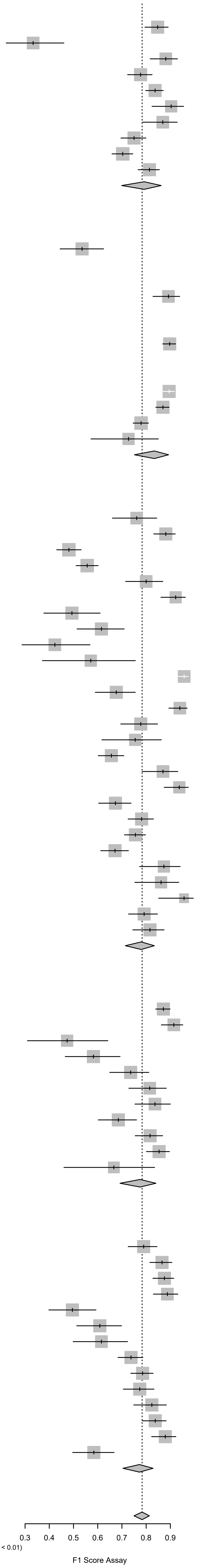

Supplement: Supplementary file 4 — Supporting Information [file ALZ-21-e14458-s009.pdf]
