# Supplementary material for: Diagnostic accuracy of phosphorylated tau217 in detecting Alzheimer's disease pathology among cognitively impaired and unimpaired: A systematic review and meta‐analysis
Source: Alzheimers Dement. 2024 Dec 23;21(2):e14458. doi: 10.1002/alz.14458 (PMC11848338; doi:10.1002/alz.14458)

Supplemental Figure-5 Forest plots: demonstrating the diagnostic performance of Lilly, Simoa-ALZpath, Simoa-Jansson R&D, and Mass Spectrometry for phosphorylated tau-217 (p-Tau217), stratified by different cognitive groups—Cognitively Unimpaired (CU), combined Cognitively Unimpaired and Cognitively Impaired (CU+CI), and Cognitively Impaired (CI). g: group of studies that used cerebrospinal fluid (CSF) or plasma biomarkers; I^2: the proportion of variation due to heterogeneity; τ^2: between-study variance in a random-effects model. χ^2_x: chi-square test statistic with x degrees of freedom (df = x).

Panel (A) depicts the sensitivity in each group, reflecting how well the platforms identified true positive cases within each cognitive category. Events: number of subjects testing positive; Total: actual total number of positive subjects in the cohort; Proportion: proportion of positive cases, detected by the assay (sensitivity). Panel (B) illustrates the specificity, indicating their ability to correctly identify true negatives across these groups. Events: number of subjects testing negative; Total: actual total number of negative subjects in the cohort; Proportion: proportion of negative cases, detected by the assay (specificity). Panel (C) shows the diagnostic odds ratio (OR), combining sensitivity and specificity to assess the overall diagnostic effectiveness of each platform within the CU, CU+CI, and CI groups. Events: number of true positive cases identified by the test; Total: actual number of positive cases in the cohort; Proportion: expressed as the DOR, provides the odds of achieving a true positive result versus a false positive. Panel (D) provides the F1 score, offering a balanced view of test performance by calculating the harmonic mean of sensitivity and precision, ensuring a comprehensive evaluation of the platforms' ability to detect AD pathology within each cognitive group. Events: number of cases where precision and recall both resulted in a truly positive outcome; Total: total number of positive predictions made by the test; Proportion: balance between sensitivity/recall and precision, giving a single measure that accounts for both false positives and false negatives.

Supp, Figure-5A

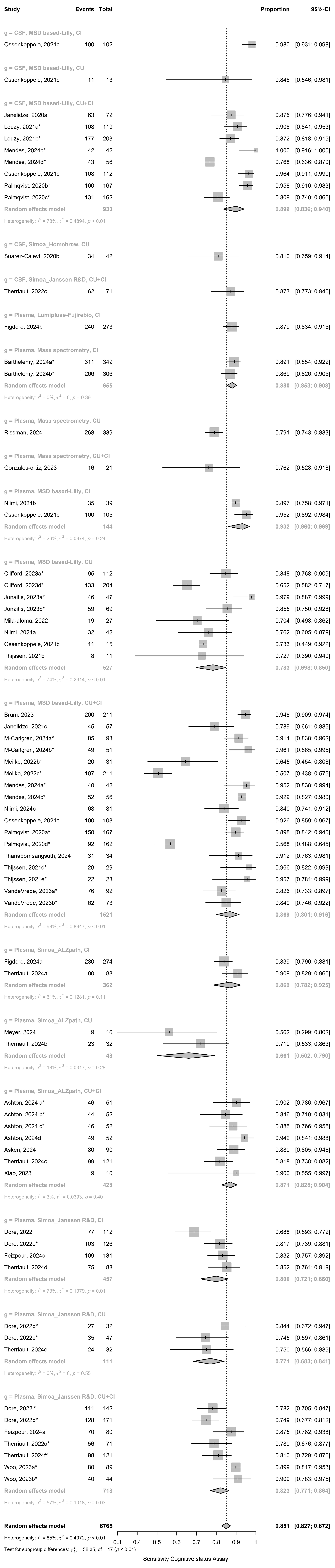

Supp, Figure-5B

Study	Events	Total	Proportion	95%-CI
**g = CSF, MSD based-Lilly, CI**				
Ossenkoppele, 2021c	45	79	0.570	[0.453; 0.681]
**g = CSF, MSD based-Lilly, CU**				
Ossenkoppele, 2021e	164	206	0.796	[0.735; 0.849]
**g = CSF, MSD based-Lilly, CU+CI**				
Janelidze, 2020a	57	65	0.877	[0.772; 0.945]
Leuzy, 2021a*	459	510	0.900	[0.871; 0.925]
Leuzy, 2021b*	383	426	0.899	[0.866; 0.926]
Mendes, 2024b*	63	72	0.875	[0.776; 0.941]
Mendes, 2024d*	45	45	1.000	[0.921; 1.000]
Ossenkoppele, 2021d	220	288	0.764	[0.711; 0.812]
Palmqvist, 2020b*	404	532	0.759	[0.721; 0.795]
Palmqvist, 2020c*	297	326	0.911	[0.875; 0.940]
**Random effects model**		**2264**	**0.868**	**[0.814; 0.908]**
Heterogeneity: $I^2 = 91\%$, $\tau^2 = 0.2576$, $p < 0.01$				
**g = CSF, Simoa_Homebrew, CU**				
Suarez-Calevt, 2020b	236	287	0.822	[0.773; 0.865]
**g = CSF, Simoa_Janssen R&D, CU+CI**				
Therriault, 2022c	97	103	0.942	[0.878; 0.978]
**g = Plasma, Lumipluse-Fujirebio, CI**				
Figdore, 2024b	132	154	0.857	[0.792; 0.908]
**g = Plasma, Mass spectrometry, CI**				
Barthelemy, 2024a*	318	353	0.901	[0.865; 0.930]
Barthelemy, 2024b*	365	405	0.901	[0.868; 0.928]
**Random effects model**		**758**	**0.901**	**[0.878; 0.920]**
Heterogeneity: $I^2 = 0\%$, $\tau^2 = 0$, $p = 0.99$				
**g = Plasma, Mass spectrometry, CU**				
Rissman, 2024	659	740	0.891	[0.866; 0.912]
**g = Plasma, Mass spectrometry, CU+CI**				
Gonzales-ortiz, 2023	65	72	0.903	[0.810; 0.960]
**g = Plasma, MSD based-Lilly, CI**				
Niimi, 2024b	86	104	0.827	[0.740; 0.894]
Ossenkoppele, 2021c	54	76	0.711	[0.595; 0.809]
**Random effects model**		**180**	**0.774**	**[0.640; 0.868]**
Heterogeneity: $I^2 = 70\%$, $\tau^2 = 0.1562$, $p = 0.07$				
**g = Plasma, MSD based-Lilly, CU**				
Clifford, 2023a*	564	752	0.750	[0.717; 0.781]
Clifford, 2023d*	423	564	0.750	[0.712; 0.785]
Jonaitis, 2023a*	89	111	0.802	[0.715; 0.871]
Jonaitis, 2023b*	95	95	1.000	[0.962; 1.000]
Mila-aloma, 2022	281	312	0.901	[0.862; 0.931]
Niimi, 2024a	260	290	0.897	[0.856; 0.929]
Ossenkoppele, 2021b	177	203	0.872	[0.818; 0.915]
Thijssen, 2021b	44	53	0.830	[0.702; 0.919]
**Random effects model**		**2380**	**0.844**	**[0.785; 0.889]**
Heterogeneity: $I^2 = 90\%$, $\tau^2 = 0.2414$, $p < 0.01$				
**g = Plasma, MSD based-Lilly, CU+CI**				
Brum, 2023	130	137	0.949	[0.898; 0.979]
Janelidze, 2021c	117	148	0.791	[0.716; 0.853]
M-Carlgren, 2024a*	22	25	0.880	[0.688; 0.975]
M-Carlgren, 2024b*	68	94	0.723	[0.622; 0.811]
Meilke, 2022b*	16	18	0.889	[0.653; 0.986]
Meilke, 2022c*	195	203	0.961	[0.924; 0.983]
Mendes, 2024a*	62	72	0.861	[0.759; 0.931]
Mendes, 2024c*	42	45	0.933	[0.817; 0.986]
Niimi, 2024c	340	393	0.865	[0.827; 0.897]
Ossenkoppele, 2021a	244	292	0.836	[0.788; 0.876]
Palmqvist, 2020a*	452	532	0.850	[0.816; 0.879]
Palmqvist, 2020d*	306	326	0.939	[0.907; 0.962]
Thanapornsangsuth, 2024	28	34	0.824	[0.655; 0.932]
Thijssen, 2021d*	36	44	0.818	[0.673; 0.918]
Thijssen, 2021e*	22	23	0.957	[0.781; 0.999]
VandeVrede, 2023a*	191	215	0.888	[0.838; 0.927]
VandeVrede, 2023b*	137	154	0.890	[0.829; 0.934]
**Random effects model**		**2755**	**0.879**	**[0.843; 0.907]**
Heterogeneity: $I^2 = 76\%$, $\tau^2 = 0.2743$, $p < 0.01$				
**g = Plasma, Simoa_ALZpath, CI**				
Figdore, 2024a	129	153	0.843	[0.776; 0.897]
Therriault, 2024a	35	42	0.833	[0.686; 0.930]
**Random effects model**		**195**	**0.841**	**[0.783; 0.886]**
Heterogeneity: $I^2 = 0\%$, $\tau^2 = 0$, $p = 0.88$				
**g = Plasma, Simoa_ALZpath, CU**				
Meyer, 2024	46	59	0.780	[0.653; 0.877]
Therriault, 2024b	83	107	0.776	[0.685; 0.851]
**Random effects model**		**166**	**0.777**	**[0.708; 0.834]**
Heterogeneity: $I^2 = 0\%$, $\tau^2 = 0$, $p = 0.95$				
**g = Plasma, Simoa_ALZpath, CU+CI**				
Ashton, 2024 a*	181	209	0.866	[0.812; 0.909]
Ashton, 2024 b*	68	80	0.850	[0.753; 0.920]
Ashton, 2024 c*	68	80	0.850	[0.753; 0.920]
Ashton, 2024d	155	197	0.787	[0.723; 0.842]
Asken, 2024	123	149	0.826	[0.755; 0.883]
Therriault, 2024c	136	148	0.919	[0.863; 0.957]
Xiao, 2023	52	60	0.867	[0.754; 0.941]
**Random effects model**		**923**	**0.851**	**[0.813; 0.882]**
Heterogeneity: $I^2 = 51\%$, $\tau^2 = 0.0662$, $p = 0.06$				
**g = Plasma, Simoa_Janssen R&D, CI**				
Dore, 2022j	56	62	0.903	[0.801; 0.964]
Dore, 2022o*	39	48	0.812	[0.674; 0.911]
Feizpour, 2024c	53	62	0.855	[0.742; 0.931]
Therriault, 2024d	36	42	0.857	[0.715; 0.946]
**Random effects model**		**214**	**0.857**	**[0.802; 0.898]**
Heterogeneity: $I^2 = 0\%$, $\tau^2 = 0$, $p = 0.61$				
**g = Plasma, Simoa_Janssen R&D, CU**				
Dore, 2022b*	141	191	0.738	[0.670; 0.799]
Dore, 2022e*	143	176	0.812	[0.747; 0.867]
Therriault, 2024e	85	107	0.794	[0.705; 0.866]
**Random effects model**		**474**	**0.779**	**[0.727; 0.824]**
Heterogeneity: $I^2 = 36\%$, $\tau^2 = 0.0243$, $p = 0.21$				
**g = Plasma, Simoa_Janssen R&D, CU+CI**				
Dore, 2022i*	206	254	0.811	[0.757; 0.857]
Dore, 2022p*	199	226	0.881	[0.831; 0.920]
Feizpour, 2024a	161	192	0.839	[0.779; 0.888]
Therriault, 2022a*	91	100	0.910	[0.836; 0.958]
Therriault, 2024f*	133	148	0.899	[0.838; 0.942]
Woo, 2023a*	132	145	0.910	[0.852; 0.951]
Woo, 2023b*	137	190	0.721	[0.652; 0.784]
**Random effects model**		**1255**	**0.857**	**[0.803; 0.898]**
Heterogeneity: $I^2 = 83\%$, $\tau^2 = 0.2179$, $p < 0.01$				
**Random effects model**		**13205**	**0.853**	**[0.837; 0.869]**

Heterogeneity: $I^2 = 84\%$, $\tau^2 = 0.2161$, $p < 0.01$

Test for subgroup differences: $\chi^2_{17} = 102.84$, df = 17 ($p < 0.01$)

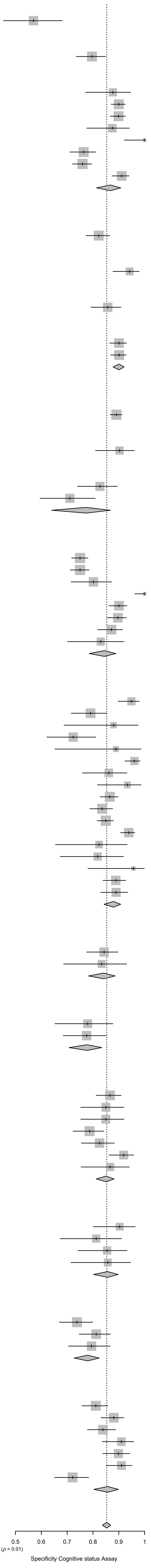

Supp, Figure-5C

Study	Experimental Events	Experimental Total	Control Events	Control Total	Odds Ratio	OR	95%-CI
**g = CSF, MSD based-Lilly, CI**							
Ossenkoppele, 2021c	100	134	2	47		66.17647	[15.23330; 287.48364]
**g = CSF, MSD based-Lilly, CU**							
Ossenkoppele, 2021e	11	53	2	166		21.47619	[4.58437; 100.60858]
**g = CSF, MSD based-Lilly, CU+CI**							
Janelidze, 2020a	63	71	9	66		49.87500	[18.02903; 137.97278]
Leuzy, 2021a*	108	159	11	470		88.36364	[44.56734; 175.19853]
Leuzy, 2021b*	177	220	26	409		60.63596	[36.10429; 101.83608]
Mendes, 2024b*	42	51	0	63		568.15789	[32.20685; 10022.81863]
Mendes, 2024d*	43	43	13	58		293.22222	[16.90866; 5084.92519]
Ossenkoppele, 2021d	108	176	4	224		87.35294	[31.05014; 245.74886]
Palmqvist, 2020b*	160	288	7	411		72.14286	[32.98809; 157.77185]
Palmqvist, 2020c*	131	160	31	328		43.27809	[25.05733; 74.74830]
Random effects model		1168		2029		63.38764	[48.02452; 83.66545]
Heterogeneity: $I^2 = 0\%$, $\tau^2 = < 0.0001$, $p = 0.45$							
**g = CSF, Simoa_Homebrew, CU**							
Suarez-Calevt, 2020b	34	85	8	244		19.66667	[8.59705; 44.98960]
**g = CSF, Simoa_Janssen R&D, CU+CI**							
Therriault, 2022c	62	68	9	106		111.37037	[37.78181; 328.28919]
**g = Plasma, Lumipluse-Fujirebio, CI**							
Figdore, 2024b	240	262	33	165		43.63636	[24.43767; 77.91792]
**g = Plasma, Mass spectrometry, CI**							
Barthelemy, 2024a*	311	346	38	356		74.35940	[45.78032; 120.77940]
Barthelemy, 2024b*	266	306	40	405		60.68125	[38.08271; 96.68993]
Random effects model		652		761		66.89832	[47.80711; 93.61338]
Heterogeneity: $I^2 = 0\%$, $\tau^2 = 0$, $p = 0.55$							
**g = Plasma, Mass spectrometry, CU**							
Rissman, 2024	268	349	71	730		30.70979	[21.66581; 43.52900]
**g = Plasma, Mass spectrometry, CU+CI**							
Gonzales-ortiz, 2023	16	23	5	70		29.71429	[8.33368; 105.94827]
**g = Plasma, MSD based-Lilly, CI**							
Niimi, 2024b	35	53	4	90		41.80556	[13.20443; 132.35745]
Ossenkoppele, 2021c	100	122	5	59		49.09091	[17.59792; 136.94327]
Random effects model		175		149		45.72545	[21.25046; 98.38928]
Heterogeneity: $I^2 = 0\%$, $\tau^2 = 0$, $p = 0.84$							
**g = Plasma, MSD based-Lilly, CU**							
Clifford, 2023a*	95	283	17	581		16.76471	[9.75112; 28.82289]
Clifford, 2023d*	133	274	71	494		5.61972	[3.97833; 7.93831]
Jonaitis, 2023a*	46	68	1	90		186.09091	[24.30938; 1424.54573]
Jonaitis, 2023b*	59	59	10	105		1082.33333	[62.26696; 18813.27541]
Mila-aloma, 2022	19	50	8	289		21.52823	[8.70461; 53.24360]
Niimi, 2024a	32	62	10	270		27.73333	[12.40702; 61.99213]
Ossenkoppele, 2021b	11	37	4	181		18.72115	[5.54833; 63.16889]
Thijssen, 2021b	8	17	3	47		13.03704	[2.88507; 58.91168]
Random effects model		850		2057		23.26666	[10.59316; 51.10257]
Heterogeneity: $I^2 = 83\%$, $\tau^2 = 0.9022$, $p < 0.01$							
**g = Plasma, MSD based-Lilly, CU+CI**							
Brum, 2023	200	207	11	141		337.66234	[127.61658; 893.42510]
Janelidze, 2021c	45	76	12	129		14.15323	[6.68668; 29.95715]
M-Carlgren, 2024a*	85	88	8	30		77.91667	[19.07439; 318.28049]
M-Carlgren, 2024b*	49	75	2	70		64.07692	[14.52266; 282.72044]
Meilke, 2022b*	20	22	11	27		14.54545	[2.81079; 75.27065]
Meilke, 2022c*	107	115	104	299		25.07812	[11.76599; 53.45174]
Mendes, 2024a*	40	50	2	64		124.00000	[25.81428; 595.63924]
Mendes, 2024c*	52	55	4	46		182.00000	[38.58321; 858.50806]
Niimi, 2024c	68	121	13	353		33.55588	[17.34120; 64.93190]
Ossenkoppele, 2021a	100	148	8	252		63.54167	[29.01688; 139.14466]
Palmqvist, 2020a*	150	230	17	469		49.85294	[28.61739; 86.84635]
Palmqvist, 2020d*	92	112	70	376		20.10857	[11.61474; 34.81393]
Thanapornsangsuth, 2024	31	37	3	31		48.22222	[11.00901; 211.22545]
Thijssen, 2021d*	28	36	1	37		126.00000	[14.87332; 1067.41500]
Thijssen, 2021e*	22	23	1	23		484.00000	[28.44526; 8235.32532]
VandeVrede, 2023a*	76	100	16	207		37.80208	[19.03365; 75.07743]
VandeVrede, 2023b*	62	79	11	148		45.42246	[20.09387; 102.67810]
Random effects model		1574		2702		50.10529	[32.55930; 77.10669]
Heterogeneity: $I^2 = 67\%$, $\tau^2 = 0.4953$, $p < 0.01$							
**g = Plasma, Simoa_ALZpath, CI**							
Figdore, 2024a	230	254	44	173		28.09659	[16.33927; 48.31418]
Therriault, 2024a	80	87	8	43		50.00000	[16.82135; 148.62068]
Random effects model		341		216		31.50165	[19.38941; 51.18018]
Heterogeneity: $I^2 = 0\%$, $\tau^2 = 0$, $p = 0.35$							
**g = Plasma, Simoa_ALZpath, CU**							
Meyer, 2024	9	22	7	53		4.54945	[1.42067; 14.56885]
Therriault, 2024b	23	47	9	92		8.83796	[3.61294; 21.61940]
Random effects model		69		145		6.90648	[3.39807; 14.03722]
Heterogeneity: $I^2 = 0\%$, $\tau^2 = 0$, $p = 0.38$							
**g = Plasma, Simoa_ALZpath, CU+CI**							
Ashton, 2024 a*	46	74	5	186		59.47143	[21.76708; 162.48621]
Ashton, 2024 b*	44	56	8	76		31.16667	[11.79529; 82.35162]
Ashton, 2024 c*	46	58	6	74		43.44444	[15.21831; 124.02295]
Ashton, 2024d	49	91	3	158		60.27778	[17.89308; 203.06232]
Asken, 2024	80	106	10	133		37.84615	[17.31845; 82.70552]
Therriault, 2024c	99	111	22	158		51.00000	[24.10223; 107.91531]
Xiao, 2023	9	17	1	53		58.50000	[6.50807; 525.84705]
Random effects model		513		838		45.29255	[31.24922; 65.64693]
Heterogeneity: $I^2 = 0\%$, $\tau^2 = 0$, $p = 0.96$							
**g = Plasma, Simoa_Janssen R&D, CI**							
Dore, 2022j	77	83	35	91		20.53333	[8.08592; 52.14224]
Dore, 2022o*	103	112	23	62		19.40580	[8.25963; 45.59342]
Feizpour, 2024c	109	118	22	75		29.17677	[12.56926; 67.72743]
Therriault, 2024d	75	81	13	49		34.61538	[12.16439; 98.50264]
Random effects model		394		277		24.70064	[15.68313; 38.90306]
Heterogeneity: $I^2 = 0\%$, $\tau^2 = 0$, $p = 0.80$							
**g = Plasma, Simoa_Janssen R&D, CU**							
Dore, 2022b*	27	77	5	146		15.22800	[5.56135; 41.69704]
Dore, 2022e*	35	68	12	155		12.63889	[5.92815; 26.94626]
Therriault, 2024e	24	46	8	93		11.59091	[4.58520; 29.30063]
Random effects model		191		394		12.91133	[7.77788; 21.43290]
Heterogeneity: $I^2 = 0\%$, $\tau^2 = 0$, $p = 0.92$							
**g = Plasma, Simoa_Janssen R&D, CU+CI**							
Dore, 2022i*	111	159	31	237		15.36694	[9.25409; 25.51767]
Dore, 2022p*	128	155	43	242		21.93971	[12.91348; 37.27505]
Feizpour, 2024a	70	101	10	171		36.35484	[16.89973; 78.20685]
Therriault, 2022a*	56	65	15	106		37.74815	[15.48725; 92.00618]
Therriault, 2024f*	98	113	23	156		37.77971	[18.74480; 76.14413]
Woo, 2023a*	80	93	9	141		90.25641	[36.91202; 220.69286]
Woo, 2023b*	40	93	4	141		25.84906	[8.81691; 75.78322]
Random effects model		779		1194		30.73394	[20.09621; 47.00267]
Heterogeneity: $I^2 = 58\%$, $\tau^2 = 0.1831$, $p = 0.03$							
**Random effects model**		**7680**		**12290**		**36.43330**	**[30.07743; 44.13226]**

Heterogeneity: $I^2 = 76\%$, $\tau^2 = 0.4035$, $p < 0.01$

Test for subgroup differences: $\chi^2_{17} = 81.78$, df = 17 ($p < 0.01$)

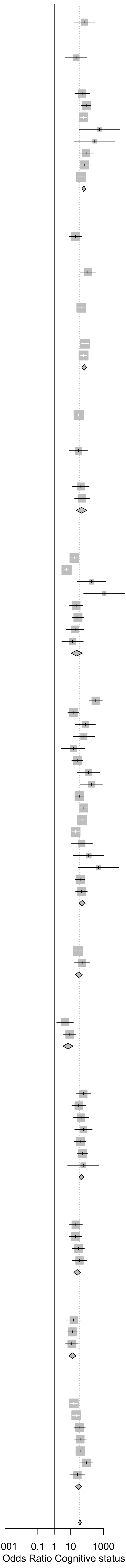

Supp, Figure-5D

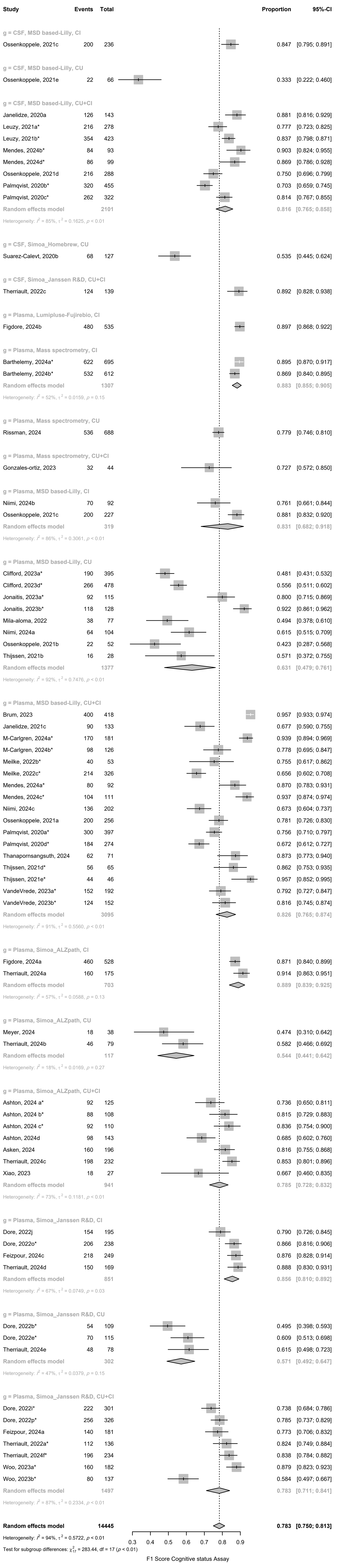

Supplement: Supplementary file 5 — Supporting Information [file ALZ-21-e14458-s003.pdf]
