# Supplementary material for: Diagnostic accuracy of phosphorylated tau217 in detecting Alzheimer's disease pathology among cognitively impaired and unimpaired: A systematic review and meta‐analysis
Source: Alzheimers Dement. 2024 Dec 23;21(2):e14458. doi: 10.1002/alz.14458 (PMC11848338; doi:10.1002/alz.14458)
Supplement: Supplementary file 6 — Supporting Information [file ALZ-21-e14458-s006.pdf]

Supplemental Figure-6 Funnel plot showing the assessment of publication bias for the studies included in the analysis. The x-axis represents the log risk ratio, while the y-axis shows the standard error, indicating the precision of each study's effect size. Each dot represents an individual study's effect size and its corresponding standard error.

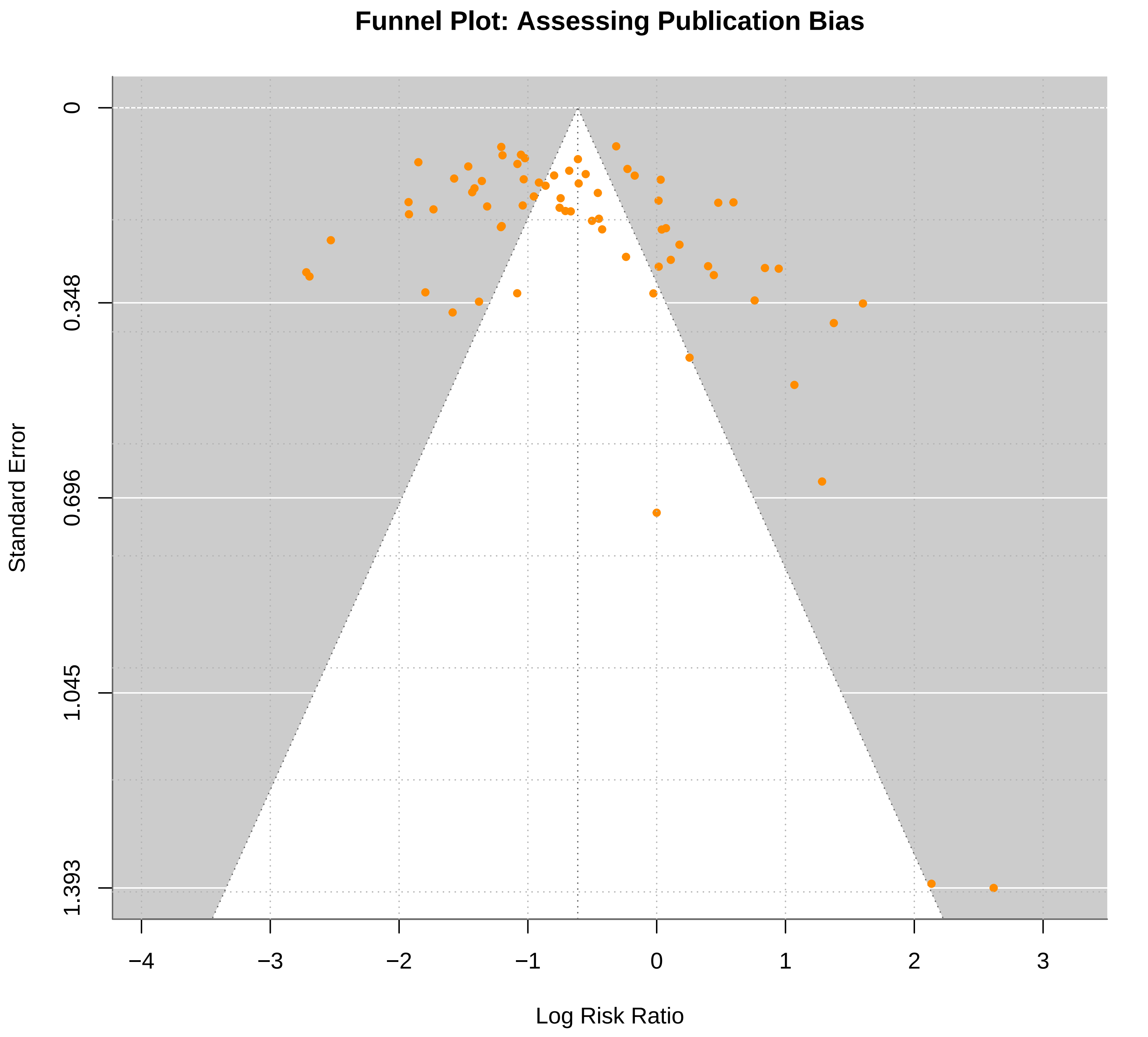

Funnel Plot: Assessing Publication Bias
Standard Error
0
0.348
0.696
1.045
1.393
−4
−3
−2
−1
0
1
2
3
Log Risk Ratio
