# Supplementary material for: Diagnostic accuracy of phosphorylated tau217 in detecting Alzheimer's disease pathology among cognitively impaired and unimpaired: A systematic review and meta‐analysis
Source: Alzheimers Dement. 2024 Dec 23;21(2):e14458. doi: 10.1002/alz.14458 (PMC11848338; doi:10.1002/alz.14458)
Supplement: Supplementary file 7 — Supporting Information [file ALZ-21-e14458-s008.pdf]

Supplemental Figure-7 The violin plots display p-Tau217 F1 scores of the included studies, including A) Plasma p-Tau217 F1 scores across different cognitive groups of Cognitively Unimpaired, cognitively Impaired, and Mixed Cognitively Unimpaired and Impaired in predicting amyloid and tau PET positivity. B) CSF p-Tau217 F1 scores across different cognitive groups of Cognitively Unimpaired, cognitively impaired, and Mixed Cognitively Unimpaired and Impaired in predicting amyloid and tau PET positivity.

*Note: An F1 score of 0.7 or higher is considered good. 0.8-0.9 is great. 0.98-0.99 is excellent. Scores below 0.5 are considered poor.

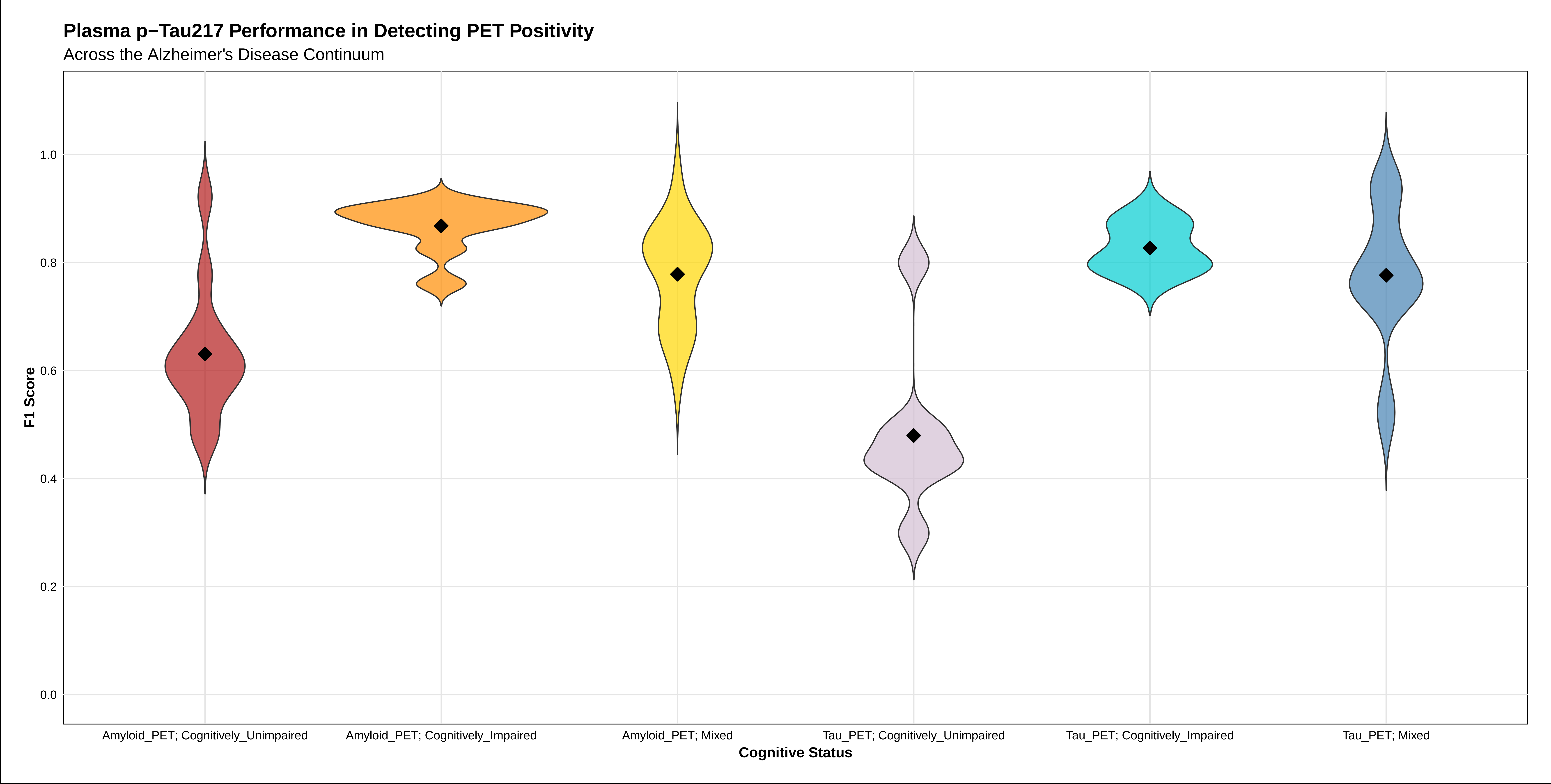
Plasma p−Tau217 Performance in Detecting PET Positivity
Across the Alzheimer's Disease Continuum
F1 Score
0.0
0.2
0.4
0.6
0.8
1.0
Amyloid_PET; Cognitively_Unimpaired
Amyloid_PET; Cognitively_Impaired
Amyloid_PET; Mixed
Tau_PET; Cognitively_Unimpaired
Tau_PET; Cognitively_Impaired
Tau_PET; Mixed
Cognitive Status

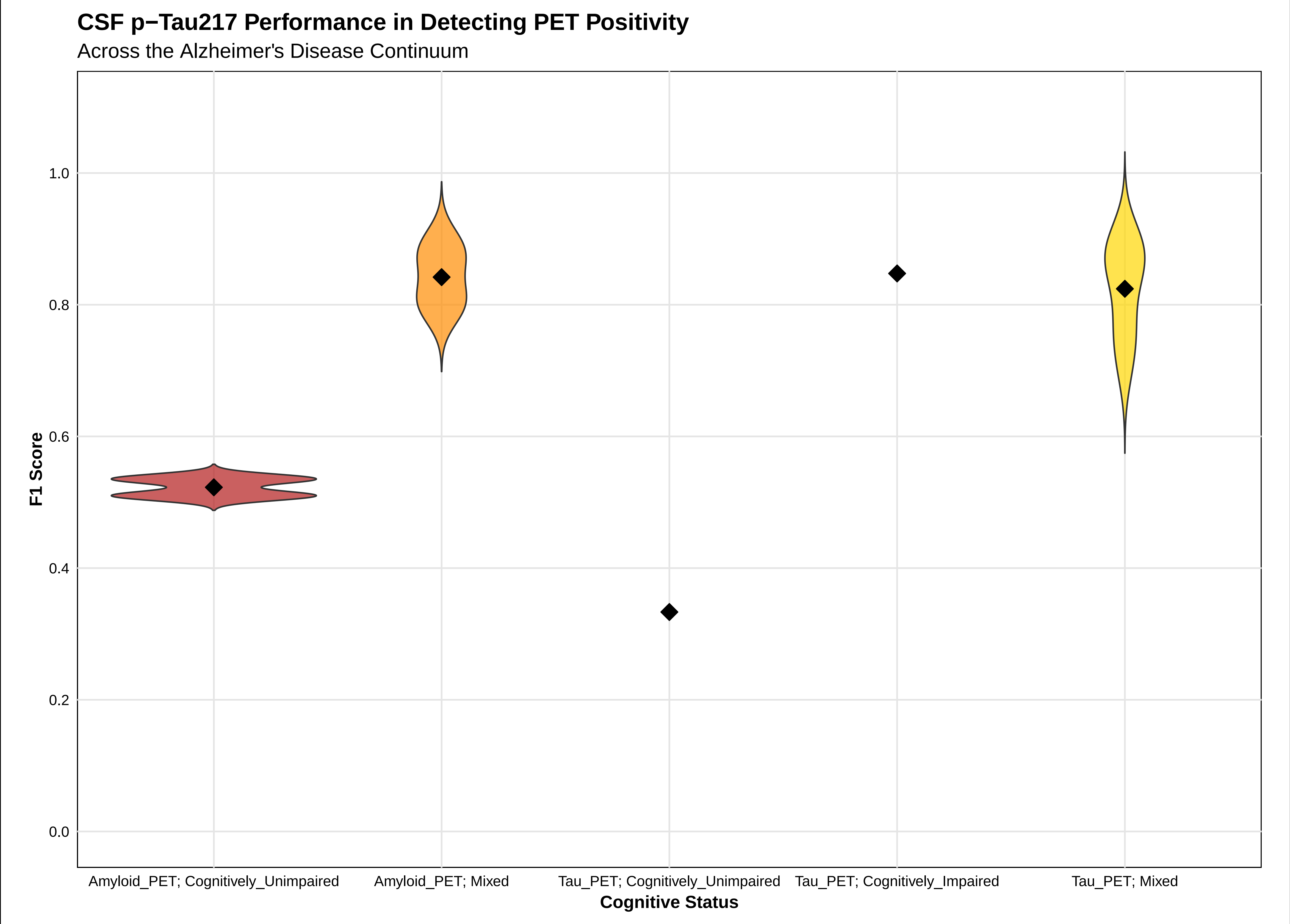

CSF p−Tau217 Performance in Detecting PET Positivity
Across the Alzheimer's Disease Continuum
F1 Score
1.0
0.8
0.6
0.4
0.2
0.0
Amyloid_PET; Cognitively_Unimpaired
Amyloid_PET; Mixed
Tau_PET; Cognitively_Unimpaired
Tau_PET; Cognitively_Impaired
Tau_PET; Mixed
Cognitive Status
