# Supplementary material for: Diagnostic accuracy of phosphorylated tau217 in detecting Alzheimer's disease pathology among cognitively impaired and unimpaired: A systematic review and meta‐analysis
Source: Alzheimers Dement. 2024 Dec 23;21(2):e14458. doi: 10.1002/alz.14458 (PMC11848338; doi:10.1002/alz.14458)
Supplement: Supplementary file 8 — Supporting Information [file ALZ-21-e14458-s004.docx]

**Supplemental Table-1** QUADAS-2 results ( = Low risk probability, = Not clear risk, = High risk probability)

| **Study** | **RISK OF BIAS** | | | | **APPLICABILITY CONCERNS** | | |  |
| --- | --- | --- | --- | --- | --- | --- | --- | --- |
|  | **PATIENT SELECTION** | **INDEX TEST** | **REFERENCE STANDARD** | **FLOW AND TIMING** | **PATIENT SELECTION** | **INDEX TEST** | **REFERENCE STANDARD** | **Interpretation** |
| Palmqvist et al, 2020 |  |  |  |  |  |  |  | Low overall risk of bias |
| Janelidze et al, 2020 |  |  |  |  |  |  |  | Low overall risk of bias |
| Janelidze et al, 2021 |  |  |  |  |  |  |  | Low overall risk of bias |
| Leuzy et al, 2021 |  |  |  |  |  |  |  | Low overall risk of bias |
| Thijssen et al, 2021 |  |  |  |  |  |  |  | Low overall risk of bias |
| Ossenkoppele et al, 2021 |  |  |  |  |  |  |  | Low overall risk of bias |
| Therriault et al, 2022 |  |  |  |  |  |  |  | High overall risk of bias |
| Dore et al, 2022 |  |  |  |  |  |  |  | Low overall risk of bias |
| Mila-aloma et al, 2022 |  |  |  |  |  |  |  | Low overall risk of bias |
| Meilke et al, 2022 |  |  |  |  |  |  |  | Low overall risk of bias |
| Brum et al, 2023 |  |  |  |  |  |  |  | High overall risk of bias |
| Woo et al, 2023 |  |  |  |  |  |  |  | High overall risk of bias |
| VandeVrede et al, 2023 |  |  |  |  |  |  |  | Low overall risk of bias |
| Xiao et al, 2023 |  |  |  |  |  |  |  | Low overall risk of bias |
| Jonaitis et al, 2023 |  |  |  |  |  |  |  | Low overall risk of bias |
| Rissman et al, 2023 |  |  |  |  |  |  |  | Low overall risk of bias |
| Gonzales-ortiz et al, 2023 |  |  |  |  |  |  |  | Low overall risk of bias |
| Clifford et al, 2023 |  |  |  |  |  |  |  | Low overall risk of bias |
| M-Carlgren et al, 2024 |  |  |  |  |  |  |  | Low overall risk of bias |
| Mendes et al, 2024 |  |  |  |  |  |  |  | Moderate overall risk of bias |
| Feizpour, 2024 |  |  |  |  |  |  |  | Low overall risk of bias |
| Ashton et al, 2024 |  |  |  |  |  |  |  | Low overall risk of bias |
| Barthelemy et al, 2024 |  |  |  |  |  |  |  | Low overall risk of bias |
| Therriault et al, 2024 |  |  |  |  |  |  |  | Low overall risk of bias |
| Niimi et al, 2024 |  |  |  |  |  |  |  | Moderate overall risk of bias |
| Thanapornsangsuth et al, 2024 |  |  |  |  |  |  |  | Low overall risk of bias |
| Asken et al, 2024 |  |  |  |  |  |  |  | Low overall risk of bias |
| Figdore et al, 2024 |  |  |  |  |  |  |  | Low overall risk of bias |
| De Meyer et al, 2024 |  |  |  |  |  |  |  | Low overall risk of bias |
