# Supplementary material for: Diagnostic accuracy of phosphorylated tau217 in detecting Alzheimer's disease pathology among cognitively impaired and unimpaired: A systematic review and meta‐analysis
Source: Alzheimers Dement. 2024 Dec 23;21(2):e14458. doi: 10.1002/alz.14458 (PMC11848338; doi:10.1002/alz.14458)
Supplement: Supplementary file 9 — Supporting Information [file ALZ-21-e14458-s007.docx]

**Supplemental Table-2** Descriptive information from the excluded studies; these studies were excluded from the analysis due to insufficient reporting of diagnostic test accuracy (DTA) data or details related to PET imaging, such as incomplete or missing information on sensitivity and specificity. In addition, studies were excluded if data from the same cohort were already included in this meta-analysis.

| **ID** | **Cohort location** | **Source** | **Assay** | **Participants (AD/MCI/CN)** | **Age**  **(AD/MCI/CN)** | **Tracer** | **Finding(s)** | **Limitation(s)** | **Comments** |
| --- | --- | --- | --- | --- | --- | --- | --- | --- | --- |
| **Mattsson-Carlgren2020 ^1^** | Sweden | CSF | Lilly | (35/38/58) | (72/71.8/74.5) | [18F]Flortaucipir | *CSF levels of tau proteins, especially p-Tau217 and p-Tau181, are increased in response to Aβ deposition in humans in the early stages of AD. | *Lack of neuropathology  *Small sample size.  * Clinic-based cohort.  * The possible difference in each technique's sensitivity. | * Lack of sufficient DTA data.  * Data from this cohort have already been included in another study. |
| **Barthelemy, 2020 ^2^** | USA | Plasma, CSF | Mass spectrometry | (2/8/8) | (75/75.6/74) | [11C]PIB, [18F]AV1451 | *Plasma p-Tau, especially p-tau-217, mirrors particular modifications in CSF to detect phosphorylation changes in soluble tau and amyloidosis. | *Small sample size.  * Clinic-based cohort. | * Lack of sufficient DTA data.  * Data from this cohort have already been included in another study. |
| **Barthelemy, 2020-2 ^3^** | USA | CSF | Mass spectrometry | (AB+=33, AB-=51) | (AB+=67, AB-=62.7) | [11C]PIB | *p-Tau217 is a more accurate biomarker than p-Tau181, which could be used to improve the diagnosis and follow-up of preclinical to advanced cases of Alzheimer’s disease (AD). | *Small sample size.  * Clinic-based cohort. | * Lack of sufficient DTA data.  * Data from this cohort have already been included in another study. |
| Brickman, 2021 ^4^ | USA | Plasma | Simoa | (AB+=32, AB-=8) | (AB+=82.6, AB-=84.3) | [18F]Florbetaben | * p-tau217 is a useful indicator for evaluating biomarkers and understanding demographics in community-based studies. | *Small sample size. | * Lack of sufficient DTA data. |
| **Lantero-Rodriguez, 2021 ^5^** | Sweden | CSF | Mass spectrometry | (20/20/82) | (64.1/72.2/71.4) | [18F]MK-6240, [18F]AZD4694,  [18F]flutemetamol | * CSF p-Tau217 captures early AD changes better than CSF p-tau235, while both have similar accuracy in distinguishing CI and CI+. | * Clinic-based cohort.  *Small sample size.  *A cross-sectional analysis. | * Lack of sufficient DTA data.  * Data from this cohort have already been included in another study. |
| **Janelidze, 2021 ^6^** | Sweden | Plasma | Lilly | (NA/164/350) | (NA/71/64) | [18F]flutemetamol | * Plasma p-tau217 is the most predictive biomarker for Aβ status in MCI patients, aiding the use of plasma biomarkers in clinical practice and drug trials. | * Some cases had p-tau217 concentrations below the detection limit. A more sensitive assay is needed for reliable measurements at low concentrations.  * Clinic-based cohort. | * Lack of sufficient DTA data.  * Data from this cohort have already been included in another study. |
| **Palmqvist, 2021 ^7^** | Sweden, ADNI | Plasma | Lilly | (CI to AD=102/no AD CI=441) | (CI to AD=73.2/no AD CI=71.2) | [18F]Florbetapir | * Using a model with plasma p-Tau217, cognition, and APOE yielded similar accuracy, but removing cortical thickness resulted in a slightly poorer model fit with significantly higher AUC.  * No significant differences in predictive accuracies were found while comparing plasma P-tau217 to P-tau181 for predicting progression to AD dementia within four years. | pT217 is a more accurate biomarker than pT181, which could be used to improve the diagnosis and follow-up of preclinical to advanced cases of AD. | * Lack of sufficient DTA data.  * Data from this cohort have already been included in another study. |
| **Pereira2021 ^8^** | Sweden | Plasma, CSF | Lilly | (123/67/52) | (73.9/69.2) | [18F]RO948,  [18F]Flutemetamol | * p-Tau217 is a valuable prognostic tool for estimating disease progression, improving patient management, and monitoring in clinical trials. | *Limited PET imaging.  * Clinic-based cohort.  *Small sample size. | * Lack of sufficient DTA data.  * Data from this cohort have already been included in another study. |
| **Pichet-Binette, 2022 ^9^** | Sweden | CSF | Lilly | (66/130/204) | (72.7/71.7/62.4) | [18F]Flortaucipir | * There are distinct associations between the rate of change in tau-PET and CSF p-Tau217 in the AD dementia stage. | *Short PET follow-up period.  * Clinic-based cohort.  *Small sample size. | * Lack of sufficient DTA data.  * Data from this cohort have already been included in another study. |
| **Pichet-Binette, 2022-2 ^10^** | Sweden | Plasma | Lilly | (CI to AD=26/no AD CI=84) | (CI to AD=74.77±8.12/no AD CI=71.52±8.20) | [18F]Flutemetamol | * Combining plasma p-Tau217 and cognitive composite score predicts progression to AD in MCI patients. | *Small plasma sample and no CSF sample.  * Clinic-based cohort.  *Small sample size. | * Lack of sufficient DTA data.  * Data from this cohort have already been included in another study. |
| **Smith^11^, 2022** | Sweden | Plasma, CSF | Lilly | (28/90/ NA) | 71.0 ± 8.6 | [18F]RO948 | * Tau-PET and baseline cognition provide the best prediction of cognitive decline in patients with amnestic MCI or mild dementia. | *Short follow-up period.  * Clinic-based cohort.  *Small sample size. | * Lack of sufficient DTA data.  * Data from this cohort have already been included in another study. |
| **Simren, 2022 ^12^** | Canada | CSF | Simoa | (14/10/81) | (67/73.6/72.8) | [18F]MK-6240,  [18F]AZD4694, | *Pronounced increases There is a significant difference in the levels of p-tau181 and p-Tau217 between the transentorhinal and limbic stages. | * Clinic-based cohort.  *Small sample size. | * Lack of sufficient DTA data.  * Data from this cohort have already been included in another study. |
| **Murray, 2022 ^13^** | USA | Plasma | Lilly | (9/NA/NA) + others | (87/NA/NA) | Tau PET (tracer/NA) | * The hippocampus may have a long-standing accumulation of tangles not easily detected by the p-T181 and p-T217 antibodies. | * Clinic-based cohort.  *Small sample size. | * Lack of sufficient DTA data. |
| **Montoliu-Gaya, 2022 ^14^** | Canada, Sweden | Plasma | Mass spectrometry, Simoa | (27/24/23) + Others neurodegenerative | (68/72.5/63) | [18F]MK-6240, [18F]AZD4694 | * These three plasma tau forms reflect AD-related changes in the brain, with different emergences along the AD continuum and associations with amyloid and tau pathologies.  * p-Tau217 demonstrated the most reliable diagnostic performance among all tested biomarkers. | * Clinic-based cohort.  *Small sample size. | * Lack of sufficient DTA data.  * Data from this cohort have already been included in another study. |
| **Palmqvist, 2022 ^15^** | Sweden | Plasma | Lilly | (NA/232/461) | 72.5 | [18F]Flutemetamol | * Three plasma biomarkers accurately identify Aβ positivity and predict future AD dementia. | * Many plasma p-Tau217 N-terminal and mid-domain measurements fell below the detection limit.  * Clinic-based cohort. | * Lack of sufficient DTA data.  * Data from this cohort have already been included in another study. |
| **Therriault 2022-2 ^16^** | Canada | CSF | Simoa | (65/80/179) | (66.7/70.34/71.2) | [18F]MK-6240, [18F]AZD4694 | * PET-based Braak staging can be used to model the natural history of AD and monitor its severity in living humans. It may also aid in determining eligibility for therapeutic trials. | * Studies have been conducted that examine the cases of individuals who had undergone an [18F]MK6240 scan before their death and whose bodies were later examined via autopsy.  *Limited spatial resolution of PET imaging.  * Disease staging models are artificial constructs that face sensitivity and specificity issues due to dividing continuous processes into discrete stages.  * Clinic-based cohort. | * Lack of sufficient DTA data.  * Data from this cohort have already been included in another study. |
| **Therriault 2023 ^17^** | Canada | CSF, Plasma | Simoa | (23/43/86) | (62.4/72.3/69.5) | [18F]MK-6240, [18F]AZD4694 | * Four p-tau biomarkers were found to be significantly associated with amyloid PET in two observational cohorts. The results were replicated in two independent groups with plasma and CSF p-tau biomarkers. | * The process of comprehending tau (hyper)phosphorylation is dynamic and will continue to develop as additional biomarkers become accessible.  * It is not certain if all upcoming p-tau biomarkers will exhibit the same selective correlation with amyloid PET as observed in this research.  * Clinic-based cohort. | * Lack of sufficient DTA data.  * Data from this cohort have already been included in another study. |
| **Mundada, 2023 ^18^** | USA | Plasma | Lilly | (44/53/NA) | 66.4±9.6 | [11C]PIB, [18F]Flortaucipir | * Plasma p-Tau217 and tau-PET are both biomarkers for assessing tau pathology, with the former being useful for disease severity and the latter for tracking regional-specific tau pathology. | * Clinic-based cohort.  *Small sample size. | * Lack of sufficient DTA data. |
| **Feizpour, 2023 ^19^** | Australia | Plasma | Simoa | (21/29/153)  (CI=50/CU=153) | (CI=72.5 ± 9.0/  CU=75.0 ± 5.0) | [18F]MK-6240,  [18F]NAV4694 | * Using p-Tau217 alone can reduce costs for selecting participants with MCI or mild dementia for a clinical trial designed to slow cognitive decline, compared to PET. | * Clinic-based cohort.  *Short-term follow-up.  *Small sample size. | * Lack of sufficient details related to PET.  * Data from this cohort have already been included in another study. |
| **Sanchez-Rodriguez, 2023 ^20^** | Canada | Plasma | Simoa | (16/35/81) | NA | 18F]MK-6240 | * The approach found essential links between Aβ and tau effects on cognitive impairment and neuronal excitability with disease progression. | CSF pT217/T217 and pT205/T205 showed better correlation with tau PET measures than p-tau181 concentration, indicating their potential as improved biomarkers of amyloid and tau pathology in AD. | * Lack of sufficient DTA data.  * Data from this cohort have already been included in another study. |
| **Barthelemy, 2023 ^21^** | USA, Sweden | CSF | Mass spectrometry |  | 75.5 | [18F]Flortaucipir , [18F]Flutemetamol or [18F]Florbetapir, [18F]Flortaucipir , [18F]RO948 | *CSF pT217/T217 and pT205/T205 were better correlated with tau PET measures than CSF p-tau181 concentration. These findings suggest that CSF pT217/T217 and pT205/T205 represent improved CSF biomarkers of amyloid and tau pathology in AD. | * Clinic-based cohort. | * Lack of sufficient DTA data.  * Data from this cohort have already been included in another study. |
| **Ferreira, 2023 ^22^** | Canada | Plasma | Simoa | (CI=87/CU=138) | (CI=68.6/ CU=70.3) | [18F]MK-6240,  [18F]NAV4694 | * In preclinical AD, plasma p-tau231 and p-tau217+ are state markers of Aβ, while in CI, they also inform on tau neurofibrillary tangles in the brain. | * Clinic-based cohort.  *Small sample size.  * The racial makeup of the participants is uniform. | * Lack of sufficient DTA data.  * Data from this cohort have already been included in another study. |
| **Groot, 2023 ^23^** | Sweden | CSF | Lilly | (NA/96/135) | (NA/71.8/64.6) | [18F]RO948 | * CSF p-tau217 baseline level affected temporal meta-region and Braak V-VI tau changes over time, but not cortical thickness. | * Picking regions to define Phospho-tau has limitations in generalizing results to studies using a different region for tau-PET positivity.  * Clinic-based cohort.  *Small sample size. | * Lack of sufficient DTA data.  * Data from this cohort have already been included in another study. |
| **Pichet-Binette, 2023 ^24^** | Sweden | Plasma | Lilly | (89/342/738) | 68.7±9.4 | [18F]RO948, [18F]flutemetamol | * p-Tau217 and NfL have the strongest correlation between their plasma and CSF concentrations. | * Clinic -based cohort. | * Lack of sufficient DTA data.  * Data from this cohort have already been included in another study. |
| **Horie, 2023 ^25^** | Sweden | CSF | Mass spectrometry | (102/90/160) | (72.5/71.7/70.2) | [18F]RO948 | * Bootstrapping showed that associations were significantly more vital for all participants than p-Tau217/Tau217. | * The magnitude of the trend differed between the two cohorts in some analyses, although similar trends were shown.  *Clinic-based cohort. | * Lack of sufficient DTA data.  * Data from this cohort have already been included in another study. |
| **Jack, 2024 ^26^** | USA | Plasma | Lilly | (CI=NA/CU=200) | 75 | [11C]PIB | * Plasma p-tau217 and amyloid PET can predict cognitive decline rates in cognitively unimpaired individuals at the population mean level but not at the personal level. | * Including tau PET as a predictor is an obvious question of interest that was not addressed. | * Lack of sufficient DTA data.  * Data from this cohort have already been included in another study. |
| **Therriault, 2024 ^27^** | Sweden | Plasma, CSF | Mass spectrometry, Lilly | (24/36/74) + others | (63.1/71.2/69.7) | [18F]MK-6240,  [18F]NAV4694 | * p-Tau217 has strong diagnostic performance and associations with amyloid PET and tau-PET. | *Focused on comparing immunoassays and mass spectrometry in CSF analysis for a cohort with dementia.  *Small sample size. | * Lack of sufficient DTA data.  * Data from this cohort have already been included in another study. |
| **Brum, 2024^28^** | Sweden, Canada | Plasma | Simoa | Biofinder (140/181/NA),  Triad (58/72/NA) | Biofinder (74/71/NA),  Triad (67/73/NA) | [18F]MK-6240, [18F]RO948 | *Plasma-based biomarker screening, particularly with plasma p-Tau217, can reduce unnecessary tau-PET scans by filtering out individuals with very low risk of being tau-PET positive in memory clinics. | *Clinic-based cohort. | * Lack of sufficient DTA data.  * Data from this cohort have already been included in another study. |
| **Janelidze, 2024^29^** | Sweden, USA | Plasma | Mass spectrometry, Lilly | Biofinder(CI=384/CU=495),  ADRC(CU=283) | Biofinder (CI=65/CU=68), ADRC(CU=69) | [18F]flutemetamol, [18F]AZD4694, [11C]PIB | * Blood tests that measure both p-tau217 and Aβ42/40 levels might be effective for screening CUs who show early, subthreshold signs of brain Aβ pathology, potentially aiding in the identification of candidates for future primary prevention trials in AD. | * No longitudinal plasma biomarker data.  *Clinic-based cohort. | * Lack of sufficient DTA data.  * Data from this cohort have already been included in another study. |

**References**

1. Mattsson-Carlgren N, Andersson E, Janelidze S, et al. Aβ deposition is associated with increases in soluble and phosphorylated tau that precede a positive Tau PET in Alzheimer’s disease. *Science advances*. 2020;6(16):eaaz2387.

2. Barthélemy NR, Horie K, Sato C, Bateman RJ. Blood plasma phosphorylated-tau isoforms track CNS change in Alzheimer’s disease. *Journal of Experimental Medicine*. 2020;217(11):e20200861.

3. Barthélemy NR, Bateman RJ, Hirtz C, et al. Cerebrospinal fluid phospho-tau T217 outperforms T181 as a biomarker for the differential diagnosis of Alzheimer's disease and PET amyloid-positive patient identification. *Alzheimers Res Ther*. Mar 17 2020;12(1):26. doi:10.1186/s13195-020-00596-4

4. Brickman AM, Manly JJ, Honig LS, et al. Plasma p‐tau181, p‐tau217, and other blood‐based Alzheimer's disease biomarkers in a multi‐ethnic, community study. *Alzheimer's & Dementia*. 2021;17(8):1353-1364.

5. Lantero‐Rodriguez J, Snellman A, Benedet AL, et al. P‐tau235: a novel biomarker for staging preclinical Alzheimer’s disease. *EMBO Molecular Medicine*. 2021;13(12):e15098.

6. Janelidze S, Palmqvist S, Leuzy A, et al. Detecting amyloid positivity in early Alzheimer's disease using combinations of plasma Aβ42/Aβ40 and p‐tau. *Alzheimer's & Dementia*. 2022;18(2):283-293.

7. Palmqvist S, Tideman P, Cullen N, et al. Prediction of future Alzheimer’s disease dementia using plasma phospho-tau combined with other accessible measures. *Nature Medicine*. 2021;27(6):1034-1042.

8. Pereira JB, Janelidze S, Stomrud E, et al. Plasma markers predict changes in amyloid, tau, atrophy and cognition in non-demented subjects. *Brain*. 2021;144(9):2826-2836.

9. Pichet Binette A, Franzmeier N, Spotorno N, et al. Amyloid-associated increases in soluble tau relate to tau aggregation rates and cognitive decline in early Alzheimer’s disease. *Nature Communications*. 2022;13(1):6635.

10. Pichet Binette A, Palmqvist S, Bali D, et al. Combining plasma phospho-tau and accessible measures to evaluate progression to Alzheimer’s dementia in mild cognitive impairment patients. *Alzheimer's Research & Therapy*. 2022;14(1):46.

11. Smith R, Cullen NC, Pichet Binette A, et al. Tau‐PET is superior to phospho‐tau when predicting cognitive decline in symptomatic AD patients. *Alzheimer's & Dementia*. 2023;19(6):2497-2507.

12. Simrén J, Brum WS, Ashton NJ, et al. CSF tau368/total-tau ratio reflects cognitive performance and neocortical tau better compared to p-tau181 and p-tau217 in cognitively impaired individuals. *Alzheimer's Research & Therapy*. 2022;14(1):1-12.

13. Murray ME, Moloney CM, Kouri N, et al. Global neuropathologic severity of Alzheimer’s disease and locus coeruleus vulnerability influences plasma phosphorylated tau levels. *Molecular neurodegeneration*. 2022;17(1):85.

14. Montoliu-Gaya L, Benedet AL, Tissot C, et al. Mass spectrometric simultaneous quantification of tau species in plasma shows differential associations with amyloid and tau pathologies. *Nature Aging*. 2023:1-9.

15. Palmqvist S, Stomrud E, Cullen N, et al. An accurate fully automated panel of plasma biomarkers for Alzheimer's disease. *Alzheimer's & Dementia*. 2023;19(4):1204-1215.

16. Therriault J, Pascoal TA, Lussier FZ, et al. Biomarker modeling of Alzheimer’s disease using PET-based Braak staging. *Nature aging*. 2022;2(6):526-535.

17. Therriault J, Vermeiren M, Servaes S, et al. Association of phosphorylated tau biomarkers with amyloid positron emission tomography vs tau positron emission tomography. *JAMA neurology*. 2023;80(2):188-199.

18. Mundada NS, Rojas JC, Vandevrede L, et al. Head-to-head comparison between plasma p-tau217 and flortaucipir-PET in amyloid-positive patients with cognitive impairment. *Alzheimer's Research & Therapy*. 2023;15(1):157.

19. Feizpour A, Doré V, Doecke JD, et al. Two-year prognostic utility of plasma p217+ tau across the Alzheimer’s continuum. 2023;

20. Sanchez-Rodriguez LM, Bezgin G, Carbonell F, et al. Revealing the combined roles of Aβ and tau in Alzheimer’s disease via a pathophysiological activity decoder. *Biorxiv*. 2023;

21. Barthélemy NR, Saef B, Li Y, et al. CSF tau phosphorylation occupancies at T217 and T205 represent improved biomarkers of amyloid and tau pathology in Alzheimer’s disease. *Nature Aging*. 2023/04/01 2023;3(4):391-401. doi:10.1038/s43587-023-00380-7

22. Ferreira PC, Therriault J, Tissot C, et al. Plasma p‐tau231 and p‐tau217 inform on tau tangles aggregation in cognitively impaired individuals. *Alzheimer's & Dementia*. 2023;19(10):4463-4474.

23. Groot C, Smith R, Stomrud E, et al. Phospho-tau with subthreshold tau-PET predicts increased tau accumulation rates in amyloid-positive individuals. *Brain*. Apr 19 2023;146(4):1580-1591. doi:10.1093/brain/awac329

24. Pichet Binette A, Janelidze S, Cullen N, et al. Confounding factors of Alzheimer's disease plasma biomarkers and their impact on clinical performance. *Alzheimer's & Dementia*. 2023;19(4):1403-1414.

25. Horie K, Salvadó G, Barthélemy NR, et al. CSF MTBR-tau243 is a specific biomarker of tau tangle pathology in Alzheimer’s disease. *Nature medicine*. 2023;29(8):1954-1963.

26. Jack Jr CR, Wiste HJ, Algeciras‐Schimnich A, et al. Comparison of plasma biomarkers and amyloid PET for predicting memory decline in cognitively unimpaired individuals. *Alzheimer's & Dementia*. 2024;

27. Therriault J, Woo MS, Salvadó G, et al. Comparison of immunoassay-with mass spectrometry-derived p-tau quantification for the detection of Alzheimer’s disease pathology. *Molecular Neurodegeneration*. 2024;19(1):2.

28. Brum WS, Cullen NC, Therriault J, et al. A blood-based biomarker workflow for optimal tau-PET referral in memory clinic settings. *Nat Commun*. Mar 14 2024;15(1):2311. doi:10.1038/s41467-024-46603-2

29. Janelidze S, Barthélemy NR, Salvadó G, et al. Plasma Phosphorylated Tau 217 and Aβ42/40 to Predict Early Brain Aβ Accumulation in People Without Cognitive Impairment. *JAMA Neurol*. Jul 28 2024;doi:10.1001/jamaneurol.2024.2619

30. Jack Jr CR, Andrews SJ, Beach TG, et al. Revised criteria for the diagnosis and staging of Alzheimer’s disease. *Nature medicine*. 2024:1-4.
